# The ADePT framework for assessing autonomous laboratory robotics

**DOI:** 10.1038/s42004-026-01932-9

**Published:** 2026-02-20

**Authors:** Pablo Salazar-Villacis, Brahim Benyahia

**Affiliations:** 1 School of AACME, Loughborough University, Loughborough, UK; 2 Department of Chemical Engineering, Loughborough University, Loughborough, UK

**Keywords:** Analytical chemistry, Chemical engineering

## Abstract

Laboratory robotics is advancing from routine automation toward autonomous systems capable of intelligent decision-making and flexible execution. This perspective outlines key milestones and introduces the ADePT framework, which defines four core dimensions of robotic capability proficiency: adaptability and learning, dexterity, perception, and task complexity. We discuss future directions for self-driving laboratories, including robot-centric, end-to-end robotic integration, and collaborative human–robot environments. These scenarios highlight the importance of technological enablers and evolving regulatory paradigms. By connecting present technologies to emerging system configurations, this work offers a foundation for designing autonomous laboratory ecosystems that support scientific discovery and operational efficiency.

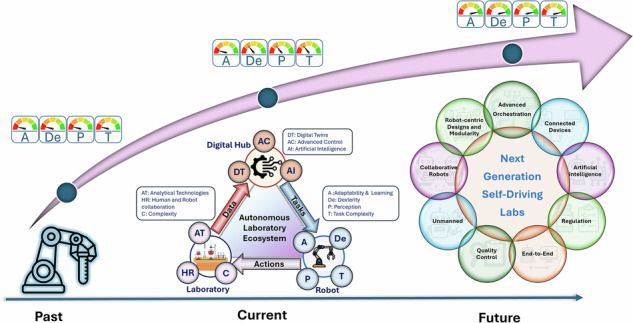

## Abstract (web summary)

Laboratory robotics is shifting from scripted automation towards autonomous systems that can perceive, decide and act robustly in real experimental environments. Here, the authors introduce the ADePT framework, comprising adaptability and learning, dexterity, perception and task complexity, to benchmark robotic capability, expose key bottlenecks and chart practical routes towards truly self-driving laboratories.

## Introduction

The automation of laboratory routines and processes has long been a cornerstone in the pursuit of efficiency, precision, and reproducibility in scientific research^1–3^. From the early days of industrial robots performing repetitive tasks to the current sophisticated autonomous systems, laboratory robotics has undergone a significant transformation to meet the unprecedented demand for rapid solutions to ongoing scientific challenges^4^. Recent strides are being driven by digital transformation and the integration of smart sensing technologies, artificial intelligence and machine learning, advances in mechanical design and additive manufacturing, and closed-loop feedback control. These developments are enhancing the capabilities of robots in laboratory settings, where greater dexterity, precision, and resilience enable the execution of increasingly complex tasks. At the heart of this transition is the emerging concept of the autonomous laboratory ecosystem, a modular, intelligent, and self-adaptive framework in which robots not only execute experiments but also perceive, decide, and adapt within dynamic scientific workflows^3,5–8^.

Catalysed by Industry 4.0^9,10^ and the human-centred emphasis of Industry 5.0^11,12^, the past few years have seen rapid growth in laboratory robotics across research and industrial settings (Supplementary Material, section SM1). The literature is articulated around four themes: (i) closed-loop optimisation and self-driving laboratories^13–15^, (ii) multimodal perception combining vision, force, and tactile sensing^16,17^, (iii) skilled, contact-rich and reconfigurable manipulation^18,19^, and (iv) interoperability and orchestration that link instruments and data^3,5,20,21^. These advances increase throughput and reproducibility but also expose mismatches between sensing, manipulation and learning capability and the task complexity typical of modern laboratories. Standardisation is a precondition for that orchestration: laboratory-level command schemas such as SiLA 2 (Standardisation in Lab Automation) and information-model-based integration via OPC UA (OPC Unified Architecture; OPC originally “OLE for Process Control”, later “Open Platform Communications”) have emerged as practical routes to cross-vendor control and data exchange in research labs^22,23^. Digital twins and high-fidelity simulation are emerging as cross-cutting technologies for robotic systems and experimentation supporting calibration, fault detection, maintenance, self-optimisation and streamlining decision making, while human-in-the-loop strategies are increasingly recognised as essential for safe, adaptable operation in exploratory research^24–30^.

Many competences now associated with autonomous laboratories were already present decades ago. Laboratory robots integrated with analytical instruments and specimen identification, used liquid-level and contact cues for handling, and coordinated workflows in which closed-loop control of variables such as temperature, flow, and pH was implemented at the instrument level^31,32^. By contrast, broad task generalisation beyond fixed scripts matured later and was not commonplace in early laboratory cells^4^. Early impact was further constrained by gaps in standardisation, fragmented software and limited support for modular design, which kept laboratory robotics functionally siloed outside industrial settings^6,23,33,34^. These lessons motivate the capability-based view we adopt later, where we examine whether sensing, manipulation, learning and task complexity are appropriately matched to the demands of chemical self-driving labs.

As laboratory workflows become more complex and variable, reliance on fixed, pre-programmed sequences limits reliability and scope. Modern laboratories now require platforms that adapt across tasks, handle delicate manipulations, and operate safely alongside researchers. These demands are driving a shift from rigid, task-specific machines to systems that perceive, plan and learn under uncertainty, enabling more autonomous decision-making. Within this context, the autonomous laboratory ecosystem provides a useful organising concept for rethinking how experiments are designed, executed and iterated^3,5,6,8,35^.

Recent studies have integrated cutting-edge technologies to enhance robotic capability in laboratory settings. Markerless 3D perception enables detection of transparent glassware and more reliable interaction in typical laboratory environments^16^. Systems that combine visual, force, and tactile feedback improve the precision of vial insertion and similar contact-rich tasks^17^. Learning methods, particularly reinforcement learning, expand the robot’s capacity to benefit from prior and ongoing experience and to adapt across tasks. One implementation with domain randomisation enabled precise powder weighing across diverse materials and target masses^36^. The integration of large language models further advances adaptability by allowing robots to interpret natural language instructions and compose appropriate action sequences^16,37,38^.

Enhancements in mechanical design, including increased degrees of freedom together with force and tactile sensing, have improved dexterity for tasks such as precise pouring and microwell plate insertion^19,39^. Managing complex, multi-step workflows benefits from task-and-motion planning frameworks, such as PDDLStream, which generate feasible action sequences under constraints like collision avoidance and resource availability, and from their coupling to data-driven models^16,38,40^.

This Perspective proposes a more comprehensive and rigorous evaluation of robotic capabilities within laboratory environments and introduces four core dimensions that underpin the development of effective autonomous robotic systems: Adaptability and Learning, Dexterity, Perception, and Task Complexity, which together form the basis of the ADePT framework. The term “adept” implies skill and proficiency, qualities essential for robotic platforms operating in complex and evolving scientific environments. By establishing a systematic and quantitative approach to evaluating these core capabilities, we aim to set a benchmark that helps identify current challenges and guide future directions. In doing so, we lay the foundation for a shift not only in laboratory operations and scientific discovery but also in the design of next-generation laboratories and analytical technologies.

The Outlook explores three emerging and potentially overlapping visions for the future of laboratory robotics. The first is the robot-exclusive laboratory, where all experimental and operational tasks are performed by robots in unmanned modular environments, while a human-in-loop may still be implemented outside the laboratory environment for high-level decisions and supervision. The second envisions end-to-end robotic integration, in which robotic systems seamlessly connect research, development, manufacturing, and quality control. The third outlines collaborative human–robot laboratories, where flexible robotic systems operate safely alongside researchers. These trajectories illustrate the diverse paths toward more intelligent, connected, and autonomous laboratory ecosystems.

## Historical overview

The development of laboratory robotics reflects the broader evolution of automation from rigid, task-specific devices to increasingly intelligent and adaptive systems. In the 1980s and 1990s, the first generation of laboratory automation tools emerged through the adaptation of industrial robotic arms to laboratory scales. Early cylindrical and Cartesian robots were deployed for benchtop tasks such as sample transfer, reagent dosing, and integration with online analysis^31,32^. These systems were suitable for manipulating reaction vessels ranging from 5 to 30 ml and reproduced repetitive manual procedures with high precision. They featured interchangeable tooling, such as rotating grippers and automated syringes, and in some cases basic force sensing for grip validation and collision detection. Some were enclosed in dedicated stations to deliver reliable performance with minimal programming.

Although technically sound, these systems were rarely general-purpose and saw limited adoption. Their potential was constrained by high costs, a lack of flexibility, and limited ability to adapt to changing experimental procedures^41^. Despite exhibiting traits that now define collaborative robots, their development stalled. With sustained investment, these early benchtop systems could have evolved into modular and adaptable robotic platforms, well ahead of their time.

From the 1990s through the early 2000s, laboratory robotics saw more extensive deployment, particularly in industrial and pharmaceutical contexts. This era included the use of SCARA arms and articulated robots with five or six axes of motion, often mounted on linear rails to extend their operational reach across interconnected instruments. These robots were typically responsible for transporting microplates between unit operations, such as incubators, readers, and liquid handlers. Their integration into automated high-throughput screening workflows enabled continuous, unattended operation across many experimental cycles. However, the need for safety enclosures, custom grippers, and precise calibration limited their flexibility. Most systems were purpose-built for specific tasks and operated as closed automation cells, which restricted reusability and hindered integration into dynamic or academic research settings.

This rise in throughput came at the cost of adaptability. While the deployment of fixed-format liquid handling platforms and microplate robotics led to significant gains in efficiency, particularly in drug discovery, it also reinforced a fragmented approach to automation. Systems were rarely interoperable and often relied on vendor-specific hardware and software, complicating modifications or upgrades. The focus remained on performance within defined workflows, rather than on creating general-purpose or modular platforms^42–44^.

A conceptual breakthrough occurred with the emergence of robot scientists^45^. These systems aimed not only to automate physical tasks but also to incorporate reasoning, learning, and hypothesis-driven experimentation. Adam, introduced in 2009, was the first robot to autonomously generate and test scientific hypotheses, discovering gene–enzyme relationships in yeast metabolism^46^. Eve, introduced in 2015, extended this model to early-stage drug discovery, combining compound screening with iterative modelling to identify candidate molecules and predict their activity^47^. These platforms demonstrated that automation could go beyond execution and begin to emulate aspects of scientific thinking^48^.

**Fig. 1:**
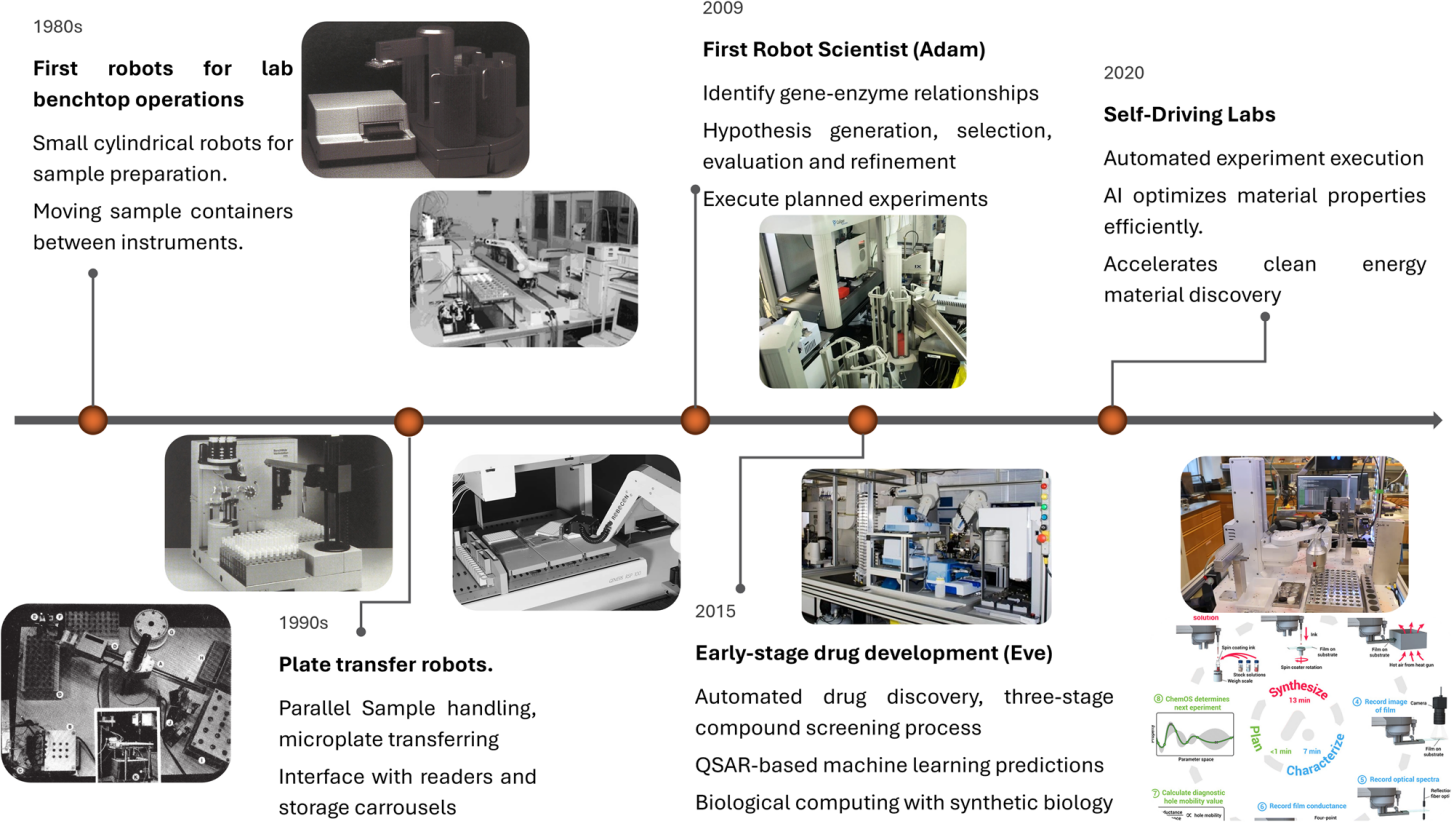
Representative milestones in the evolution of laboratory robotics from the 1980s to 2020. Early laboratory automation focused on fixed, benchtop robotic systems for repetitive operations such as sample preparation and plate transfer (1980s–1990s). Subsequent advances enabled integrated robotic workcells for high-throughput screening and automated chemical synthesis, incorporating coordinated liquid handling, sample storage, and analytical interfaces (1990s–2000s). More recent systems combined robotics with computational planning, machine learning, and closed-loop experimentation to support hypothesis generation, adaptive experiment execution, and materials discovery, culminating in self-driving laboratories capable of autonomous optimisation and accelerated scientific discovery (2010s–2020s). Reproduced with permission from Frisbee et al. (1984), © American Chemical Society; Timoney and Felder (1998), © Elsevier; Okamoto and Deuchi (2000), © John Wiley & Sons; Sparkes et al. (2010), CC BY 2.0; MacLeod et al. (2020), © AAAS.

The convergence of machine learning frameworks, cloud computing, and accessible sensors during the late 2010s catalysed a new generation of robotic platforms. Among these, a self-driving laboratory known as Ada exemplified a modular system that autonomously planned, executed, and optimised thin-film materials experiments using model-based algorithms^49^. Unlike earlier platforms, Ada generalised across experimental contexts and improved its performance iteratively using real-time data. This evolution is summarised in Fig. 1, which outlines representative platforms from early benchtop automation to the emergence of robot scientists and fully self-driving laboratories.

This historical progression highlights how many of the core capabilities seen in current autonomous systems, such as sensor integration, feedback control, and task generalisation, were already technically feasible decades ago. Their limited early impact stemmed more from systemic factors, including the lack of standardisation, fragmented software ecosystems, and the absence of long-term support for modular design. As a result, robotics in the laboratory remained functionally siloed and underexploited outside of industrial settings.

The trajectory of laboratory robotics reveals both achievements and missed opportunities. It illustrates that progress depends not only on technical capability but also on alignment with the evolving needs of experimental science. The early discontinuation of collaborative, benchtop robots, for example, arguably delayed the transition to more flexible and general-purpose platforms. Recognising this history is essential for guiding the development of next-generation laboratory automation that is not only intelligent and precise but also adaptable, interoperable, and open to continuous evolution.

## Recent progress in laboratory robotics

Laboratory robotics has progressed from simple automation of repetitive tasks to becoming a core component of autonomous experimentation platforms. In these systems, robots serve primarily as physical executors, transferring vials, plates, and reagents with precision and reliability. While decision-making and optimisation are typically handled by integrated experimental planning algorithms, robotics enables the realisation of these plans in the physical world, bridging analytical feedback with experimental action in closed-loop workflows.

This section reviews the current capabilities of laboratory robots, beginning with their role as systems integrators and progressing to the technical components that support their increasing autonomy: Adaptability and Learning, Dexterity, Perception, and Task Complexity. These aspects are central to enabling robots to operate robustly within dynamic laboratory settings. By examining each in detail, we provide a framework for understanding how physical execution capabilities are evolving to support more intelligent, efficient, and flexible laboratory automation. For transparency, we summarise headline observations in the main text and provide the complete ADePT scoring and evidence for each case in SM3.1 (systems integrators) and SM3.2 (key features).

### Robots as systems integrators

Laboratory robots have long served as systems integrators, physically linking instruments, modules, and procedures across experimental workflows. While historically their role was limited to transporting objects such as vials, microplates, flasks, and other labware, recent advances have embedded them in closed-loop platforms where the orchestration of experiments is guided by real-time analytical feedback. Within such platforms, the robot executes the physical tasks that constitute the experiment, while the decision-making regarding what to do next is driven by algorithms that optimise predefined scientific objectives. This integration of hardware and digital logic marks a critical departure from traditional laboratory automation.

**Fig. 2:**
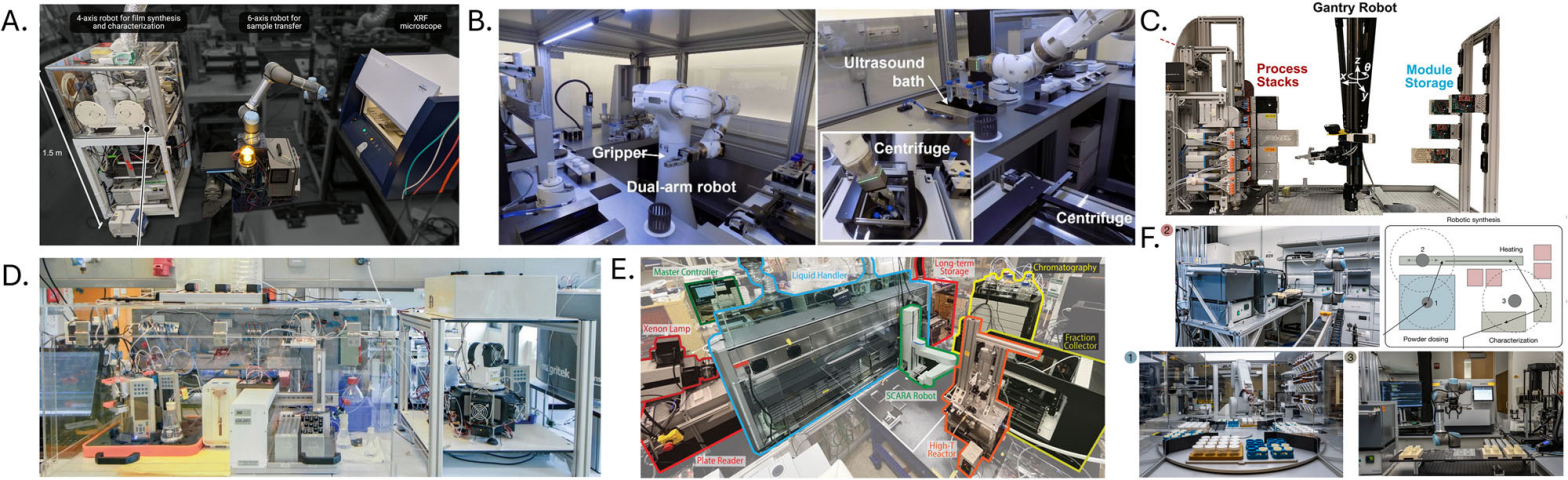
Robotic systems as integrators in lab automation. **A** Four- and six-axis robotic platforms for autonomous materials synthesis and sample transfer. **B** Dual-arm robotic system integrating ultrasound and centrifugation for automated sample preparation. **C** Gantry-based robot enabling modular, multistep flow synthesis. **D** Automated platform for closed-loop optimisation of photocatalytic reactions. **E** Integrated system combining robotic synthesis with computational modelling for molecular discovery. **F** Robotic laboratory platform supporting heating, dosing and in situ characterisation. Panels reproduced with permission from MacLeod et al. (2022), Dembski et al. (2023) and Szymanski et al. (2023) under CC BY licences; from Nambiar et al. (2022) with permission from the American Chemical Society; and from Slattery et al. (2024) and Koscher et al. (2023) with permission from the American Association for the Advancement of Science (AAAS).

Robotic integration plays a central role in enabling self-driving experimentation. In chemical synthesis, a small number of landmark studies have demonstrated the use of robots to automate complex multistep processes. One study reported the first implementation of a six-degree-of-freedom UR5e robotic arm to assemble modular flow reactors for reactions suggested by retrosynthetic planning^50^. In a subsequent study, the UR5e was replaced with a gantry robot equipped with a rotary end-effector, extending workspace coverage and improving reliability in routing^51^. This system also integrated process analytical technologies to enable closed-loop optimisation of yield in a multistep reaction sequence (Fig. 2C).

Figure 2 presents a set of representative robotic platforms that serve integrative roles across domains such as flow chemistry, nanoparticle synthesis, and materials characterisation. These examples highlight the breadth of robotic applications in experimental coordination.

In materials science, a closed-loop laboratory was developed in which robotic arms performed aspirating, dispensing, and transferring operations between synthesis and characterisation modules^49,52^ (Fig. 2A). Though actions were pre-scripted, they were informed by analytical feedback, enabling iterative optimisation of material properties. Similarly, an automated platform for the synthesis of inorganic materials was constructed, with a robot coordinating heating, dosing, and characterisation tasks^53^ (Fig. 2F). In both cases, the robots operated within well-defined environments and did not possess autonomous task-level flexibility.

In nanoparticle synthesis, a dual-arm system was implemented to perform coordinated dosing, mixing, and centrifugation across modular synthesis units^54^ (Fig. 2B). While the platform demonstrated versatility in manipulating a variety of laboratory tools, it relied on predefined procedures and required structured spatial arrangements to function reliably.

In photocatalysis, the RoboChem platform was developed to integrate a liquid handler, syringe pumps, and a flow photoreactor with in-line NMR to autonomously optimise reaction conditions^55^ (Fig. 2D). The system applies a Bayesian optimisation algorithm to adaptively explore a high-dimensional chemical space, identifying conditions that maximise yield or throughput. RoboChem does not incorporate a robotic arm. All experimental steps are executed through fixed-position fluid handling components. Although highly effective in linking data and action, the system’s hardware remains limited in spatial flexibility and cannot autonomously adapt to task reconfiguration.

Robotic systems are also used in high-throughput and sample preparation contexts. A dual-arm robot was deployed for automating solid-phase extraction^56^, while robotic pipetting was implemented for preparing samples for X-ray photon correlation spectroscopy^57^. Robotic microplate handling has also been used to synthesise and characterise colloidal nanocrystals^58^. These platforms emphasise precision and consistency, though they typically operate within narrowly defined task spaces.

In automated discovery pipelines, predictive modelling was combined with robotic synthesis and characterisation in a molecular discovery workflow^59^ (Fig. 2E). Here, robots executed prescribed experimental actions informed by model outputs. While effective, the robotic execution was limited to a fixed set of predefined tasks and did not support real-time recovery from failure or generalisation across workflows.

Across these examples, robotic platforms have delivered gains in speed, precision and reproducibility, yet most deployments still operate as structured automation with little perception, adaptation or autonomous error handling. Their roles are tightly coupled to task scripts and spatial layouts, with limited capacity to interpret or respond to variation in their environment^4,60^. Full ADePT evaluations for these systems-integrator cases are provided in Supplementary Material SM3.1, scored using the rubric in SM2.

Robots, therefore, serve reliably as systems integrators in controlled laboratory settings. They enable the physical execution of algorithmically guided experiments and contribute to the broader architecture of self-driving laboratories, but their function remains bounded by pre-programmed actions within fixed systems. Transitioning from integration to autonomy requires advances in perception, learning, dexterity and the ability to manage complex, variable tasks, as highlighted in recent overviews of human-in-the-loop and closed-loop laboratory practice^3,61^. These components are addressed in the following sections.

Emerging open interfaces are lowering adoption barriers and making heterogeneous devices easier to orchestrate in practice. In laboratory contexts, SiLA 2 has matured into a vendor-neutral command schema with open-source tooling that eases driver development and device discovery while supporting reproducibility and auditability within workflow engines^23,62^. These developments directly address legacy lock-in and reduce the bespoke glue code that historically hindered modular deployments.

Important challenges remain. Many instruments still lack mature information models, version drift complicates long-term maintenance, and mapping legacy telemetry to common schemas is labour-intensive; recent studies show that automated generation and reuse of OPC UA information models and event-stream analysis can lower these costs and improve traceability in multi-tool labs^63,64^. Evidence from life-science deployments further indicates that layering laboratory device profiles on OPC UA simplifies plug-and-play orchestration and lifecycle support^62^.

Complementary industrial standards provide secure, model-based communication that scales from benchtop instruments to plant-level systems. OPC UA information models support long-lived interoperability, and when combined with Time-Sensitive Networking and modern workflow managers, they enable laboratories to integrate legacy equipment while achieving robust, traceable execution across platforms^63,65,66^.

Alongside these interoperability layers, researchers are adopting experiment-centric digital twins that encode reaction and materials-process models to prioritise experiments, explore design spaces in silico and de-risk closed-loop optimisation before execution, with chemistry and crystallisation case studies demonstrating mechanistic model-based digital twins coupled to model-predictive control and uncertainty-aware design frameworks^67–72^. Complementing these high-fidelity twins, the frugal twin concept uses low-cost surrogates to emulate key behaviours of self-driving laboratories for safe training, benchmarking and protocol development^73^.

A complementary device-level strand proposes plug-and-play laboratory twins that store device geometric features and robot approach frames to reduce teaching and speed integration in self-driving workcells^74^. At the task level, learning from simulation for laboratory activities such as powder weighing provides a concrete bridge from twin-based training to reliable physical execution in materials chemistry contexts^36^. These developments are presently used chiefly for planning and risk management rather than continuous bi-directional control; coupling twin state to provenance-aware workflow engines and live laboratory telemetry would move systems-integrator robots beyond pre-programmed routines and motivate the adoption of the ADePT capabilities in the system^4,25,75^

### Key features of robot proficiency: the ADePT framework

The transition from structured automation to autonomous laboratory systems requires robots to operate beyond fixed routines and static environments. The key features of robot proficiency can be captured by the ADePT framework: Adaptability and Learning, Dexterity, Perception, and Task Complexity. These dimensions represent the essential capabilities that enable robots to interpret their surroundings, respond to variation, manipulate laboratory equipment with precision, and perform complex, multi-step procedures (Supplementary Material, section SM2). Interoperability is orthogonal to ADePT’s capability scores but underpins their practical realisation; we discuss concrete standards and integration patterns in the Outlook^22,23^. Per-study ADePT evaluations linked to this section are compiled in SM3.2. Rather than acting solely as task executors, autonomous robots are becoming integral to closed-loop experimental platforms, where physical actions are shaped by analytical feedback and algorithmic planning. This framework provides a basis for examining recent developments in laboratory robotics, supported by representative case studies that illustrate current advances and highlight the remaining challenges to broader deployment in research and chemical engineering contexts. Readers wishing to view the individual scores and justifications can consult the entries in SM3.2 and the cross-study comparison in SM4.

#### Adaptability and learning

Learning and adaptability are critical enablers of autonomy in robotic systems, especially in laboratories where tasks, materials, and conditions frequently vary^4,53,76^. While conventional automation excels at repeating known routines, autonomous laboratories require robots to generalise across tasks, respond to novel inputs, and refine their behaviour based on experience^77–79^. Progress in machine learning, large language models, and sensor-driven feedback is making this shift increasingly feasible. Scoring definitions for this component are specified in SM2 (subsection SM2.1), with case-by-case justifications in SM3.2.

##### Advanced learning methods

**Fig. 3:**
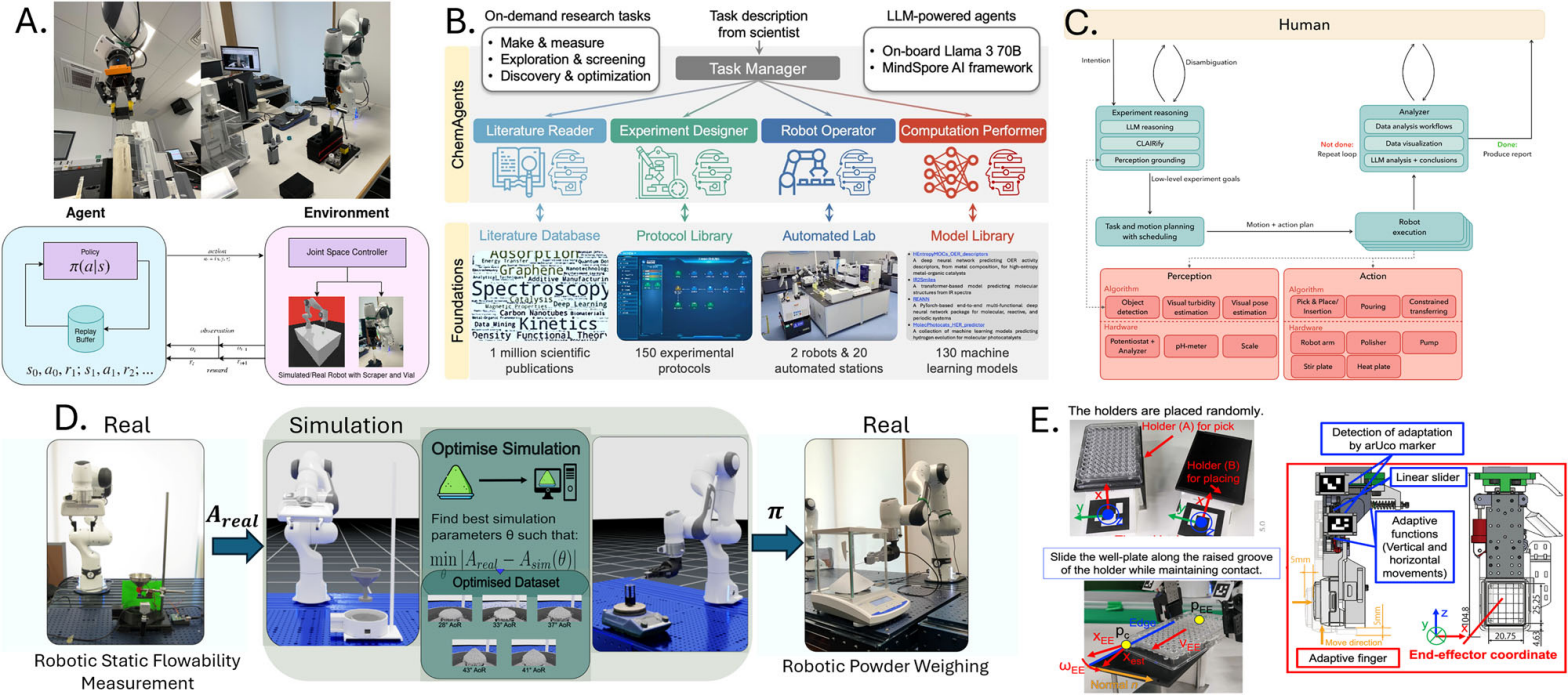
Adaptive learning and sensory feedback in robotics for lab automation. **A** Reinforcement learning with curriculum-based task progression for adaptive scraping. **B** LLM-driven multi-agent architecture coordinating experimental design, robotic execution and computation. **C** LLM-enabled robotic system integrating perception, planning and execution for real-time adaptation in chemistry workflows. **D** Flowability-informed reinforcement learning (FLIP) enabling adaptive robotic powder weighing across materials and target masses. **E** Sensory-feedback-driven control for precise well-plate insertion under variable alignment conditions. **A**, **D** and **E** reproduced from arXiv preprints under a Creative Commons Attribution 4.0 (CC BY 4.0) licence. **B** and **C** reproduced with permission from Song et al. (2025), © American Chemical Society, and Darvish et al. (2024), © Elsevier.

Recent advances in reinforcement learning (RL) have enabled robots to adapt to uncertainty and task variability in laboratory settings. For powder handling, the FLIP framework integrates measured material flowability into simulation and curriculum-based RL, enabling a robotic system to achieve milligram-level weighing accuracy across diverse powders and previously unseen target masses^80^ (Fig. 3D). Curriculum-based RL has also been implemented to train a robot in powder scraping, gradually increasing task complexity to build robustness in variable vial conditions and tool geometries^17^ (Fig. 3A). These systems show how trial-and-error training, when coupled with task simulation, can produce reliable laboratory behaviours.

##### Language models and symbolic reasoning

Large language models (LLMs) are expanding how robots interpret and respond to high-level task instructions. LLMs have been used to generate symbolic representations of chemical procedures, enabling robots to perform varied tasks such as solubility screening and sample preparation through natural language prompts^16,37^ (Fig. 3C). In another approach, LLMs were combined with a multi-agent architecture to coordinate task planning across up to 20 automated stations^38^ (Fig. 3B). These strategies reduce the need for manual coding and facilitate broader adoption of robotic systems in laboratory research. However, current LLMs struggle with grounded reasoning and context retention, and their application in safety-critical settings like chemical engineering remains constrained by reliability concerns.

##### Adaptation efficiency and real-time adjustments

Robots must respond to variation during task execution, not just across training episodes. One system demonstrated this capability by adapting vial insertion trajectories in real time based on force and tactile feedback^17^. Sub-millimetre alignment during well-plate insertion has also been achieved using real-time adjustment of end-effector orientation and pressure^19^ (Fig. 3E). These feedback loops allow robots to compensate for minor misalignments, improving reliability in workflows where precision handling is critical. This capability is particularly relevant to chemical engineering, where tasks such as sealing, filtering, or pipetting must be executed with minimal variation to maintain experimental integrity.

##### Generalisation across tasks

The ability to generalise skills across experimental protocols is a key step toward more flexible automation. Combining learning methods with adaptable hardware has enabled a single robot to perform multiple chemistry tasks within a unified framework^16^. By integrating learning with task and motion planning, such systems can adjust procedures in response to environmental feedback. However, many systems still perform well only in tightly defined settings, limiting their deployment in more variable or open-ended laboratory contexts^81^.

Despite rapid progress, significant challenges remain. Transfer learning between simulation and real-world scenarios is complex due to the reality gap, where models trained in virtual environments may fail to perform reliably under physical constraints. Digital twin environments can accelerate adaptation by allowing policy updates and identification routines to be exercised under controlled distribution shifts such as layout changes, batch variation and sensor noise, improving sample efficiency and reducing risky on-hardware exploration. Studies in robotics and chemical automation outline how simulation-to-real transfer and twin-in-the-loop optimisation shorten learning cycles and support safer online updates in closed-loop experiments^4,82,83^. Ensuring safety and robustness in continuous learning processes is particularly important in laboratory contexts, where execution errors can result in costly or hazardous outcomes. These concerns are especially critical in chemical engineering, where material behaviour, environmental factors, and process control impose strict reliability requirements on autonomous systems.

#### Dexterity

Dexterity defines a robot’s ability to manipulate diverse objects with precision and control, a requirement central to many laboratory procedures^84,85^. In chemical settings, robots must handle vials, pipettes, flasks, and specialised labware with care, often within confined spaces or under delicate constraints^86,87^. As autonomous systems take on more complex workflows, dexterity becomes a defining factor in enabling reliable and generalisable physical interaction; see SM2.2 for the Dexterity rubric and thresholds, with supporting case assessments in SM3.2^85,88^.

##### Degrees of freedom

**Fig. 4:**
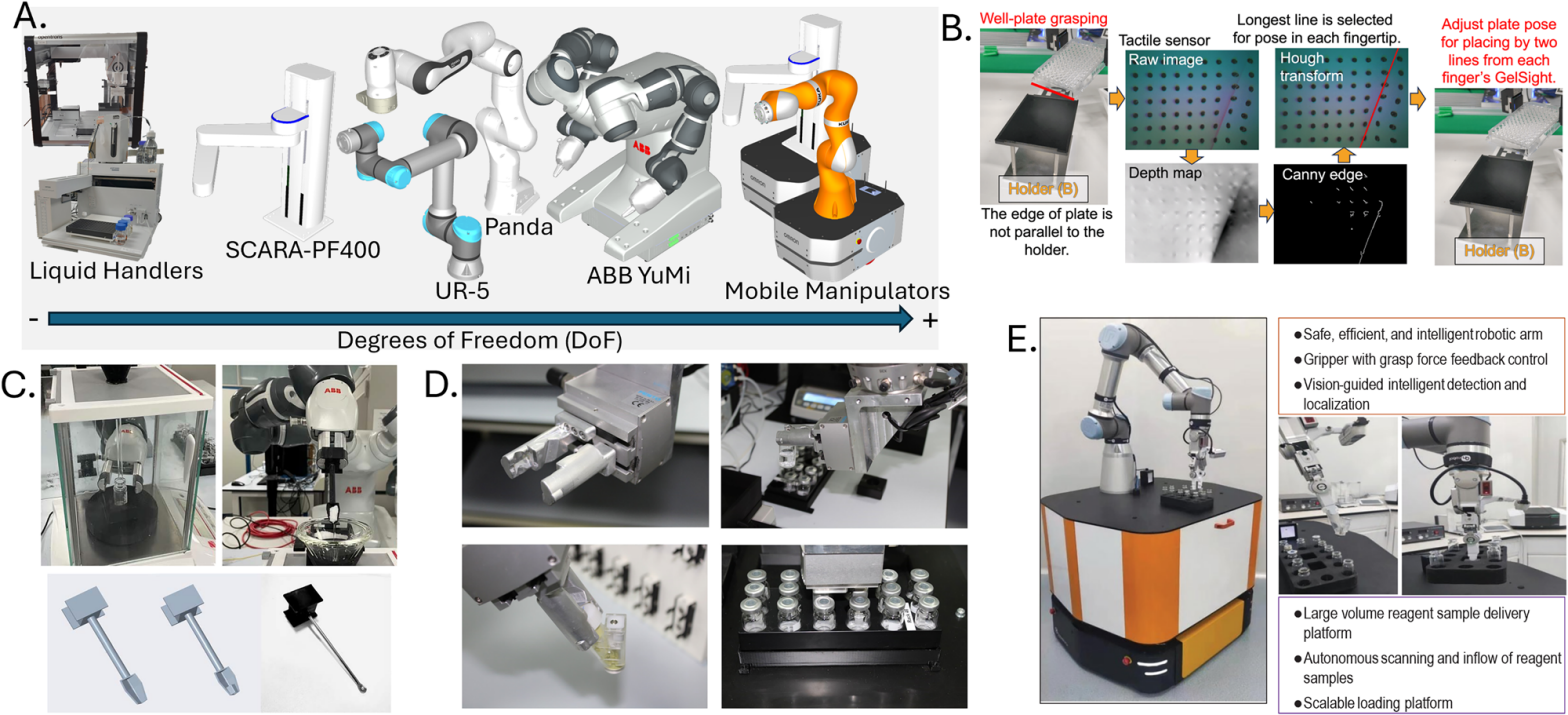
Robotic systems demonstrating dexterity in laboratory tasks. **A** Robotic platforms spanning a range of degrees of freedom, from liquid handlers to mobile manipulators, enabling flexible laboratory handling. **B** Force, torque and tactile sensing enabling precise well-plate insertion under variable alignment conditions. **C** Customised spatulas and end-effectors for controlled material manipulation. **D** Gripper designs with geometric features to ensure secure handling of laboratory containers. **E** Integrated visual and force feedback enabling adaptable vial manipulation during laboratory tasks. **B** reproduced from arXiv preprints under a Creative Commons Attribution 4.0 (CC BY 4.0) licence. **C** reproduced under a Creative Commons Attribution 3.0 (CC BY 3.0) licence. **D** reproduced from Burger et al. (2020), © Springer Nature. **E** reproduced with permission from Zhu et al. (2022), © Elsevier.

A key enabler of dexterity is the number and configuration of joints in the robotic arm. Robotic systems such as the ABB YuMi^39,89^ and Franka Emika Panda^16,17,19,89,90^ feature seven degrees of freedom, allowing for complex movements like vial insertion, pipetting, or precise pouring in confined environments (Fig. 4A). These configurations enable human-like reach and orientation control. Mobile manipulators extend this capability across space. Six-degree-of-freedom UR5e arms have been mounted on omnidirectional platforms to interact with equipment across different workstations^38,91^. Similarly, a four-degree-of-freedom arm has been combined with a mobile base to increase workspace coverage^92^. However, many platforms still rely on fixed trajectories and lack adaptive capabilities when confronted with uncertainty in object location or geometry.

##### Force control and tactile feedback

Tactile and force sensing are essential for handling delicate or poorly constrained objects. Sub-millimetre alignment in well-plate insertion has been demonstrated using integrated force/torque sensors and GelSight tactile sensors^19^ (Fig. 4B). Tactile, visual, and force feedback have also been combined to dynamically correct vial insertions during misalignment^17^. Torque-controlled scraping informed by force sensing has been used to maintain contact under changing tool and surface conditions^17^. These studies show how feedback enables robots to adjust in real time, improving resilience and reducing task failure rates. Nonetheless, many platforms still lack tactile sensing altogether, depending exclusively on vision, which limits robustness during contact-rich tasks.

##### Grasp variety and end-effector design

The design of the gripper or tool directly affects what the robot can manipulate. Some systems employ specialised end-effectors for specific tasks, such as customised spatulas for powder transfer^39^(Fig. 4C) or grippers with internal contours to hold labware securely^93^ (Fig. 4D). Others aim for greater versatility.

Grippers capable of handling multiple vial formats have been developed, integrating visual feedback to guide grasping^16,94^. However, generalisation remains limited. A syringe-specific gripper has been used to perform task-specific operations^81^, while many systems continue to rely on lab-specific tools. Few existing grippers are adaptable enough to handle the variety of shapes and materials encountered in chemistry laboratories.

Achieving and outperforming human-like dexterity remains a significant challenge due to the complexity of manipulating objects with varying shapes, sizes and material properties. Developing grippers that can adapt to different object geometries, along with control algorithms that manage nuanced force application and tactile feedback, is a continuing area of research. Digital twins enable virtual commissioning of end-effectors and contact-rich skills, allowing teams to tune grasp strategies, force limits and compliance before physical trials; evidence from reconfigurable manufacturing and robotic machining shows that twin-driven commissioning reduces integration errors and improves first-time-right execution of contact tasks, which directly supports laboratory manipulation under tight tolerances^26,27,95^. For chemical laboratories, where physical interaction often underpins workflow success, improvements in dexterity will be central to expanding the role of autonomous systems.

#### Perception

Perception is fundamental to robotic autonomy, underpinning the ability to interpret surroundings, track progress, and act with precision^79,96^. In laboratory environments filled with fragile instruments, varied materials, and dynamic procedures, robots must detect and localise objects, monitor experimental states, and respond to environmental variation^97^. These capabilities are critical not only for safe manipulation but also for linking physical actions to analytical feedback in autonomous experimental platforms. Formal definitions and cut-offs for Perception are set out in SM2.3, with corresponding study-by-study scores listed in SM3.2^4,87^.

##### Sensor variety

**Fig. 5:**
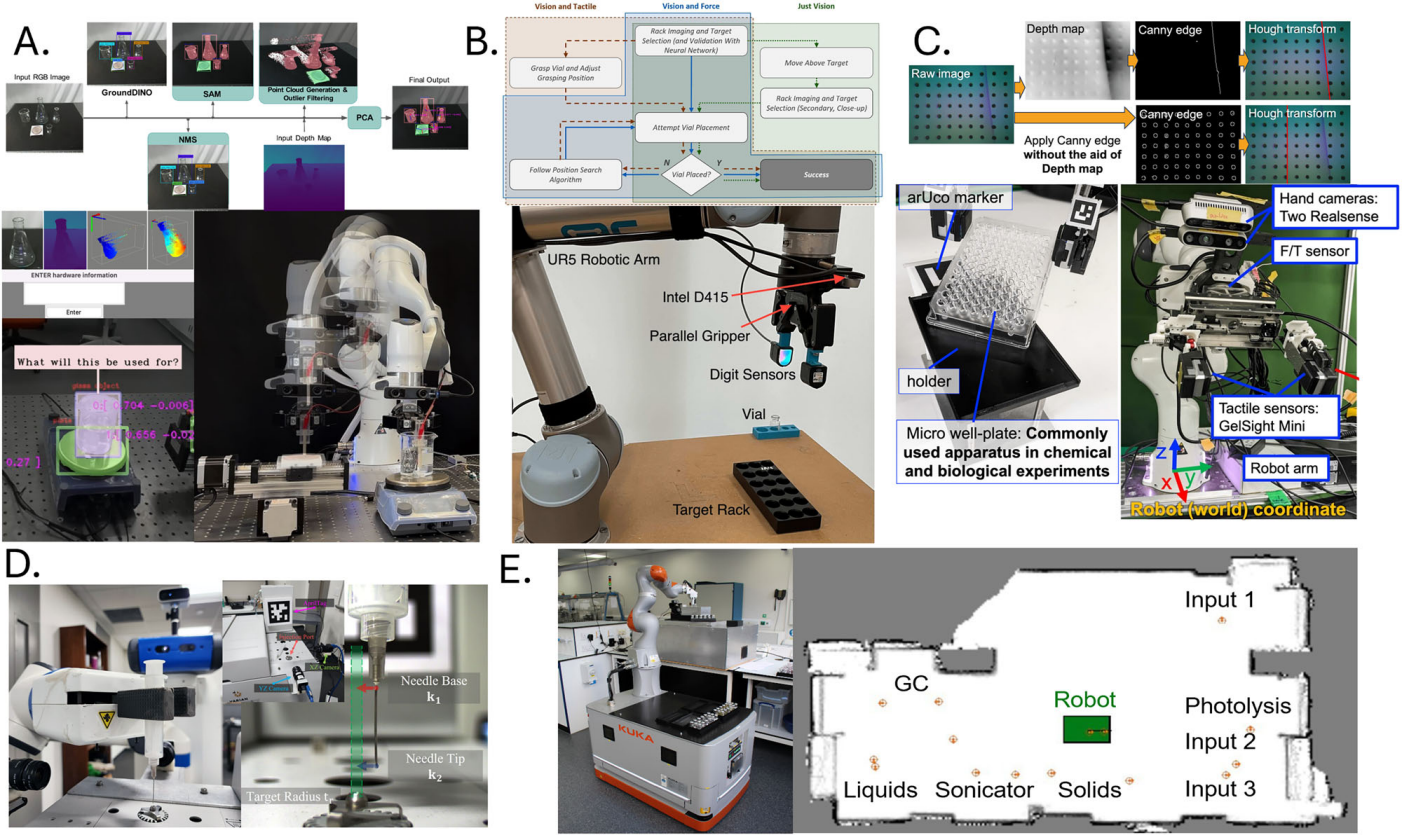
Robotic perception systems for laboratory autonomy. **A** ORGANA robotic assistant integrating advanced 3D visual perception for object detection, pose estimation and manipulation of transparent labware. **B** Multi-modal perception for vial insertion, combining visual, force and tactile sensing. **C** Integrated camera, force/torque and GelSight tactile sensors enabling precise well-plate insertion. **D** Multi-camera visual servoing for accurate syringe alignment during automated injection. **E** Mobile robotic chemist integrating laser scanning and touch sensing for autonomous navigation and experiment execution. **A** reproduced with permission from Darvish et al. (2024), © Elsevier. **B** reproduced with permission from the author (Butterworth et al., 2023). **C** reproduced from an arXiv preprint under a Creative Commons Attribution 4.0 (CC BY 4.0) licence (Pai et al., 2023). **D** reproduced with permission from the authors (Angelopoulos et al., 2023). Panel (E) reproduced with permission from Burger et al. (2020), © Springer Nature.

Recent developments have expanded the scope of robotic perception to encompass both environmental interpretation and experimental monitoring. A robotic assistant equipped with transformer-based 3D vision has been introduced for detecting and estimating the pose of transparent laboratory glassware^16^ (Fig. 5A). In addition to visual object recognition, this system integrates process-level sensors, including an analytical balance, pH probe, and potentiostat, to track key state variables such as mass, acidity, and electrochemical potential. This combination of object and process sensing enables closed-loop workflows such as solubility screening and electrochemistry.

A multi-camera visual servoing pipeline has been used to guide syringe alignment with sub-millimetre precision^81^ (Fig. 5D). High-precision well-plate insertion has been achieved using RGB vision, force and torque sensing, and GelSight tactile sensors (Fig. 5C), with real-time adjustment based on contact feedback^19^. Visual, tactile, and force sensors have also been combined to increase reliability in vial handling^17^ (Fig. 5B). An auditory-visual approach has been applied for monitoring powder grinding, using ultrasonic microphones and cameras to track material behaviour^98,99^. These examples underscore the increasing reliance on multi-modal sensing to enhance robustness and task precision. However, systems used in chemical laboratories must frequently manipulate transparent vessels, detect subtle visual indicators such as turbidity, and interface with analytical instrumentation. Despite progress, these remain challenging due to the variability of labware, signal ambiguity, and sensitivity to environmental conditions.

##### Feedback integration

Perception must not only gather information but also support dynamic decision-making during task execution. Simultaneous localisation and mapping (SLAM) with LIDAR, depth cameras, and force sensors has been used to enable robots to adapt navigation and manipulation strategies in real time^38,94^. These systems exemplify how feedback can refine movement and enable resilience to minor disturbances.

Integrating vision with tactile feedback has been shown to improve success rates in object placement^17^. Tactile contact data has also been used to guide sub-millimetre positioning during delicate insertions^19^. Such integrations allow robots to adjust grip and trajectory during execution. Yet, most systems still rely on case-specific tuning and lack the capacity to generalise to untrained task configurations. This is particularly limiting in chemical workflows where consistent sealing, pipetting, or reactor interfacing is essential to ensure reproducibility and experimental continuity.

##### Environmental awareness

Mobile robotic systems highlight the growing importance of spatial perception and contextual awareness. A mobile chemist has been developed that navigates using laser scanners and calibrates its end-effector position at each station through tactile contact with a known reference cube^76,93^ (Fig. 5E). This approach enabled accurate object handling despite station-to-station variability. Simultaneous localisation and mapping (SLAM) has also been implemented to coordinate the movement of multiple robots across a shared environment^100^. While these advances demonstrate effective navigation, they typically do not extend perception to ambient conditions such as temperature or humidity, which are often crucial in chemical engineering applications.

Powder weighing procedures have been guided using only balance readings, operating in fixed environments without contextual perception of workspace or object geometry^36^. Many systems continue to operate with limited adaptability, relying on fixed paths and assumptions of environmental stability^89^. This restricts deployment in open-ended or modular lab settings. Such limitations are particularly consequential in chemistry, where reaction success and safety often depend on detecting subtle changes in workspace configuration, reagent condition, or atmospheric control.

Transparent surfaces, occlusion, object similarity, and visual clutter continue to pose persistent challenges for perception systems^18,101,102^. Progress will depend on improved sensing and calibration, richer datasets and systematic stress testing; in this context, digital-twin resources can contribute by supplying labelled, physics-aware scenarios for hard-to-reproduce edge cases such as transparent vessels, glare and occlusion, with recent studies showing realistic sensor simulation and connected laboratory twins that support calibration, fusion testing and what-if analyses before deployment^25,95^. Addressing these limitations is critical for scaling autonomy beyond highly engineered setups and into flexible, multipurpose chemical laboratories.

#### Task complexity

Handling complex, multi-step procedures is a defining challenge for autonomous laboratory robots^77,103^. In chemical research, protocols often involve precise sequencing, conditional decision-making, and dynamic adaptation to intermediate outcomes^4,53,76^. Managing this complexity reliably and flexibly is essential for robotic systems to operate beyond structured automation and contribute meaningfully to scientific experimentation^15,104^. The Task Complexity scoring scheme is given in SM2.4; detailed evaluations for the cases discussed here are in SM3.2.

##### Multi-step workflows and automation

**Fig. 6:**
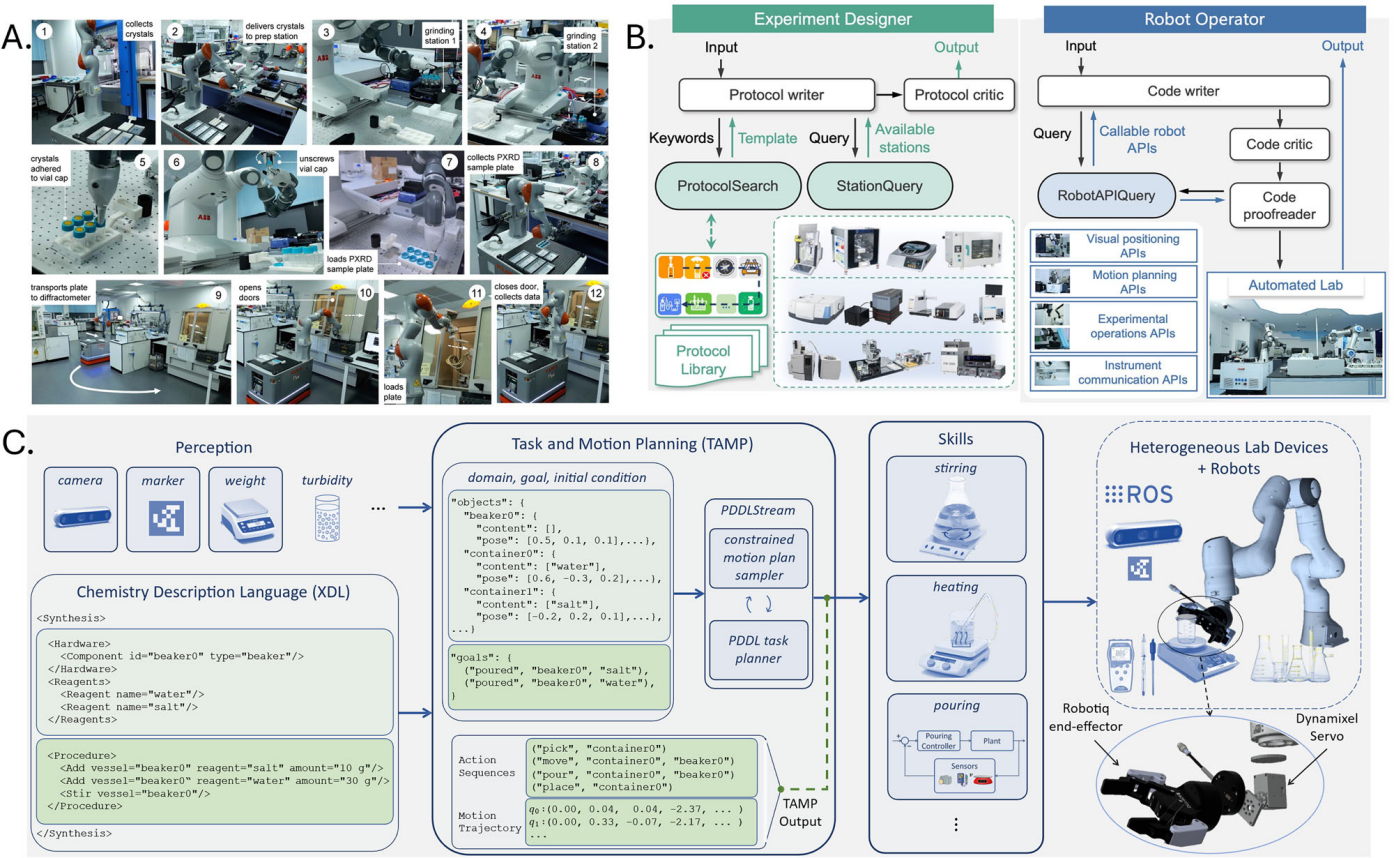
Robotic systems for complex lab tasks. **A** Fully autonomous powder X-ray diffraction (PXRD) workflow integrating sample preparation, handling and data collection across multiple robotic stations. **B** Multi-agent framework combining experiment design and robot operation to coordinate automated planning and task refinement across a distributed laboratory infrastructure. **C** Task-and-motion planning (TAMP) framework enabling adaptable laboratory actions, such as stirring, heating and pouring through integrated perception, planning and execution. Panel **A** reproduced from an arXiv preprint under a Creative Commons Attribution 4.0 (CC BY 4.0) licence (Lunt et al., 2024). Panel **B** reproduced with permission from Song et al. (2025), © American Chemical Society. Panel **C** reproduced from an arXiv preprint under a Creative Commons Attribution 4.0 (CC BY 4.0) licence (Yoshikawa et al., 2022).

Recent platforms have demonstrated that robots can now execute entire experimental procedures spanning multiple operations. A system has been developed to coordinate several robots in carrying out a 12-step powder X-ray diffraction (PXRD) workflow, encompassing sample preparation, transport, and data acquisition^100^(Fig. 6A). This capability has been further extended through the integration of a mobile manipulator and a rail-mounted arm to orchestrate experimental execution across 20 modular stations, including reaction, analysis, and cleaning steps^38^ (Fig. 6B). These platforms illucostrate the feasibility of autonomous protocol execution in realistic chemical workflows. However, most such systems depend on rigidly defined processes and require human intervention to update task logic or recover from failure.

##### Task and motion planning

Executing complex tasks requires generating action sequences that respect physical and temporal constraints. A task and motion planning (TAMP) framework using PDDLStream has been implemented to automate workflows involving object transport and pouring while avoiding collisions and spillage^105^ (Fig. 6C). By incorporating real-time perception feedback, the system adjusted its plans dynamically in response to environmental changes. These planning frameworks are a key component of complex laboratory automation but remain limited in generalisability. Custom planners are often task-specific and require significant reconfiguration when applied to different setups.

##### Handling environmental uncertainty

Real-world laboratories introduce uncertainty through variability in sample positioning, hardware availability, or experimental conditions. Visual and auditory feedback have been used to guide powder grinding and weighing tasks^36,98^. Tactile feedback has also been applied to correct positional errors in real time^17,19^. While these strategies improve resilience, many robotic systems still operate best in controlled settings. Effective task execution has been demonstrated in stable environments, but with limited responsiveness to unanticipated changes^81,93^. The ongoing challenge of enabling robots to adapt autonomously in unstructured or variable conditions remains a central focus of current research^89^.

##### Task variability and generalisation

The ability to generalise between tasks without extensive reprogramming remains limited. LLM-integrated systems have begun to show flexible task interpretation through natural language^16,38^. Adaptability across container types has been demonstrated using visual feedback, but most systems still require considerable manual intervention to switch contexts.

As task complexity increases, so does the computational demand for planning and control. Ensuring that robots can make timely decisions and adjustments requires efficient algorithms and processing capabilities. The integration of multiple sensory inputs and control strategies must be balanced with the need for real-time operation. System-level digital twins encode workflows, resources and constraints across instruments and stations, enabling pre-deployment validation, schedule optimisation and risk-aware replanning for long procedures; connected laboratory twins grounded in common data models have been proposed to enhance reproducibility and to coordinate end-to-end experimental processes from unit operations to integrated campaigns^24,25^. Additionally, verifying the correctness of autonomous decisions in safety-critical laboratory environments is a significant concern. Many systems still rely on predefined sequences and lack advanced learning methods for adaptability^92^.

## Outlook: the future of autonomous laboratory robotics

**Fig. 7:**
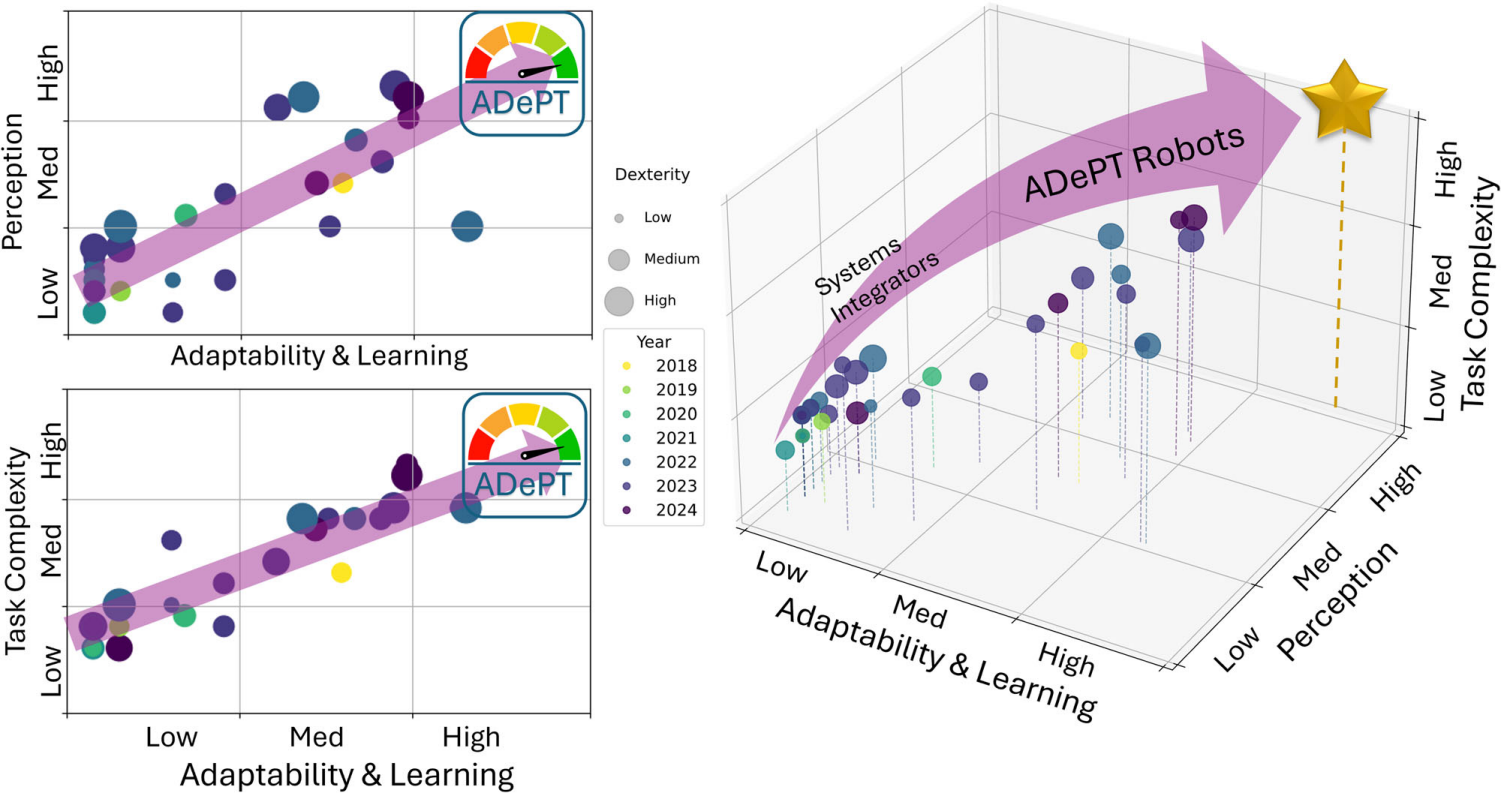
Evolution of laboratory robotics from Systems Integrators to ADePT Robots. . Each point represents a reported laboratory robotics study, with marker size indicating dexterity and colour denoting publication year. The observed trend reflects increasing integration of perception, learning and adaptability, marking a transition from fixed-function systems towards more autonomous, decision-enabled laboratory robots. Data are derived from ADePT evaluations (Supplementary Methods Sections SM3.1 and SM3.2), with aggregated scores reported in Supplementary Methods Section SM4.

The current landscape of laboratory robotics is diverse, with systems at varying levels of autonomy and functional sophistication. These advancements can be evaluated through the performance dimensions defined by the ADePT framework, which stands for Adaptability and Learning, Dexterity, Perception, and Task Complexity. This framework provides a structured basis for assessing the proficiency of robotic platforms as they evolve from simple automation tools into autonomous agents capable of dynamic decision-making. Figure 7 reflects this evaluation by positioning recent works along these dimensions, with each study assessed for how effectively it incorporates the core elements of autonomy (Supplementary Material, section SM3).

The ADePT framework also provides a lens through which to examine three major trajectories for the future of laboratory robotics. Each trajectory depends on different balances of ADePT capabilities. Robot-centric laboratories require high dexterity, advanced task execution, and minimal reliance on human input. End-to-end robotic integration prioritises adaptability and continuity across research and manufacturing domains. In collaborative human–robot settings, perception and real-time responsiveness are essential for safe and effective interaction. These paths highlight distinct models for future laboratories, each supported by a growing set of enabling technologies.

Most existing systems function as systems integrators, primarily managing physical tasks such as the transfer of labware and reagents between instruments within controlled workflows. Robots operating in this capacity excel at high-precision, repetitive operations. They effectively free human researchers from routine handling tasks but rely on pre-programmed instructions. Examples include earlier systems that remain concentrated in the lower range of adaptability and perception^59,100^. These systems demonstrate limited ability to respond dynamically to changes in their environment, focusing instead on consistent task execution within predefined workflows. This positioning highlights both their value in automation and their limitations in achieving higher levels of autonomy.

Recent research illustrates a clear trend toward systems with greater perception and adaptability. This shift is reflected in the figure by a progression toward higher performance along the ADePT dimensions. Systems that integrate multi-modal perception and advanced planning capabilities are enabling more flexible and independent robotic behaviour in laboratory workflows. These developments point toward platforms that can interpret complex instructions, adjust to experimental variability, and operate with increasing autonomy based on contextual awareness. Each point in Fig. 7 corresponds to one peer-reviewed study scored under ADePT; the underlying per-study evaluations are in SM3.1 (systems integrators) and SM3.2 (key features), with the full score matrix in SM4. The transition from systems integrators to more autonomous robots suggests a movement toward platforms that are no longer confined to static protocols.

As progress in these key areas continues, the vision of a fully autonomous laboratory is becoming more realistic. Robots will not only perform routine tasks but also make independent decisions, adapt to new experimental designs, and collaborate effectively with human researchers. This perspective highlights the technological and conceptual advancements that are likely to shape the laboratories of the future and underscores the role of the ADePT framework in guiding their development.

**Fig. 8:**
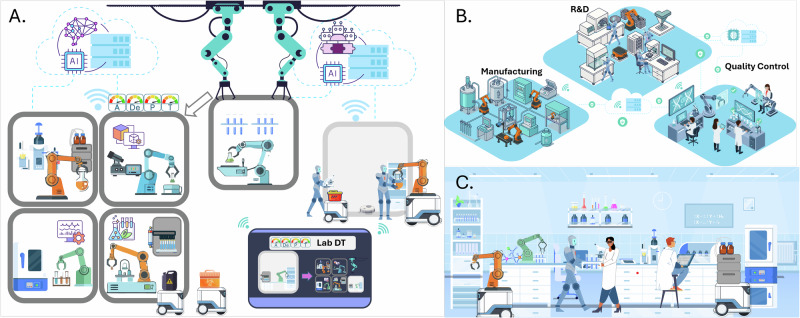
Next-generation robot-centric laboratory paradigms. **A** Conceptual unmanned, robot-centric laboratory in which experimental operations, including setup, execution, analysis, cleaning and waste handling are autonomously orchestrated by robotic systems within modular and reconfigurable facilities, supported by a laboratory digital twin (Lab DT). **B** End-to-end robotic integration spanning research, development, manufacturing and quality control, enabling continuous data and material flow across the laboratory lifecycle. **C** Collaborative human–robot laboratory with safe, intuitive shared workspaces enabled by multimodal perception; robots perform repetitive, hazardous or precision-critical tasks while humans guide experimental design, interpretation and decision-making.

The capabilities defined by the ADePT framework are not only essential for evaluating the current state of laboratory robotics but also serve as a foundation for understanding how autonomous systems will shape the laboratories of the future (Fig. 8). Each of the four dimensions plays a distinct role in supporting different operational models. Autonomous laboratories are intended to augment scientific judgement rather than replace it. Framed in ADePT terms, robots offset human limitations in Dexterity when contact-rich precision is required over long durations or in hazardous settings, extend Perception through consistent multi-sensor monitoring, increase Adaptability and Learning by updating policies as conditions drift, and expand Task Complexity by scheduling and executing long multi-step protocols without fatigue. Human strengths remain central for problem framing, hypothesis revision and risk acceptance, and studies of self-driving laboratories and human-in-the-loop decision-making and supervision show that hybrid workflows deliver faster convergence and improved robustness compared to unattended operation^28,29,61,106^.

The following perspectives explore three emerging configurations of laboratory automation, each placing different demands on ADePT capabilities. Robot-centric laboratories require advanced dexterity and autonomous task management. End-to-end integration depends on high adaptability and continuity across experimental and industrial processes. Collaborative environments prioritise safe perception, context awareness, and flexible planning. These visions reflect complementary paths toward more intelligent, connected, and autonomous laboratory ecosystems. The component scores reported in SM3.1–SM3.2 and summarised in SM4 underpin the trends discussed here and in Fig. 7.

Across all three trajectories, credibility and uptake depend on reproducibility, safety, sustainability and ethics, which we treat as cross-cutting requirements for ADePT-capable systems.

### Reproducibility and reporting

For credibility and reuse, autonomous laboratory studies should report quantitative error and robustness alongside ADePT scores. At a minimum, these should include per-step error rates, inter- and intra-run variance, failure modes with recovery outcomes, and complete data and protocol provenance that follow FAIR principles and enable replication in other labs^107,108^. Recent perspectives on self-driving laboratories emphasise transparent metadata, dataset curation and workflow audit trails as enablers of reproducible closed-loop experimentation in chemistry and materials^4,61^.

### Safety and ethics in unmanned and shared spaces

Operating unattended or in human–robot co-presence requires formal safety cases and documented limits. Evidence from human–robot collaboration research supports risk assessment, speed and separation monitoring, contact-force limits, and validation of stop functions, with compliant control reducing residual risk in shared workspaces^88,109^. Ethical governance for autonomous decision making should include accountabilities for human override, data governance for human-in-the-loop feedback, and documentation of model limits and failure modes, aligning with contemporary analyses of responsible AI in scientific contexts^110–112^.

### Sustainability and environmental performance

Robotic laboratories can consolidate workflows and enable smart scheduling, but should quantify environmental performance to avoid shifting burdens. Practical metrics include energy per experiment, solvent and single-use plastic mass per result, emission-based metrics (e.g., global warming potential), green chemistry metrics (e.g., E-factor and process mass intensity), and life cycle assessment^4,113,114^. Reporting these indicators alongside ADePT component scores provides a unified view of capability, quality and impact that supports continuous improvement.

### Next generation Robot-centric Labs

One vision for the future is the unmanned, robot-centric laboratory (Fig. 8A). In this configuration, all tasks such as experiment setup, raw material handling, execution, sampling and analysis, cleaning, and waste management are orchestrated by robotic systems. The next-generation lab facilities may feature compact, stacked modules to maximise spatial efficiency, and nonstandard architecture such as vertical labs. Reconfigurable stations and plug-and-play instruments, similar to those proposed in recent modular synthesis frameworks, will support flexible experimental workflows without human intervention^51,53^. Advanced orchestration systems and connected instruments will consolidate the unmanned lab ecosystem and ensure operational excellence.

Beyond reducing manual set-up burden, unmanned operation improves utilisation of shared instruments through automated scheduling, orchestration and resource management, as shown by connected laboratory twin platforms and digital facility management frameworks^25,115^. Digital twins will enable virtual commissioning of the unmanned lab virtually by verifying layouts, reachability, collision margins, safety interlocks and recovery logic before any hardware is touched. Evidence from twin-driven virtual commissioning indicates fewer integration iterations and faster start-up for contact-rich tasks, which translates directly to quicker turnover of autonomous experimental campaigns^26,27^.

As robots take full control of lab operations, laboratory equipment will be fully or partly redesigned to accommodate robot features more effectively. This includes not only the development of new robotic tools but also the adaptation of existing instruments and process analytical technologies to support robotic access and interaction. For example, the design of the Crystal LINE system has incorporated simplified interfaces to enable robotic vial insertion, reflecting a broader trend toward improving the compatibility of analytical platforms with autonomous handling systems. Such adjustments will be essential for achieving seamless operation across diverse robotic workflows.

Current standards and regulatory frameworks associated with workplace exposure limits, such as control of substances hazardous to health (COSHH) and noise-related limits, may be revised to account for the ability of robots to function in harsher environments and under extended exposure conditions. The use of personal protective equipment may be significantly reduced or supplanted. Robots will support seamless orchestration of laboratory tasks, reducing the risk of cross-contamination, particularly in bio- and pharmaceutical settings. These advances may help to elevate standards of good laboratory practice (GLP) and good manufacturing practice (GMP), ultimately contributing to safer and more consistent pharmaceutical development.

Advances in artificial intelligence are critical to enabling this level of autonomy. Large language models (LLMs) and symbolic reasoning systems have already demonstrated the ability to interpret experimental objectives and generate protocols^16,38,116^. These capabilities allow robots to function as planning agents as well as executors. As systems mature, they will dynamically optimise experimental parameters, adapt to data trends, and reconfigure workflows without human instruction. In addition, Digital twins can commission the unmanned lab virtually by verifying layouts, reachability, collision margins, safety interlocks and recovery logic before any hardware is touched. Evidence from twin-driven virtual commissioning indicates fewer integration iterations and faster start-up for contact-rich tasks, which translates directly to quicker turnover of autonomous experimental campaigns^26,27^.

Autonomous decision-making for an unmanned lab can be realised with a layered architecture that couples multi-robot scheduling, task-and-motion planning, and robust motion generation. At the top layer, constraint-programming schedulers allocate jobs across robots and stations while resolving shared resources and routes in parallel experimental campaigns, as recently demonstrated for autonomous chemistry laboratories coordinating multiple robots and 18 stations^117^. Where centralised plans need online adaptation, cooperative multi-agent reinforcement learning with explicit inter-agent communication provides decentralised conflict resolution and re-planning under uncertainty^118^. At execution time, integrated task-and-motion planning bridges symbolic procedures to feasible motions in cluttered lab scenes, with optimisation-based TAMP now surveyed comprehensively and with laboratory examples in rack loading and similar manipulation^119,120^. Low-level motion can rely on sampling-based planners that scale to high-DOF manipulators and tight clearances, offering a practical foundation for collision-free operation on crowded benches^121^. Together, these elements constitute a feasible decision-making stack for unattended operation and map directly to ADePT by raising task complexity and adaptability while protecting Dexterity constraints.

In such settings, robots will not only match but in some domains exceed human dexterity, using integrated tactile sensing, compliant actuation, and precision control to manipulate diverse labware with sub-millimetre accuracy. Coupled with robust perception systems capable of detecting subtle environmental changes, they will operate continuously with minimal failure, navigating complex workspaces and monitoring process quality in real time.

Realising this vision will require more than algorithmic improvements. Robustness and resilience are key. Robotic systems must tolerate variation in material properties, environmental conditions, and instrument performance. This will require advanced fault detection, error recovery, and predictive maintenance. While some platforms already detect failed insertions or force anomalies, many still require manual intervention for calibration and correction.

Sustainability will also play a critical role. Robot-centric laboratories offer opportunities to reduce energy and space usage by condensing workflows, automating lighting and ventilation, and minimising human-support infrastructure. However, the environmental footprint of continuous operation, sensor networks, and computational resources must be carefully managed. Metrics for lifecycle energy use and material consumption will be needed to assess and mitigate the environmental costs of full automation.

### End-to-end robotic integration

A second trajectory envisions not only autonomous experimentation but full integration of robotic systems across research, development, manufacturing, and quality control (Fig. 8B). In this model, data and materials flow seamlessly through robotic platforms, from hypothesis generation to commercial production, and real-time testing and release of products. Such end-to-end automation enables consistent documentation, real-time feedback loops, and continuous process improvement.

While robotic systems are already common in manufacturing, their extension into quality control remains underdeveloped. Laboratories for raw material and product testing and validation often remain human-operated, which introduces variability and longer lead times. Integrating robotics into these environments could reduce cycle times, improve standardisation, and support real-time release testing. This would be particularly valuable in regulated sectors such as pharmaceuticals, where quality control is critical.

End-to-end integration also opens new possibilities for experiment-to-manufacture translation. Experimental data can directly inform process optimisation, and robotic platforms can reproduce synthesis routes at different scales with minimal reconfiguration. This continuity reduces the risk of knowledge loss between R&D and production stages, improving efficiency and reproducibility across the product lifecycle. To sustain this continuity across sites, a connected fabric of digital twins can bridge R&D, QC and manufacturing by synchronising state, provenance and specifications; recent proposals show how laboratory twins grounded in shared ontologies support traceability and change management, and chemical-industry reviews detail implementation pathways for scalable twin deployment, including interoperability with OPC UA information models^24,25^.

AI-enabled robots will continuously refine their performance through learning from both simulation and real-world feedback, enabling real-time self-optimisation and on-the-fly adjustment of protocols and process parameters. Their ability to interpret sensor data, recognise anomalies, and autonomously update control strategies will underpin increasingly complex operations. As adaptability becomes more embedded in laboratory control systems, these robots will expand beyond static roles, becoming true agents of process innovation.

However, challenges remain. Interfacing different robotic platforms across the development chain requires common standards for communication, control, and data formats. Realising this vision will depend on collaborative efforts among equipment manufacturers, software developers, and regulatory bodies. Investment in interoperability and long-term support infrastructures will be essential for deployment at scale.

Beyond protocols, sustained adoption depends on shared ontologies and provenance. Laboratory digital-twin frameworks grounded in common data models demonstrate how to coordinate workflows, enforce change control, and preserve end-to-end traceability across R&D, QC and manufacturing, with clear pathways for integrating OPC UA information models^24,25^. Deterministic transport using time-sensitive networking has been reviewed as a viable path to information technology/operational technology (IT/OT) convergence from bench to plant, reducing timing uncertainty in cross-tool coordination^122^.

Building on these foundations, a practical route to end-to-end robot integration is to layer multi-agent orchestration on top of the standardised interfaces and digital twins already in place. Station, or instrument-level agents negotiate task allocation and timing, while a plant-level coordinator resolves shared resources and deadlines. Recent studies demonstrate multi-robot, multi-task scheduling across manipulators and stations in autonomous chemistry, and surveys of multi-agent decision making outline auction- and market-based policies that remain robust under uncertainty and streaming jobs^117,123,124^. Agentic pipelines in biomedicine further show how specialist agents can coordinate planning, perception and execution across complex workflows^125^.

In ADePT terms, this stack raises Task Complexity by scaling interlinked workflows across sites, strengthens Adaptability and Learning through online reallocation and replanning, and preserves Dexterity by honouring device-level constraints exposed via information models. Deployment risks are addressed with audit trails and human override at defined decision points, phased adoption that targets OPC UA-modelled bottleneck instruments to control cost, and governance that documents model limits and failure modes in line with responsible-AI practice^110,111^

### Collaborative human–robot labs

Despite the promise of full autonomy, there is also a compelling case for laboratories where humans and robots collaborate (Fig. 8C). In this scenario, robots take on repetitive, hazardous, or precision-dependent tasks, while humans focus on conceptual design, interpretation, and innovation. Such arrangements are particularly suited to exploratory research, where task flexibility and on-the-spot decision-making remain valuable.

Empirical examples underline when collaboration outperforms full autonomy. Human-guided Bayesian and model-based experimental campaigns achieve higher sample efficiency in exploratory regimes where objectives and constraints evolve during the run^28,29^. In wet-lab co-working, vision-plus-force pipelines enable safe insertion, handovers and recovery behaviours at cycle times that would be unsafe or impractical for manual repetition, while learning from demonstration shortens the path from intent to executable skills^126–128^. Physical human–robot interaction studies further show that role allocation and compliant control reduce residual risk in shared spaces, supporting routine co-presence in bench-scale laboratory tasks^129^.

Practically, collaboration is operationalised by checkpointed planners that request human approvals at pre-defined decision nodes, safety envelopes validated against contact-force limits, and transparent reporting of error and recovery events. In materials and chemical campaigns, human feedback has been incorporated as priors or constraints within active learning and Bayesian optimisation loops, yielding safer, more informative experiments with fewer iterations^28,61^. Reporting these design choices alongside ADePT component scores clarifies how people and robots share responsibility and where capability upgrades are most beneficial.

Safe, intuitive interaction is central to this approach. Robots will increasingly use multimodal perception systems, including vision, force sensing, and proximity detection, to operate safely alongside humans. The feasibility of shared workspaces and real-time adjustment has been demonstrated in recent systems^17,19^. Future systems will combine these capabilities with predictive planning and human-intent recognition, enabling coordinated workflows where human and robot actions are dynamically aligned. In human–robot settings, digital twins offer a safe sandbox for training and evaluation of shared-autonomy policies, intent inference and workspace zoning, and they provide a living record for continuous improvement of interaction. Reviews report that twin platforms can mediate perception and planning with human feedback and support operator training and scenario rehearsal for safer co-working^130,131^.

Key challenges include ethical oversight, cost and maintainability, operator workload and trust, and transparent reporting of errors and recoveries^25,132,133^. Countermeasures are to retain human override at defined decision points, phase deployments where the cost–benefit is clearest, and publish per-study logs of safety incidents, recovery metrics and reproducibility rates^134–137^. In ADePT terms, these practices raise Adaptability and Learning via human-guided updates, sustain Perception through operator-in-the-loop validation of ambiguous scenes, and increase Task Complexity by enabling mixed-initiative workflows without degrading safety or throughput. Guidance from responsible and transparent AI on accountability and documentation complements laboratory safety reporting, while recent human–algorithm collaboration studies show improved performance and sample-efficiency when expert priors are integrated explicitly^28,29,110,111,138^.

Lab infrastructure will evolve to support this interaction. Spaces may be zoned for human and robotic activity or feature tools designed for dual use. Adaptive planning algorithms will ensure robots can adjust their strategies in response to changing workflows or unexpected intervention. This hybrid model also improves resilience. In the event of hardware failure or unexpected conditions, human operators can intervene. Conversely, robots provide continuity for routine operations, reducing bottlenecks and enabling researchers to focus on higher-level problem-solving.

## Conclusion

Laboratory robotics is undergoing a profound transformation, progressing from isolated automation modules toward systems capable of intelligent and adaptive operation across complex workflows. This shift has been driven by advances in perception, learning, manipulation, and planning, which together form the foundation of laboratory autonomy^77,139^. While many current systems remain limited to structured tasks within defined settings, the field is steadily advancing toward platforms that function reliably in dynamic and data-rich environments^4^.

In chemical research and development, robots are no longer confined to automating repetitive procedures. They are now contributing meaningfully to experimental design, execution, and optimisation. These systems can interpret sensor feedback, make informed adjustments, and coordinate activity across multiple instruments and stages of experimentation^55,58,93^. Artificial intelligence and machine learning further enhance this progression by enabling robots to explore parameter spaces, respond to variability, and adapt in real time^140,141^.

This Perspective introduces a conceptual benchmark that captures the core proficiencies required for autonomous operation in laboratory environments. We define these capabilities using the ADePT framework, which stands for Adaptability and Learning, Dexterity, Perception, and Task Complexity. The term ADePT evokes skillfulness and proficiency, reflecting the evolving role of robots in scientific discovery. These four dimensions serve as a foundation for evaluating the development and deployment of laboratory robotics and provide a structured approach for aligning robotic capabilities with the needs of self-driving laboratories^85,88,103^. We expect that ADePT-capable systems will be essential to the future of laboratory automation, supporting the advancement of robot-exclusive laboratories, end-to-end robotic integration across research and manufacturing, and collaborative human–robot environments.

Several challenges remain before these visions can be realised. Laboratory settings are complex and unpredictable, demanding robustness and adaptability that current systems do not consistently achieve. Many robotic platforms still struggle with fine motor control, safe handling of delicate labware, and autonomous recovery from unexpected conditions^121,129^. Planning systems capable of adjusting workflows in real time are under active development but are not yet broadly reliable in unsupervised operation^103^.

Despite these limitations, progress is accelerating. Modular hardware, reconfigurable laboratory layouts, and improved sensor systems are making robotics more versatile and easier to integrate^51,142^. Robots are becoming more self-sufficient, not only executing predefined protocols but also generating, refining, and validating experimental workflows in response to live data. Advances in fault detection and error recovery are contributing to increased resilience, reducing the need for human oversight and enabling longer operational cycles.

Looking ahead, multiple trajectories are likely to shape the laboratory of the future. Some laboratories will become entirely robot-centric, designed for high-throughput operation and continuous activity. Others will foster hybrid environments where robots work alongside researchers, providing support for repetitive, hazardous, or precision-demanding tasks. In both cases, robotics will contribute to safer, more efficient, and more sustainable laboratory operations. The widespread adoption of autonomous platforms will also influence regulatory practices, quality control, energy use, and the physical design of laboratory infrastructure.

Looking ahead, multiple trajectories are likely to shape the laboratory of the future. Some laboratories will become entirely robot-centric, designed for high-throughput operation and continuous activity, while others will foster hybrid environments where robots work alongside researchers on repetitive, hazardous, or precision-demanding tasks. In both cases, robotics will contribute to safer, more efficient, and more sustainable operations, with digital twins increasingly providing the connective tissue for commissioning, monitoring, and end-to-end traceability across research and development, quality control, and manufacturing^24,25^. Collaborative human-in-the-loop workflows will remain important for safe adaptation in exploratory regimes, complementing autonomous decision-making^28,29^. The widespread adoption of autonomous platforms will also influence regulatory practices, quality control, energy use, and the physical design of laboratory infrastructure, and will benefit from systematic reporting of error, recovery, and reproducibility metrics to ensure accountability and sustained performance at scale.

The objective is not to mimic human scientists with humanoid machines, but rather to extend the capabilities of the laboratory itself. By enabling precise, adaptive, and seamless workflows, robotics can expand the scale and scope of experimentation. Achieving this will require the integration of automation technologies with digital systems, the redesign of laboratory spaces and tools, and the development of regulatory frameworks that reflect the capabilities and requirements of autonomous platforms. Real-world impact will hinge not only on ADePT-grade capabilities but also on rigorous standardisation, including open command schemas, shareable information models and auditable data flows, to enable modularity, knowledge sharing and sustained adoption^22,23,62^. The ADePT framework provides a useful guidepost for evaluating and advancing these efforts as laboratory science enters a new era.

## Supplementary information

Supplementary material pdf 26-01-26

## References

[1] Abolhasani, M. & Kumacheva, E. The rise of self-driving labs in chemical and materials sciences. *Nat. Synth.* **2**, 483–492 (2023).

[2] Xie, Y., Sattari, K., Zhang, C. & Lin, J. Toward autonomous laboratories: convergence of artificial intelligence and experimental automation. *Prog. Mater. Sci.* **132**, 101043 (2023).

[3] Canty, R. B. et al. Science acceleration and accessibility with self-driving labs. *Nat. Commun.* **16**, 3856 (2025). 40274856 10.1038/s41467-025-59231-1 PMC12022019

[4] Tom, G. et al. Self-driving laboratories for chemistry and materials science. *Chem. Rev.* **124**, 9633–9732 (2024). 39137296 10.1021/acs.chemrev.4c00055 PMC11363023

[5] Maffettone, M. P. et al. What is missing in autonomous discovery: open challenges for the community. *Digit. Discov.* **2**, 1644–1659 (2023).

[6] Christensen, M. et al. Automation isn’t automatic. *Chem. Sci.* **12**, 15473–15490 (2021). 35003576 10.1039/d1sc04588a PMC8654080

[7] Zwirnmann, H., Knobbe, D., Culha, U. & Haddadin, S. Towards flexible biolaboratory automation: container taxonomy-based, 3D-Printed Gripper Fingers*. In *IEEE International Conference on Intelligent Robots and Systems*, (ed. Gregg, B.) 6823–6830 (2023).

[8] Canty, R. B., Koscher, B. A., McDonald, M. A. & Jensen, K. F. Integrating autonomy into automated research platforms. *Digit. Discov.* **2**, 1259–1268 (2023).

[9] Grønseth, B. O. & Madsen, D. Ø. Industry 4.0. In *Encyclopedia of Tourism Management and Marketing*, Vol. 2 (ed. Buhalis, D.) 683–685 (Edward Elgar Publishing, 2022).

[10] Ghobakhloo, M. Industry 4.0, digitization, and opportunities for sustainability. *J. Clean. Prod.* **252**, 119869 (2020).

[11] Xu, X., Lu, Y., Vogel-Heuser, B. & Wang, L. Industry 4.0 and Industry 5.0—inception, conception and perception. *J. Manuf. Syst.* **61**, 530–535 (2021).

[12] Leng, J. et al. Industry 5.0: prospect and retrospect. *J. Manuf. Syst.* **65**, 279–295 (2022).

[13] MacLeod, B. P., Parlane, F. G. L., Brown, A. K., Hein, J. E. & Berlinguette, C. P. Flexible automation accelerates materials discovery. *Nat. Mater.* **21**, 722–726 (2022). 34907322 10.1038/s41563-021-01156-3

[14] MacLeod, B. P., Parlane, F. G. L. & Berlinguette, C. P. How to build an effective self-driving laboratory. *MRS Bull.* **48**, 173–178 (2023).

[15] Bayley, O., Savino, E., Slattery, A. & Noël, T. Autonomous chemistry: Navigating self-driving labs in chemical and material sciences. *Matter* **7**, 2382–2398 (2024).

[16] Darvish, K. et al. ORGANA: a robotic assistant for automated chemistry experimentation and characterization. *Matter* **8**,101897 (2025). **Demonstrates an integrated robotic chemistry assistant combining 3D vision with process sensors to execute and monitor workflows, exemplifying how robust perception underpins reliable autonomy**.

[17] Butterworth, A., Pizzuto, G., Pecyna, L., Cooper, A. I. & Luo, S. Leveraging multi-modal sensing for robotic insertion tasks in R&D laboratories. In *2023 IEEE 19th International Conference on Automation Science and Engineering (CASE)*, (ed. Aw, K.), 1–8 (IEEE, 2023). **Shows how multi-modal sensing (vision/force/tactile) improves robustness in contact-rich insertion tasks, directly advancing dexterity and in-situ adaptation in lab manipulation**.

[18] Jiang, J., Cao, G., Butterworth, A., Do, T.-T. & Luo, S. Where shall I touch? Vision-guided tactile poking for transparent object grasping. *IEEE/ASME Trans. Mechatron.* **28**, 233–244 (2023).

[19] Pai, S. et al. Precise well-plate placing utilizing contact during sliding with tactile-based pose estimation for laboratory automation. In *2024 IEEE/RSJ International Conference on Intelligent Robots and Systems (IROS)* 5252–5259 (IEEE, 2024). **Introduces tactile-based pose estimation for sub-millimetre well-plate placement, providing a concrete route to high-precision, failure-resistant laboratory handling**.

[20] Christensen, M. et al. Data-science driven autonomous process optimization. *Commun. Chem.* **4**, 112 (2021). 36697524 10.1038/s42004-021-00550-x PMC9814253

[21] Knobbe, D., Zwirnmann, H., Eckhoff, M. & Haddadin, S. Core processes in intelligent robotic lab assistants: flexible liquid handling. In *2022 IEEE/RSJ International Conference on Intelligent Robots and Systems (IROS)*, (eds Wang, Z., Ando, N. & Yamanobe, N.) 2335–2342 (IEEE, 2022).

[22] Brendel, A. et al. Laboratory and Analytical Device Standard (LADS): a communication standard based on OPC UA for networked laboratories. In *Advances in Biochemical Engineering/Biotechnology*, (eds Beutel, S. & Lenk, F.) Vol. 182, 175–194 (2022). 10.1007/10_2022_209 35861885

[23] Juchli, D. SiLA 2: the next generation lab automation standard. In *Advances in Biochemical Engineering/Biotechnology* Vol. 182, 147–174 (Springer Science and Business Media Deutschland GmbH, 2022). 10.1007/10_2022_204 35639108

[24] Mane, S., Dhote, R. R., Sinha, A. & Thirumalaiswamy, R. Digital twin in the chemical industry: a review. *Digit. Twins Appl.* **1**, 118–130 (2024).

[25] Rihm, S. D. et al. Transforming research laboratories with connected digital twins. *Nexus*. **1**, 100004 (2024).

[26] Iyer, S. V. & Sangwan, K. S. & Dhiraj. Digital twin-based virtual commissioning for evaluation and validation of a reconfigurable process line. *IET Coll. Intell. Manuf.* **6**, e12111 (2024).

[27] Ni, H. et al. Digital twin-driven virtual commissioning for robotic machining enhanced by machine learning. *Robot Comput. Integr. Manuf.* **93**, 102908 (2025).

[28] Adams, F., McDannald, A., Takeuchi, I. & Kusne, A. G. Human-in-the-loop for Bayesian autonomous materials phase mapping. *Matter* **7**, 697–709 (2024). **Illustrates a practical autonomy pattern where Bayesian experimentation is safely accelerated** via **human-in-the-loop oversight, clarifying how decision logic and autonomy can be staged in real labs**.

[29] Biswas, A. et al. A dynamic Bayesian optimized active recommender system for curiosity-driven partially human-in-the-loop automated experiments. *NPJ Comput Mater.* **10**, 29 (2024).

[30] Yewale, A. et al. Deep reinforcement learning-based self-optimization of flow chemistry. *ACS Eng. Au* **5**, 247–266 (2025). **Demonstrates reinforcement-learning-driven self-optimisation in flow chemistry, highlighting how learning can reduce manual tuning and adapt control policies to process variability**. 40556644 10.1021/acsengineeringau.5c00004 PMC12183679

[31] Little, J. N. The Zymate laboratory automation systems. *J. Liq. Chromatogr.* **9**, 3197–3201 (1986).

[32] Frisbee, A. R., Nantz, M. H., Kramer, G. W. & Fuchs, P. L. Laboratory automation. 1: syntheses via vinyl sulfones. 14. Robotic orchestration of organic reactions: yield optimization via an automated system with operator specified reaction sequences. *J. Am. Chem. Soc.* **106**, 7143–7145 (1984).

[33] Bai, J. et al. From platform to knowledge graph: evolution of laboratory automation. *JACS Au* **2**, 292–309 (2022). 35252980 10.1021/jacsau.1c00438 PMC8889618

[34] Wolf, Á et al. Towards robotic laboratory automation Plug & Play: the “LAPP” framework. *SLAS Technol.* **27**, 18–25 (2022). 35058216 10.1016/j.slast.2021.11.003

[35] Rafael Vescovi et al. Towards a modular architecture for science factories. *Digit. Discov.* **2**, 1980–1998 (2023).

[36] Kadokawa, Y., Hamaya, M. & Tanaka, K. Learning robotic powder weighing from simulation for laboratory automation. In *2023 IEEE/RSJ International Conference on Intelligent Robots and Systems (IROS)*, (ed. Gregg, B.) 2932–2939 (IEEE, 2023). **Shows simulation-trained policies transferring to real robotic powder weighing, evidencing a scalable route to adaptable handling across materials and target masses**.

[37] Yoshikawa, N. et al. Large language models for chemistry robotics. *Auton. Robots* **47**, 1057–1086 (2023).

[38] Song, T. et al. A multiagent-driven robotic AI chemist enabling autonomous chemical research on demand. *J. Am. Chem. Soc.* **147**, 12534–12545 (2025). **Presents a multi-agent architecture that coordinates planning and execution across automated resources, exemplifying system-level task complexity management for autonomous lab operation**. 40056128 10.1021/jacs.4c17738

[39] Jiang, Y. et al. Autonomous biomimetic solid dispensing using a dual-arm robotic manipulator. *Digit. Discov.*10.1039/D3DD00075C (2023). **Demonstrates autonomous solid dispensing with dual-arm manipulation, addressing a core bottleneck in lab robotics where dexterous, repeatable handling is essential**.

[40] Yoshikawa, N. et al. Chemistry lab automation via constrained task and motion planning. https://acrad.github.io/robot-chemist-tamp/ (2022).

[41] Oldenburg, K. R. Automation basics: robotics vs. workstations. *J. Biomol. Screen* **4**, 53–56 (1999). 10838411 10.1177/108705719900400203

[42] Elands, J. The evolution of laboratory automation. In *Handbook of Drug Screening*, (eds Seethala, R. & Fernandes, P. B.) 498–513 (CRC Press, 2001).

[43] Gecks, W. & Pedersen, S. T. Robotics—an efficient tool for laboratory automation. *IEEE Trans. Ind. Appl.* **28**, 938–944 (1992).

[44] Ahmed, N. & Sowmya, A. AutoLab: a robotics solution for flexible laboratory automation. In *Intelligent Robots and Computer Vision XIII: 3D Vision, Product Inspection, and Active Vision* Vol. 2354 (ed. Casasent, D. P.) 205–214 (SPIE, 1994).

[45] Sparkes, A. et al. Towards robot scientists for autonomous scientific discovery. *Autom. Exp.* **2**, 1–11 (2010). 20119518 10.1186/1759-4499-2-1 PMC2813846

[46] King, R. D. et al. The automation of science. *Science (1979)* **324**, 85–89 (2009). 10.1126/science.1165620 19342587

[47] Williams, K. et al. Cheaper faster drug development validated by the repositioning of drugs against neglected tropical diseases. *J. R. Soc. Interface* **12**, 20141289 (2015). 25652463 10.1098/rsif.2014.1289 PMC4345494

[48] King, R. D., Schuler Costa, V., Mellingwood, C. & Soldatova, L. N. Automating sciences: philosophical and social dimensions. *IEEE Technol. Soc. Mag.* **37**, 40–46 (2018).

[49] MacLeod, B. P. et al. Self-driving laboratory for accelerated discovery of thin-film materials. *Sci. Adv.* **6**, eaaz8867 (2020). **A landmark self-driving lab that closes the loop between models and experiments for thin-film discovery, illustrating how autonomy can accelerate materials optimisation campaigns**. 10.1126/sciadv.aaz8867 PMC7220369 32426501

[50] Coley, C. W. et al. A robotic platform for flow synthesis of organic compounds informed by AI planning. *Science* **365**, eaax1566 (2019). **Integrates AI-driven synthesis planning with robotic execution in flow, demonstrating an end-to-end pipeline from computational intent to physical chemistry outcomes**. 10.1126/science.aax1566 31395756

[51] Nambiar, A. M. K. et al. Bayesian optimization of computer-proposed multistep synthetic routes on an automated robotic flow platform. *ACS Cent. Sci.* **8**, 825–836 (2022). 35756374 10.1021/acscentsci.2c00207 PMC9228554

[52] MacLeod, B. P. et al. A self-driving laboratory advances the Pareto front for material properties. *Nat. Commun.* **13**, 995 (2022). 35194074 10.1038/s41467-022-28580-6 PMC8863835

[53] Szymanski, N. J. et al. An autonomous laboratory for the accelerated synthesis of novel materials. *Nature* **624**, 1–6 (2023). **Demonstrates autonomous materials synthesis with tight experiment–analysis coupling, providing a high-impact reference point for scalable closed-loop laboratory operation**. 10.1038/s41586-023-06734-w PMC10700133 38030721

[54] Dembski, S. et al. Establishing and testing a robot-based platform to enable the automated production of nanoparticles in a flexible and modular way. *Sci. Rep.* **13**, 1–10 (2023). 37454142 10.1038/s41598-023-38535-6 PMC10349877

[55] Slattery, A. et al. Automated self-optimization, intensification, and scale-up of photocatalysis in flow. *Science* **383**, eadj1817 (2024). 10.1126/science.adj1817 38271529

[56] Fleischer, H. et al. Automated robotic system for sample preparation and measurement of heavy metals in indoor dust using inductively coupled plasma mass spectrometry (ICP-MS). *Adv. Sci. Technol. Eng. Syst. J.* **7**, 139–151 (2022).

[57] Ozgulbas, D. Y. et al. Robotic pendant drop: containerless liquid for μs-resolved, AI-executable XPCS. *Light Sci. Appl* **12**, 1–10 (2023). 37596264 10.1038/s41377-023-01233-z PMC10439219

[58] Zhao, H. et al. A robotic platform for the synthesis of colloidal nanocrystals. *Nat. Synth.* **2**, 505–514 (2023).

[59] Koscher, B. A. et al. Autonomous, multiproperty-driven molecular discovery: from predictions to measurements and back. *Science* **382**, eadi1407 (2023). **Shows autonomous molecular discovery that iterates between prediction and measurement under multiple objectives, illustrating how autonomy supports complex, data-driven optimisation**. 10.1126/science.adi1407 38127734

[60] Volk, A. A. & Abolhasani, M. Performance metrics to unleash the power of self-driving labs in chemistry and materials science. *Nat. Commun.* **15**, 1–7 (2024). 38355564 10.1038/s41467-024-45569-5 PMC10866889

[61] Hysmith, H. et al. The future of self-driving laboratories: from human in the loop interactive AI to gamification. *Digit. Discov.* **3**, 621–636 (2024).

[62] Wolf, Á., Zsoldos, P., Széll, K. & Galambos, P. Towards robotic laboratory automation plug & play: reference architecture model for robot integration. *SLAS Technol.* 100168 10.1016/J.SLAST.2024.100168 (2024). 10.1016/j.slast.2024.100168 39098589

[63] Busboom, A. Automated generation of OPC UA information models—a review and outlook. *J. Ind. Inf. Integr.* **39**, 100602 (2024).

[64] Hornsteiner, M., Empl, P., Bunghardt, T. & Schönig, S. Reading between the lines: process mining on OPC UA network data. *Sensors* **24**, 4497 (2024). 39065898 10.3390/s24144497 PMC11280917

[65] Trifonov, H. & Heffernan, D. OPC UA TSN: a next-generation network for Industry 4.0 and IIoT. *Int. J. Pervasive Comput. Commun.* **19**, 386–411 (2023).

[66] Fedullo, T., Morato, A., Tramarin, F., Rovati, L. & Vitturi, S. A comprehensive review on time sensitive networks with a special focus on its applicability to industrial smart and distributed measurement systems. *Sensors* **22**, 1638 (2022). 35214541 10.3390/s22041638 PMC8879530

[67] Leeming, R. et al. Development of a digital twin for the prediction and control of supersaturation during batch cooling crystallization. *Ind. Eng. Chem. Res.* **62**, 11067–11081 (2023). 37484628 10.1021/acs.iecr.3c00371 PMC10360059

[68] Szilágyi, B., Eren, A., Quon, J. L., Papageorgiou, C. D. & Nagy, Z. K. Digital design of the crystallization of an active pharmaceutical ingredient using a population balance model with a novel size dependent growth rate expression. From development of a digital twin to in silico optimization and experimental validation. *Cryst. Growth Des.* **22**, 497–512 (2021).

[69] Barhate, Y., Kilari, H., Wu, W. L. & Nagy, Z. K. Population balance model enabled digital design and uncertainty analysis framework for continuous crystallization of pharmaceuticals using an automated platform with full recycle and minimal material use. *Chem. Eng. Sci.* **287**, 119688 (2024).

[70] Parekh, R., Benyahia, B. & Rielly, C. D. A global state feedback linearization and decoupling MPC of a MIMO continuous MSMPR cooling crystallization process. *Comput. Aided Chem. Eng.* **43**, 1607–1612 (2018).

[71] Urwin, S. J. et al. Digital process design to define and deliver pharmaceutical particle attributes. *Chem. Eng. Res. Des.* **196**, 726–749 (2023).

[72] Yuan, X., Nagy, Z. K. & Benyahia, B. A comprehensive framework for model evaluation and refinement using MBDoE estimability and structural identifiability: application to a crystallization process. *Chem. Eng. J. Adv.* **23**, 100823 (2025).

[73] Lo, S. et al. Review of low-cost self-driving laboratories in chemistry and materials science: the “frugal twin” concept. *Digit. Discov.* **3**, 842–868 (2024).

[74] Wolf, Á, Romeder-Finger, S., Széll, K. & Galambos, P. Towards robotic laboratory automation Plug & play: survey and concept proposal on teaching-free robot integration with the Lapp digital twin. *SLAS Technol.* **28**, 82–88 (2023). 36646253 10.1016/j.slast.2023.01.003

[75] Bai, J. et al. A dynamic knowledge graph approach to distributed self-driving laboratories. *Nat. Commun.* **15**, 1–14 (2024). 38263405 10.1038/s41467-023-44599-9 PMC10805810

[76] Dai, T. et al. Autonomous mobile robots for exploratory synthetic chemistry. *Nature* **635**, 890–897 (2024). 39506122 10.1038/s41586-024-08173-7 PMC11602721

[77] Kroemer, O., Niekum, S. & Konidaris, G. A review of robot learning for manipulation: challenges, representations, and algorithms. *J. Mach. Learn. Res.* **22**, 1–82 (2020).

[78] Tang, C. et al. Deep reinforcement learning for robotics: a survey of real-world successes. *Annu. Rev. Control Robot. Auton. Syst.* **8**, 153–188 (2025).

[79] Wu, J., Jin, Z., Liu, A., Yu, L. & Yang, F. A survey of learning-based control of robotic visual servoing systems. *J. Frankl. Inst.* **359**, 556–577 (2022).

[80] Radulov, N., Wright, A., Little, T., Cooper, A. I. & Pizzuto, G. FLIP: flowability-informed powder weighing. Preprint at https://arxiv.org/pdf/2506.03896 (2025).

[81] Angelopoulos, A., Verber, M., McKinney, C., Cahoon, J. & Alterovitz, R. High-accuracy injection using a mobile manipulation robot for chemistry lab automation. In *2023 IEEE/RSJ International Conference on Intelligent Robots and Systems (IROS)*, (ed. Gregg, B.) 10102–10109 (IEEE, 2023).

[82] Mazumder, A. et al. Towards next generation digital twin in robotics: Trends, scopes, challenges, and future. *Heliyon* **9**, e13359 (2023). 36825188 10.1016/j.heliyon.2023.e13359 PMC9941953

[83] Qian, J. et al. Digital Twin for Chemical Science: a case study on water interactions on the Ag(111) surface. *Nat. Comput Sci.* **5**, 793–800 (2025). 40866731 10.1038/s43588-025-00857-y PMC12457190

[84] Xie, Z., Liang, X. & Roberto, C. Learning-based robotic grasping: a review. *Front. Robot. AI* **10**, 1038658 (2023). 37082744 10.3389/frobt.2023.1038658 PMC10111055

[85] Suomalainen, M., Karayiannidis, Y. & Kyrki, V. A survey of robot manipulation in contact. *Rob. Auton. Syst.* **156**, 104224 (2022).

[86] AboZaid, Y. A., Aboelrayat, M. T., Fahim, I. S. & Radwan, A. G. Soft robotic grippers: a review on technologies, materials, and applications. *Sens. Actuators A Phys.* **372**, 115380 (2024).

[87] Mao, Q., Liao, Z., Yuan, J. & Zhu, R. Multimodal tactile sensing fused with vision for dexterous robotic housekeeping. *Nat. Commun.* **15**, 6871 (2024). 39127714 10.1038/s41467-024-51261-5 PMC11316753

[88] Haddadin, S. & Shahriari, E. Unified force-impedance control. *Int. J. Robot. Res.* **43**, 2112–2141 (2024).

[89] Walker, M., Pizzuto, G., Fakhruldeen, H. & Cooper, A. I. Go with the flow: deep learning methods for autonomous viscosity estimations. *Digit. Discov.* **2**, 1540–1547 (2023). 38013903 10.1039/d3dd00109a PMC10561544

[90] Pizzuto, G., De Berardinis, J., Longley, L., Fakhruldeen, H. & Cooper, A. I. SOLIS: autonomous solubility screening using deep neural networks. In *2022 International Joint Conference on Neural Networks (IJCNN)*, (eds Gori, M. & Sperduti, A.) 1–7, (IEEE, 2022).

[91] Zhu, Q. et al. Automated synthesis of oxygen-producing catalysts from Martian meteorites by a robotic AI chemist. *Nat. Synth.* **3**, 319–328 (2023).

[92] Li, J. et al. AIR-Chem: authentic intelligent robotics for chemistry. *J. Phys. Chem. A* **122**, 9142–9148 (2018). 30395457 10.1021/acs.jpca.8b10680

[93] Burger, B. et al. A mobile robotic chemist. *Nature* **583**, 237–241 (2020). 32641813 10.1038/s41586-020-2442-2

[94] Zhu, Q. et al. An all-round AI-Chemist with a scientific mind. *Natl. Sci. Rev.* **9**, nwac190 (2022). 10.1093/nsr/nwac190 PMC9674120 36415316

[95] Ubezio, B., Ergun, S. & Zangl, H. Realistic sensor simulations for the digital twin. *e i Elektrotech. Informationstech.* **140**, 562–571 (2023).

[96] Al-Tawil, B., Hempel, T., Abdelrahman, A. & Al-Hamadi, A. A review of visual SLAM for robotics: evolution, properties, and future applications. *Front. Robot. AI* **11**, 1347985 (2024). 38686339 10.3389/frobt.2024.1347985 PMC11056647

[97] Guan, J., Hao, Y., Wu, Q., Li, S. & Fang, Y. A survey of 6DoF object pose estimation methods for different application scenarios. *Sensors* **24**, 1076 (2024). 38400234 10.3390/s24041076 PMC10893425

[98] Nakajima, Y. et al. Robotic powder grinding with audio–visual feedback for laboratory automation in materials science. In *2023 IEEE/RSJ International Conference on Intelligent Robots and Systems (IROS)*, (ed. Gregg, B.) 8283–8290 (IEEE, 2023).

[99] Nakajima, Y. et al. *Robotic Powder Grinding With a Soft Jig for Laboratory Automation in Material Science* 2320–2326 (IEEE/RJS International, 2022).

[100] Lunt, A. M. et al. Modular, multi-robot integration of laboratories: an autonomous workflow for solid-state chemistry. *Chem. Sci.* **15**, 2456–2463 (2024). 38362408 10.1039/d3sc06206f PMC10866346

[101] Jiang, J., Cao, G., Deng, J., Do, T.-T. & Luo, S. Robotic perception of transparent objects: a review. *IEEE Trans. Artif. Intell.* **5**, 2547–2567 (2024).

[102] Cheng, X. et al. Intelligent vision for the detection of chemistry glassware toward AI robotic chemists. *Artif. Intell. Chem.* **1**, 100016 (2023).

[103] Garrett, C. R. et al. Integrated task and motion planning. *Annu. Rev. Control Robot. Auton. Syst.* **4**, 265–293 (2021).

[104] Angelopoulos, A., Cahoon, J. F. & Alterovitz, R. Transforming science labs into automated factories of discovery. *Sci. Robot.* **9**, 6991 (2024). 10.1126/scirobotics.adm6991 39441898

[105] Yoshikawa, N. et al. An adaptive robotics framework for chemistry lab automation. Preprint at 10.48550/arXiv.2212.09672 (2022).

[106] Snapp, K. L. & Brown, K. A. Driving school for self-driving labs. *Digit. Discov.* **2**, 1620–1629 (2023).

[107] Stodden, V. et al. Enhancing reproducibility for computational methods. *Science (1979)* **354**, 1240–1241 (2016). 10.1126/science.aah6168 27940837

[108] Wilkinson, M. D. et al. The FAIR Guiding Principles for scientific data management and stewardship. *Sci. Data* **3**, 160018 (2016). 26978244 10.1038/sdata.2016.18 PMC4792175

[109] Villani, V., Pini, F., Leali, F. & Secchi, C. Survey on human–robot collaboration in industrial settings: safety, intuitive interfaces and applications. *Mechatronics* **55**, 248–266 (2018).

[110] Mittelstadt, B. Principles alone cannot guarantee ethical AI. *Nat. Mach. Intell.* **1**, 501–507 (2019).

[111] Floridi, L. & Cowls, J. A unified framework of five principles for AI in society. *Harv. Data Sci. Rev.* **1**, (2019).

[112] Gebru, T. et al. Datasheets for datasets. *Commun. ACM* **64**, 86–92 (2021).

[113] Urbina, M. A., Watts, A. J. R. & Reardon, E. E. Labs should cut plastic waste too. *Nature* **528**, 479–479 (2015). 26701046 10.1038/528479c

[114] Ince, M. C., Benyahia, B. & Vilé, G. Sustainability and techno-economic assessment of batch and flow chemistry in seven industrial pharmaceutical processes. *ACS Sustain. Chem. Eng.* **13**, 2864–2874 (2025). 40018297 10.1021/acssuschemeng.4c09289 PMC11864096

[115] Liu, J., Zhang, D., Liu, Z., Guo, T. & Yan, Y. Construction of a digital twin system and dynamic scheduling simulation analysis of a flexible assembly workshops with Island layout. *Sustainability* **16**, 8851 (2024).

[116] Bran, M. A. et al. Augmenting large language models with chemistry tools. *Nat. Mach. Intell.* **6**, 525–535 (2024). 38799228 10.1038/s42256-024-00832-8 PMC11116106

[117] Zhou, J. et al. A multi-robot-multi-task scheduling system for autonomous chemistry laboratories. *Digit. Discov.* **4**, 636–652 (2025).

[118] Zhu, C., Dastani, M. & Wang, S. A survey of multi-agent deep reinforcement learning with communication. *Auton. Agent Multi Agent Syst.* **38**, 4 (2024).

[119] Zhao, Z. et al. A survey of optimization-based task and motion planning: from classical to learning approaches. *IEEE/ASME Trans. Mechatron.* **30**, 2799–2825 (2025).

[120] Wan, W., Kotaka, T. & Harada, K. Arranging test tubes in racks using combined task and motion planning. *Rob. Auton. Syst.* **147**, 103918 (2022).

[121] Orthey, A., Chamzas, C. & Kavraki, L. E. Sampling-based motion planning: a comparative review. *Annu. Rev. Control Robot Auton. Syst.* **7**, 285–310 (2024).

[122] Zhang, T. et al. Time-Sensitive Networking (TSN) for industrial automation: current advances and future directions. *ACM Comput Surv.* **57**, 1–38 (2025).

[123] Tsiotras, P., Gombolay, M. & Foerster, J. Editorial: Decision-making and planning for multi-agent systems. *Front. Robot. AI* **11**, 1422344 (2024). 38855625 10.3389/frobt.2024.1422344 PMC11157335

[124] Shakeri, Z., Benfriha, K., Varmazyar, M., Talhi, E. & Quenehen, A. Production scheduling with multi-robot task allocation in a real industry 4.0 setting. *Sci. Rep.* **15**, 1795 (2025). 39805942 10.1038/s41598-024-84240-3 PMC11729899

[125] Gao, S. et al. Empowering biomedical discovery with AI agents. *Cell* **187**, 6125–6151 (2024). 39486399 10.1016/j.cell.2024.09.022

[126] Jin, P., Lin, Y., Song, Y., Li, T. & Yang, W. Vision-force-fused curriculum learning for robotic contact-rich assembly tasks. *Front. Neurorobot.* **17**, 1280773 (2023). 37867617 10.3389/fnbot.2023.1280773 PMC10590057

[127] Hwang, P.-J., Hsu, C.-C., Chou, P.-Y., Wang, W.-Y. & Lin, C.-H. Vision-based learning from demonstration system for robot arms. *Sensors* **22**, 2678 (2022). 35408292 10.3390/s22072678 PMC9002941

[128] Barekatain, A., Habibi, H. & Voos, H. A practical roadmap to learning from demonstration for robotic manipulators in manufacturing. *Robotics* **13**, 100 (2024).

[129] Yang, X., Zhou, Z., Li, L. & Zhang, X. Collaborative robot dynamics with physical human–robot interaction and parameter identification with PINN. *Mech. Mach. Theory* **189**, 105439 (2023).

[130] Iliuţă, M.-E. et al. Digital Twin—a review of the evolution from concept to technology and its analytical perspectives on applications in various fields. *Appl. Sci.* **14**, 5454 (2024).

[131] Nambiar, S., Paul, R. C., Ikechukwu, O. C., Jonsson, M. & Tarkian, M. Digital twin-enabled adaptive robotics: leveraging large language models in Isaac Sim for unstructured environments. *Machines* **13**, 620 (2025).

[132] Callari, T. C., Vecellio Segate, R., Hubbard, E.-M., Daly, A. & Lohse, N. An ethical framework for human–robot collaboration for the future people-centric manufacturing: a collaborative endeavour with European subject-matter experts in ethics. *Technol. Soc.* **78**, 102680 (2024).

[133] Haney, J. M. & Liang, C. J. A literature review on safety perception and trust during human–robot interaction with autonomous mobile robots that apply to industrial environments. *IISE Trans. Occup. Ergon. Hum. Factors* **12**, 6–27 (2024). 38190192 10.1080/24725838.2023.2283537 PMC11076167

[134] Balakrishnan, M., Ferreira, K. J. & Tong, J. Human–algorithm collaboration with private information: naïve advice-weighting behavior and mitigation. *Manage. Sci.*10.1287/mnsc.2022.03850 (2025).

[135] Sanders, N. E., Şener, E. & Chen, K. B. Robot-related injuries in the workplace: an analysis of OSHA severe injury reports. *Appl. Erg.* **121**, 104324 (2024). 10.1016/j.apergo.2024.104324 39018706

[136] Gunes, H. et al. Reproducibility in human–robot interaction: furthering the science of HRI. *Curr. Robot. Rep.* **3**, 281–292 (2022). 36311257 10.1007/s43154-022-00094-5 PMC9589714

[137] Faragasso, A. & Bonsignorio, F. Reproducibility challenges in robotic surgery. *Front. Robot. AI* **10**, 1127972 (2023). 37008982 10.3389/frobt.2023.1127972 PMC10050429

[138] Senoner, J., Schallmoser, S., Kratzwald, B., Feuerriegel, S. & Netland, T. Explainable AI improves task performance in human–AI collaboration. *Sci. Rep.* **14**, 31150 (2024). 39730794 10.1038/s41598-024-82501-9 PMC11681242

[139] Brunke, L. et al. Safe learning in robotics: from learning-based control to safe reinforcement learning. *Annu. Rev. Control Robot Auton. Syst.* **5**, 411–444 (2022).

[140] Häse, F., Roch, L. M. & Aspuru-Guzik, A. Next-generation experimentation with self-driving laboratories. *Trends Chem.* **1**, 282–291 (2019).

[141] Granda, J. M., Donina, L., Dragone, V., Long, D.-L. & Cronin, L. Controlling an organic synthesis robot with machine learning to search for new reactivity. *Nature* **559**, 377–381 (2018). 30022133 10.1038/s41586-018-0307-8 PMC6223543

[142] Maffettone, P. M., Campbell, S., Hanwell, M. D., Wilkins, S. & Olds, D. Delivering real-time multi-modal materials analysis with enterprise beamlines. *Cell Rep. Phys. Sci.* **3**, 101112 (2022).

