# Supplementary material for: The ADePT framework for assessing autonomous laboratory robotics
Source: Commun Chem. 2026 Feb 20;9:99. doi: 10.1038/s42004-026-01932-9 (PMC12923569; doi:10.1038/s42004-026-01932-9)
Supplement: Supplementary file 1 — Supplementary material pdf 26-01-26 [file 42004_2026_1932_MOESM1_ESM.pdf]

Supplementary Material: The ADePT Framework for Assessing Autonomous Laboratory Robotics

Pablo Salazar-Villacis and Brahim Benyahia
School of AACME; Department of Chemical Engineering
Loughborough University
Loughborough, United Kingdom

SM1. Evolution of Research Activity in Robots in the context of Lab automation and Self-Driving Laboratories across the Chemical Sciences

To contextualise the emergence and disciplinary adoption of self-driving laboratories, we conducted a bibliometric analysis of the Web of Science (WoS) database covering the period from 2012 to 2024. The analysis aimed to track the growth in research output and identify shifts in disciplinary focus over time. Publications were categorised into four research areas: Chemistry, Materials Science, Biochemistry & Molecular Biology, and Others, to highlight the evolving landscape and adoption of autonomous laboratory systems across adjacent scientific domains.

Supplementary Figure 1 | Growth and disciplinary distribution of publications related to self-driving laboratories

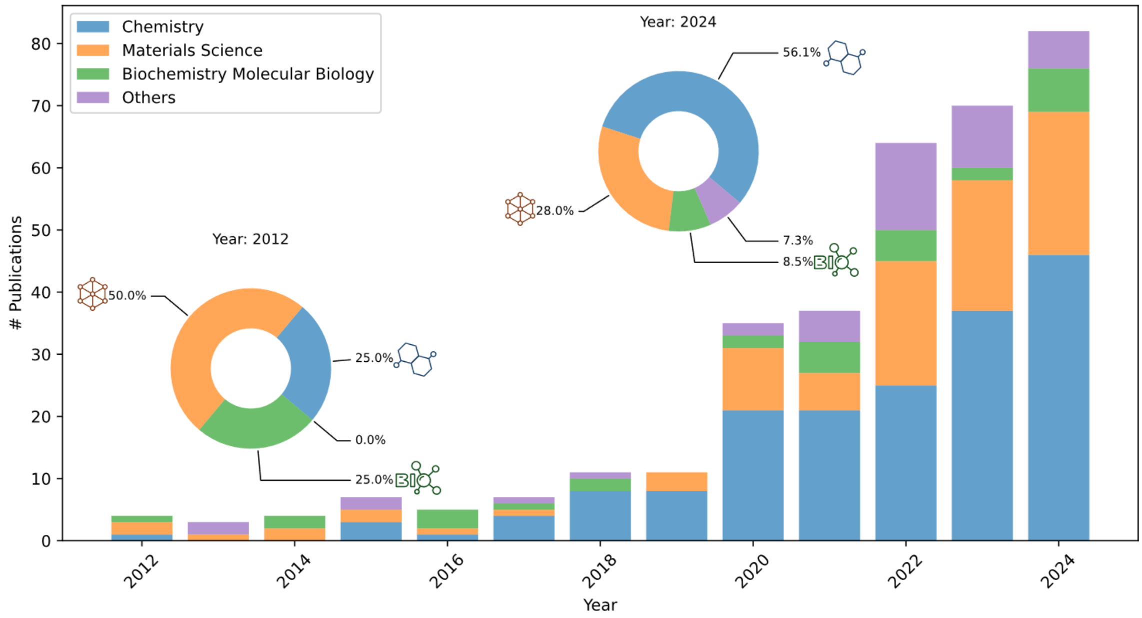

Supplementary Figure 1 Growth and disciplinary distribution of publications related to self-driving laboratories (2012–2024). Annual publication counts and disciplinary composition of research related to self-driving laboratories, based on a bibliometric analysis of the Web of Science Core Collection. Stacked bars show the number of publications per year across four research areas, while inset pie charts illustrate the relative disciplinary distribution in 2012 and 2024.

We performed a series of refined queries on the Web of Science Core Collection, targeting publications related to self-driving laboratories in the chemical and materials sciences. The base query required the terms Robot* and Lab* to appear in any field. Results were further filtered to include only specific research areas relevant to the life and physical sciences (e.g., Chemistry, Materials Science, Biochemistry & Molecular Biology, Crystallography, Electrochemistry, Polymer Science), while excluding review articles and duplicate research area overlaps.

Each publication was assigned to one of four categories:

- Chemistry: Publications explicitly classified under "Chemistry" and not under Materials Science or Biochemistry.
- Materials Science: Publications listed only under "Materials Science" (excluding Chemistry and Biochemistry).
- Biochemistry & Molecular Biology: Publications specifically listed under that research area, excluding Chemistry and Materials Science.
- Others: Publications falling outside the above three categories, including cross-disciplinary areas such as Biotechnology, Pharmacology, or Oncology.

The publication trends over the past decade reveal a pronounced acceleration in the field of self-driving laboratories, with the annual number of publications increasing more than tenfold from 2012 to 2024. This growth reflects the maturation of enabling technologies such as robotic platforms, machine learning algorithms, and automated analytical infrastructure, as well as a growing recognition of automation's role in accelerating discovery.

Disciplinary contributions have evolved markedly over time. In the early years (2012–2016), the field was largely driven by materials science, accounting for up to 50% of publications, likely reflecting early adoption of robotic platforms for high-throughput materials screening and device fabrication. Chemistry, by contrast, was underrepresented, comprising only 25% of studies in 2012.

However, this landscape has shifted. By 2024, chemistry has become the dominant discipline, comprising over 56% of new publications. This shift signals the field’s increasing engagement with core chemical reactivity, synthesis, and catalysis workflows, domains traditionally viewed as less automatable. Advances in precision liquid handling, closed-loop optimisation, and algorithm-guided reaction planning have likely contributed to this surge.

Biochemistry and other life science disciplines have maintained a relatively small but steady presence. Their slower uptake may reflect the greater complexity of biological systems, sample variability, or the stringent sterility and environmental controls required for automation in these settings.

These results suggest that self-driving laboratories have transitioned from a technological novelty rooted in materials characterisation to a broad methodological framework with increasing traction in synthetic chemistry. The future growth of the field may depend on further generalisation to biologically and clinically relevant workflows, as well as the integration of more adaptive robotic architectures and richer multi-modal sensing.

SM2. Evaluation Criteria: ADePT Framework

This supplementary section introduces the framework used to systematically evaluate the autonomy of robotic systems in laboratory environments. To quantify autonomy in a consistent and reproducible manner, we developed a rubric structured around four core components: Adaptability and Learning, Dexterity, Perception, and Task Complexity. Each component encompasses distinct sub-metrics, scored on a continuous scale from 0 to 1. These sub-metrics assess increasingly sophisticated capabilities, from minimal automation to high degrees of autonomous function. Unless otherwise stated, each dimension score is the arithmetic mean of its sub-indicators, and any optional overall ADePT index is the unweighted mean of the four dimensions; application-specific weights may be applied by readers according to domain priorities

Adaptability and Learning, Dexterity, Perception and Task Complexity act together in closed loop laboratory operation. Perception determines what can be observed and estimated during execution, while Dexterity determines what sensed information can be turned into safe, precise action in contact-rich workcells; surveys of visual servoing and manipulation in contact show that routing sensing into control is essential for reliable behaviour [1,2]. Advances in grippers and grasp learning expand feasible contact modes and reduce sensitivity to perceptual noise, linking sensing and action at the hardware–policy boundary [3,4]. Adaptability and Learning depend on these channels to express updates during experiments, and rapid adaptation with online identification demonstrates that policies and model parameters can change during execution when informative feedback and safe exploration are available [5,6]. Task Complexity then sets the temporal and structural load on the system: more subtasks and greater uncertainty increase the demands on observation, control and learning, while cross-embodiment and multi-task models provide evidence that broader variability tolerance supports longer, more reliable routines [7–9]. Together these strands justify the ADePT structure by capturing the observable state, the actionable state, the operational load and the mechanism of improvement in a minimal set for comparable scoring across platforms [10,11].

The rubric was applied uniformly across a diverse set of peer-reviewed case studies, allowing for comparative analysis and identification of technological gaps and trends in laboratory automation. The scoring criteria are grounded in both theoretical benchmarks

of robotic autonomy and empirical evidence reported in the literature. Full justifications for each score are provided alongside excerpts from the original publications.

Because ADePT dimensions target complementary capabilities, we report them separately to preserve trade-offs; qualitative interrelations are discussed in SM2 and quantitative co-variation can be inspected across cases in SM4.

SM2.1. Adaptability and Learning

The *Adaptability and Learning* criterion evaluates a robotic system's capacity to respond to new tasks, changing environments, and unexpected conditions during operation. Adaptability is central to the development of autonomous systems capable of functioning beyond rigid, pre-defined procedures [11–14].

The assessment considers four subcomponents: Adaptation Efficiency, Task Generalisation, Real-Time Adaptability, and Learning Method. Adaptation efficiency refers to how readily the robot can accommodate changes without manual intervention. Task generalisation reflects the robot's ability to apply previously learned behaviours to different but related tasks. Real-time adaptability measures whether the system can adjust its actions dynamically in response to sensor feedback or environmental shifts. The learning method captures the sophistication of the approach used to improve performance, ranging from fixed rules to advanced machine learning algorithms [6,9,15–17].

In closed loop laboratory campaigns, learning produces useful behaviour only if updates are expressed during ongoing experiments [12,13]. Real time adaptability governs the latency and cadence with which sensing becomes action, while adaptation efficiency records whether those updates actually restore or improve performance under routine shifts such as batch variation or instrument drift. Task generalisation supplies the prior that makes adaptation efficient, since policies trained across diverse tasks and embodiments start closer to workable solutions for new procedures[8,9]. The learning method ties these pieces together, because identification based controllers, diffusion and transformer sequence models, and reinforcement learning determine both what is learnable and how safely and quickly behaviour improves[6,16,18,19]. Recent reports and surveys describe this feedback loop in practice across chemistry and robotics, linking generalisation and online updating to reliable closed loop operation [14,17].

At the lowest performance level, robots are entirely scripted and require reprogramming for any change. Higher scores are awarded to systems that can adapt autonomously, generalise learned strategies across workflows, respond fluidly in real time, and incorporate learning strategies such as reinforcement learning or deep learning to refine behaviours during operation [9,15,17].

This framework distinguishes between static automation and adaptive autonomy, providing a clear basis for evaluating how learning contributes to flexible, robust robotic performance in laboratory settings[11,13,14].

Supplementary Table 1 Adaptability and Learning scoring criteria

Adaptation Efficiency	Task Generalisation	Real-Time Adaptability	Learning Method	Score
No ability to adapt; robot requires full reprogramming for new tasks or changes.	No task generalisation; robot can only perform one specific task and cannot generalise learned behaviours.	No real-time adaptability; robot performs rigid, pre-programmed actions without adjustments.	No learning method; robot performs fixed actions based on pre-programmed instructions.	0
Very slow or inefficient adaptation; robot struggles to adapt and requires significant reprogramming.	Very limited task generalisation; robot struggles to apply learned skills to even closely related tasks.	Minimal real-time adaptability; robot makes slow or infrequent adjustments based on feedback	Basic rule-based learning; robot applies simple, fixed rules for task variations.	0.2
Limited adaptability; robot can adjust to minor changes but still requires manual intervention.	Limited task generalisation; robot can generalise to a few related tasks but requires reprogramming for others.	Limited real-time adaptability; robot adjusts to some feedback but with noticeable delays.	Limited learning ability; robot uses basic algorithms to adjust its actions but cannot autonomously learn new tasks.	0.4
Moderate adaptability; robot can efficiently adjust to task variations without much intervention.	Moderate task generalisation; robot can generalise skills learned in one task to several related tasks.	Moderate real-time adaptability; robot can adjust to feedback during task execution with reasonable efficiency.	Moderate learning ability; robot uses machine learning or similar methods to adjust and improve task performance.	0.6
Good adaptability; robot adjusts quickly and efficiently to task changes, with minimal reprogramming.	Good task generalisation; robot applies learned skills to a wide range of related tasks.	Strong real-time adaptability; robot makes near real-time adjustments to feedback, improving task performance.	Strong learning method; robot employs advanced learning algorithms (e.g., reinforcement learning) to autonomously improve task performance.	0.8
Highly efficient adaptation; robot autonomously	Excellent task generalisation; robot can autonomously	Fully autonomous real-time adaptability; robot	Highly advanced learning method; robot uses cutting-edge	1

adapts to new tasks or environmental changes in real-time without reprogramming.	transfer learned skills across many different tasks without reprogramming	continuously adjusts its actions based on feedback without delays or errors.	techniques (e.g., deep learning) to autonomously learn and optimise new tasks continuously.	

SM2.2. Dexterity

The *Dexterity* criterion evaluates the physical manipulation capabilities of robotic systems used in laboratory environments[11,13,14]. Effective manipulation is essential for executing experimental procedures that involve handling diverse labware, performing delicate tasks, and adapting to variable geometries and materials[3,4,20–22].

Three subcategories are considered: Degrees of Freedom (DOF), Force Control, and Grasp Variety. Degrees of freedom reflect the mechanical flexibility of the robot, determining how freely it can move and orient its end-effector[23–25]. Force control assesses the system's ability to regulate applied forces during interactions, which is especially important when handling fragile or sensitive materials[26–28]. Grasp variety measures the range of object types the robot can successfully pick up and manipulate [3,4,20,21].

In laboratory manipulation, dexterity arises from the interaction of configuration freedom, contact regulation, and grasp repertoire. Degrees of freedom set whether the end effector can realise task frames and avoid singularities in crowded benches, linking workspace coverage and manipulability to success with varied labware [23–25]. Force control then converts reach into safe, precise interaction by shaping impedance and contact transitions so fragile glassware, seals and fittings can be handled without damage[27,28]. Grasp variety supplies the stable contact modes available to the system, allowing it to cope with different geometries, materials and deformabilities from rigid vials and microplates to flexible tubing and soft packs [3,4,21]. Surveys of manipulation in contact and in-hand control emphasise that progress in one dimension reinforces the others, since richer grasps expand feasible force strategies and higher configuration freedom exposes poses where force regulation is effective [2,22]. In autonomous laboratory case studies these dimensions co-occur in practice, where multi-instrument workflows succeed when robots can reach and reorient in confined spaces, modulate interaction forces for delicate transfers, and switch grasp strategies as tools and samples change during procedures, which is why dexterity tracks experimental reliability in real deployments.

At the lowest level, systems are limited to simple, rigid operations with little or no adaptability. As performance improves, robots demonstrate increasingly precise motion, controlled application of force, and the ability to handle a broader set of tools and

samples. The highest scores correspond to systems that combine fine motor control, real-time force adjustment, and the versatility to manipulate objects with varied and complex properties[4,27].

This rubric allows for consistent benchmarking of robotic dexterity in the context of laboratory automation and highlights the capabilities required for autonomous systems to perform tasks reliably and safely[2,11].

Supplementary Table 2 Dexterity scoring criteria

Degrees of Freedom (DOF)	Force Control	Grasp Variety	Score
No dexterity or extremely limited (e.g., single-axis motion).	No force control; robot applies a fixed or binary grip force, unable to handle delicate tasks	Very limited grasping ability; can only handle one type of object shape or size.	0
Very limited DOF (e.g., basic gripper with 2-3 DOF) allowing only simple, linear movements.	Minimal force control; robot has basic pressure adjustments but lacks fine control over applied forces.	Limited grasping variety; can handle objects of a single shape (e.g., cylindrical) or material.	0.2
Moderate DOF (e.g., 3-4 DOF), capable of basic manipulations but not complex movements.	Some force control; robot can adjust grip force to some extent but still risks damaging delicate objects.	Moderate grasping ability; robot can handle a few different types of objects but struggles with complex shapes or materials.	0.4
Sufficient DOF (e.g., 5-6 DOF) for flexible movements, such as rotating, twisting, and performing multiple types of grasps.	Good force control; robot can apply precise forces during manipulation, avoiding damage to objects but not suitable for very sensitive tasks.	Good grasp variety; robot can grasp a wide range of objects of different sizes, shapes, and materials.	0.6
High DOF (e.g., 6-7 DOF), enabling complex and flexible manipulations similar to a human hand	Strong force control; robot adjusts its grip and manipulation forces dynamically and precisely, able to handle delicate objects.	High grasp variety; robot can handle complex objects and materials, including soft, hard, and irregularly shaped objects.	0.8
Extremely high DOF (e.g., 7+ DOF), allowing for highly intricate movements and manipulations, capable of handling complex tasks like human dexterity.	Highly advanced force control with real-time adjustments; robot can handle extremely fragile objects without causing damage, even in complex manipulations.	Extremely versatile grasping capability; robot can grasp almost any object, regardless of size, shape, or material, with precision.	1

SM2.3. Perception

The *Perception* criterion assesses the extent to which a robotic system can sense and respond to its surroundings during task execution[10]. This capability is fundamental for enabling autonomy in laboratory environments, where variability in samples, tools, and spatial configurations can affect performance[13,29,30].

The evaluation is based on three dimensions: Sensor Variety, Feedback Integration, and Environmental Awareness. These dimensions reflect whether the system uses a diverse set of sensors, whether sensor data are integrated to adjust actions, and whether the robot has any functional understanding of its environment[2,31]. Sensor Variety broadens what can be observed, for example by fusing vision with tactile cues to cover object identity, pose and contact states [32]. Feedback Integration turns those signals into action through closed-loop control, as surveyed for visual servoing and contact-rich manipulation [1,2]. Environmental Awareness maintains a persistent world model so that fused signals remain actionable over time, with reviews highlighting mapping and localisation as well as 6-DoF object pose estimation in changing work cells [33,34].

At the lowest level, systems operate in open-loop mode with no meaningful sensing or feedback. Moderate scores indicate some integration of sensors such as cameras or force sensors, and basic responses to environmental cues, although typically in structured or static settings[1,35]. The highest scores are reserved for systems that combine multiple sensor types into a coherent framework, perform continuous feedback-based adjustments, and demonstrate an ability to map and interpret complex, dynamic environments[32–34,36].

This classification enables comparison across platforms and identifies areas where perceptual capabilities remain limited in current laboratory automation[11].

Supplementary Table 3 Perception scoring criteria

Sensor Variety	Feedback Integration	Environmental Awareness	Score
No sensors are used or only basic encoders (e.g., position encoders).	No integration of feedback; robot actions are pre-programmed without adjustments based on sensor input.	No environmental awareness; robot operates purely based on predefined instructions without sensing its surroundings.	0
Very limited sensors, such as a single camera or simple range sensor, with minimal use in tasks.	Very limited feedback integration; occasional sensor input is used, but actions are mostly static.	Minimal awareness; robot can detect basic object presence, but no awareness of the environment's structure or changes.	0.2

A small number of sensors, such as basic visual and force sensors, but lacking diversity (e.g., no tactile or multi-modal sensing).	Some feedback integration is present, but adjustments are slow or not responsive enough for real-time corrections.	Limited environmental awareness; robot can detect and respond to some features but struggles in dynamic or complex environments.	0.4
Multiple sensors (e.g., visual and force) that provide sufficient input for task performance, but lacking tactile or specialised sensors.	Moderate feedback integration, allowing for reasonable real-time adjustments during task execution.	Reasonable awareness; robot can detect and understand its surroundings in controlled environments, with moderate adaptation to changes.	0.6
A diverse set of sensors, including visual, force, and some tactile feedback, allowing robust interaction with objects and environments.	Strong feedback integration, enabling near real-time adjustments to handle varying conditions during tasks.	Good awareness; robot can map and understand its environment, detecting changes and adapting its actions accordingly.	0.8
Highly diverse sensors (e.g., vision, force, tactile, thermal) integrated into a coherent system that provides comprehensive environmental and task-related data.	Fully integrated feedback system, where real-time adjustments are made continuously and seamlessly to optimize task performance.	Highly advanced environmental awareness; robot can fully perceive, map, and interpret complex, dynamic environments and respond autonomously to unexpected changes.	1

SM 2.4. Task Complexity

The *Task Complexity* criterion captures the scope and difficulty of the tasks a robotic system is capable of performing in a laboratory setting[37]. This dimension is essential for assessing how well a system can support realistic experimental workflows, which often involve multiple steps, uncertain conditions, and diverse procedures[13,14].

Three aspects are considered: Number of Subtasks, Environmental Uncertainty, and Task Variability. The number of subtasks reflects how many sequential or parallel actions are required, such as object handling, preparation steps, or analysis procedures [7]. Environmental uncertainty accounts for the robot's ability to operate in conditions that may not be fully controlled, including spatial variability or dynamic changes in the workspace[2]. Task variability measures how broadly a robot can apply its capabilities across different procedures, whether it is restricted to a single repetitive task or able to

manage a wide variety of experimental operations[8,9]. In practice these three aspects form a single load on capability. As the number of subtasks grows, the discrete search over action sequences and the continuous feasibility checks both expand, which is the classic hallmark of long horizon planning and task and motion planning complexity [7]. Environmental uncertainty compounds this load by introducing belief updates, re-planning and contact management within those longer procedures, so execution must remain robust to partial observability and process drift typical of lab workcells [2,38]. Task variability then sets how far skills transfer across different protocols and tools, which directly influences how efficiently long procedures can be assembled and recovered when conditions shift; large multitask and cross robot studies show that broader variability tolerance enables composition of longer, more reliable routines [8,9]. Long horizon benchmarks that require many coordinated steps make the same interdependence visible, since more subtasks and more diverse parts elevate the effect of uncertainty on success rates [39].

Low-scoring systems are limited to basic, linear routines in stable environments with no variation in task type. As complexity increases, robots demonstrate the ability to execute longer and more intricate procedures, adapt to changing surroundings, and flexibly transition between task types. At the highest level, systems autonomously perform complex workflows across domains, operating reliably in dynamic, unpredictable settings[40,41].

This framework allows a structured assessment of the functional breadth and operational resilience of robotic systems in the context of chemical research.

Supplementary Table 4 Task complexity scoring criteria

Number of Subtasks	Environmental Uncertainty	Task Variability	Score
No subtask complexity; robot performs only simple, single-step tasks..	No uncertainty; robot operates in a completely controlled, unchanging environment.	No task variability; robot performs only one fixed task repeatedly.	0
Very simple tasks with one or two subtasks, such as picking and placing.	Minimal uncertainty; robot operates in a mostly controlled environment with minor changes.	Very limited variability; robot can handle a small range of similar tasks.	0.2
Simple tasks with a few sequential steps, such as pick, place, and position.	Some environmental uncertainty; robot can handle limited variability, such as slight object displacement.	Some task variability; robot can perform a moderate variety of tasks with similar characteristics	0.4
Moderate complexity with several subtasks,	Moderate environmental	Reasonable task variability; robot can	0.6

such as multi-object assembly or transfer.	uncertainty; robot can adapt to moderate changes, such as dynamic object positions..	handle a wide range of different tasks within a similar domain.	
High task complexity with many sequential steps, involving multiple actions, objects, or stages.	High environmental uncertainty; robot can handle dynamic, partially unstructured environments with changing variables.	High task variability; robot can handle a diverse set of tasks across different domains.	0.8
Extremely high task complexity, involving multiple layers of actions, decision-making, and subtasks, such as assembling complex objects with many parts.	Extremely high environmental uncertainty; robot autonomously navigates highly dynamic, unstructured environments with unpredictable changes.	Extremely high task variability; robot can perform a wide variety of complex, unrelated tasks with minimal adjustment.	1

SM3. Analysis of the Literature under the ADePT Framework.

To assess the state of autonomy in laboratory robotics, we reviewed and scored 36 representative case studies using the evaluation rubric. Each study was examined for evidence of autonomous capability in the areas of **Perception**, **Dexterity**, **Adaptability and Learning**, and **Task Complexity**.

- **Adaptability and Learning**: This remains the most underdeveloped category, with many systems relying on fixed scripts or human-programmed routines. Only a small subset incorporates real-time adaptability or learning-based strategies. Notable exceptions include Kadokawa et al. (2023) and Song et al. (2024), which leverage reinforcement learning and LLM-driven planning, respectively.
- **Dexterity**: While several robotic arms offer high degrees of freedom (6–7 DOF), few employ nuanced force control or demonstrate a wide range of grasping behaviours. Robotic platforms such as those by Pai et al. (2023) and Pizzuto et al. (2022a) stand out for combining advanced DOF with feedback-enabled manipulation tasks.
- **Perception**: Most systems demonstrate limited sensor diversity, relying heavily on basic visual or positional sensors. A minority integrate multi-modal perception, including tactile and force feedback. Only a few, notably those by Butterworth et al. (2023) and Zhu et al. (2022), approached higher perception scores through the use of combined visual, force, and tactile sensing or SLAM-based environmental mapping.

- **Task Complexity**: While most systems automate linear workflows with minimal task variation or environmental uncertainty, a few demonstrate integration across multiple workstations or workflows. For instance, Darvish et al. (2024) and Lunt et al. (2024) exhibit high levels of procedural complexity and parallel task execution.

This structured analysis provides a comprehensive view of the autonomy landscape in the robotics component of self-driving labs, highlighting both current capabilities and areas for future development. A full breakdown of scores and justifications is presented in the accompanying subsections.

SM3.1. ADePT Evaluation for Robot as Systems Integrators Use Cases

Supplementary Table 5 ADePT Evaluation for "Flexible Laboratory Automation System Based on Distributed Framework: Implementation for Press Process in Polymer Materials Development" [42]

Reference	Use case	Robotic System
**Asano et.al 2023**	A robot automates loading/unloading in a polymer press process, while the pressing itself is optimized through BO for process parameters.	A 7-DOF Franka Emika robot arm is used to manipulate plates and polymer samples, handling the physical steps of loading/unloading a PLC-controlled press machine

Dimension	Metric	Justification	Paper Evidence	Score
**Adaptability and Learning**	Adaptation Efficiency	Adaptation applies only to the process parameters (through BO) and not to the robot's own tasks. The robot's actions remain pre-programmed and unadaptive.	The robot performs fixed actions, and BO optimizes the process parameters, not robot behavior.	0
	Task Generalization	The robot is designed for a specific task—handling materials during the polymer press process—and there is no evidence of skill generalization to other tasks.	The system is specialized for polymer pressing, and the robot's role is tightly bound to this task.	0.2
	Real-Time Adaptability	The robot follows pre-defined actions and does not adapt in real time to changes during the task. Parameter adjustments via BO are post-process, not real-time.	The robot does not adapt its actions in real time, and any adjustments are made between experimental runs .	0
	Learning Method	There is no learning method applied to the robot's tasks; the learning via BO pertains only to the process parameters of the press machine.	"The parameters of the press machine... are determined using BO". The robot's actions are not subject to learning.	0

**Dexterity**	Degrees of Freedom (DOF)	The robot arm has 7 DOF, allowing flexibility in manipulating objects like plates and loading/unloading the press machine.	"The robot arm is Panda (Franka Emika), which is a collaborative robot with 7DOFs."	0.7
	Force Control	No mention of force control, suggesting the robot uses pre-programmed movements without dynamic force adjustments.	No force-sensitive manipulation is mentioned .	0
	Grasp Variety	The robot handles plates and polymer samples, but there is no indication of manipulating various objects of different shapes or sizes.	"The robot arm manipulates tools and polymer granules placed on the workstand".	0.4
**Perception**	Sensor Variety	The robot employs a camera for visual feedback but does not use advanced sensors (e.g., tactile or force sensors) to enhance task execution.	"The molded polymer is captured by a camera attached to the end-effector of the robot, and the polymer region is recognized by image recognition."	0.1
	Feedback Integration	Limited to feedback from the vision system for evaluating the polymer after the press, with no real-time sensor feedback during manipulation tasks.	Vision-based recognition is used to evaluate the polymer, but there is no real-time feedback affecting robot movements .	0.2
	Environmental Awareness	The robot recognizes specific objects (plates, polymer), but no broader environmental perception or adaptation to environmental changes is reported.	The robot senses the polymer and plates using a camera but lacks environmental awareness beyond this.	0.4
**Task Complexity**	Number of Subtasks	The robot handles straightforward subtasks such as positioning plates in the press machine and retrieving samples, with no indication of handling complex workflows.	The robot's task is limited to loading/unloading plates, with no intricate multi-step processes.	0.4
	Environmental Uncertainty	The robot operates in a stable, controlled environment without adapting to unexpected changes or dynamic elements.	The task environment is static and predictable.	0

	Task Variability	The robot is limited to a single task—material handling for polymer pressing—with no evidence of task variation or handling of other operations.	The system's focus is on the polymer press process, without task variation.	0.2

Supplementary Table 6 ADePT Evaluation for "Navigating phase diagram complexity to guide robotic inorganic materials synthesis"[43]

Reference	Use case	Robotic System
**Chen et al., 2024**	Automated synthesis of inorganic materials using a robotic system that optimizes precursor selection based on thermodynamic principles.	A 7-axis Franka Emika Panda arm performs automated handling of materials, integrated into the **ASTRAL platform** for powder-based ceramic synthesis.

Dimension	Metric	Justification	Paper Evidence	Score
**Adaptability and Learning**	Adaptation Efficiency	The robot performs pre-programmed tasks with no real-time adaptation; the focus of learning and adaptation is on precursor selection, not robot behavior.	The system follows pre-defined workflows with no mention of the robot adapting to new tasks.	0
	Task Generalization	The robotic system is limited to specific tasks related to material synthesis, with no evidence of task generalization to other domains.	The robot is specifically used for material synthesis, handling predefined tasks in this context.	0.2
	Real-Time Adaptability	No real-time adaptability during manipulation is indicated; the robot performs tasks according to a predefined process without dynamic changes during operation.	The Panda arm performs its tasks according to pre-programmed workflows without real-time adjustments.	0.2
	Learning Method	There is no learning method applied to the robot itself; the system's learning is applied to the precursor selection and synthesis process, not robot task performance.	Learning in the paper is focused on optimizing synthesis parameters through DFT calculations, not on robot behavior or task learning.	0
**Dexterity**	Degrees of Freedom (DOF)	The Panda arm features 7 DOF, which allows flexible manipulation tasks such as handling materials and interacting with multiple stations.	"The ASTRAL system uses a 7-axis Panda robotic arm, allowing flexible handling of samples and interaction with various synthesis stations."	0.75
	Force Control	The robot can apply controlled gentle force, but	"The Panda arm can apply controlled gentle	0.4

		there is no mention of sophisticated force control for delicate manipulation.	force". No advanced force control for varying object fragility is mentioned.	
	Grasp Variety	The robot handles a consistent set of objects (test tubes, crucibles) within a predefined process, but there is no evidence of diverse grasping capabilities.	The robot consistently handles test tubes and crucibles but does not demonstrate grasping versatility across different object shapes or sizes.	0.4
**Perception**	Sensor Variety	The robot uses force and position sensors to handle materials but lacks advanced sensory integration such as tactile or visual feedback during synthesis tasks.	"The Panda arm is equipped with force and position sensors that allow it to detect collisions and apply controlled gentle force to objects."	0.2
	Feedback Integration	Feedback from force and position sensors is used for collision detection, but there is no evidence of real-time adjustment based on environmental feedback beyond this.	The force and position sensors enable basic feedback for object handling, but no adaptive feedback integration in complex tasks is mentioned.	0.4
	Environmental Awareness	The system handles objects precisely within a controlled workspace, but there is no broader environmental mapping or adaptive awareness.	The robot interacts with static equipment and objects in a predefined workspace without additional spatial awareness.	0.2
**Task Complexity**	Number of Subtasks	The robot handles material preparation, from powder dispensing to X-ray diffraction, but each task follows a linear process without high complexity or multi-layered subtasks.	The Panda arm performs tasks like handling crucibles, but these are not deeply complex sequences.	0.4
	Environmental Uncertainty	The robot operates in a controlled lab environment with no evidence of adaptability to dynamic or unpredictable conditions.	The ASTRAL system operates in a controlled environment with predictable conditions.	0
	Task Variability	The robot handles a specific set of tasks related to material synthesis, with no indication of handling diverse tasks or task variations beyond this scope.	The robotic system is tailored to the materials synthesis process and does not exhibit task variability.	0.2

Supplementary Table 7 ADePT evaluation for "Data-science driven autonomous process optimization" [44]

Reference	Use case	Robotic System
**Christensen et al., 2021**	Autonomous process optimization in chemistry using machine learning (ML) algorithms to explore chemical reaction parameters in closed-loop robotic experimentation.	The Chemspeed SWING robotic platform executes parallel reactions, automatically dispensing reagents and collecting samples for online analysis.

Dimension	Metric	Justification	Paper Evidence	Score
**Adaptability and Learning**	Adaptation Efficiency	The robot follows pre-programmed tasks and does not adapt to new tasks; all adaptation occurs via ML for optimizing reaction parameters.	Adaptation applies only to reaction conditions through the machine learning algorithms, not to the robot's manipulation.	0
	Task Generalization	The robotic system is specifically designed for chemical reaction optimization and does not generalize to tasks outside this domain.	The system is specialized for reaction parameter optimization and does not show generalization across different types of robotic tasks.	0.2
	Real-Time Adaptability	The robot does not adapt its physical actions in real time; adaptations are made by the ML algorithm between reaction cycles based on HPLC feedback.	Real-time adaptability is focused on process parameters (reaction optimization) via ML, not the robot's physical actions.	0
	Learning Method	The robot itself does not learn or improve its own performance; learning is applied to the chemical process through ML algorithms like Phoenics and Gryffin.	The learning process optimizes reaction parameters, not the robot's behavior.	0
**Dexterity**	Degrees of Freedom (DOF)	The Chemspeed SWING platform can dispense reagents and manage parallel experiments, but it lacks a multi-axis robotic arm with complex movement capabilities.	The system uses a robotic dispense head for handling liquid volumes, but no dexterous arm is involved.	0.2
	Force Control	No force control is needed as the system is focused on accurate liquid dispensing and does not require force-sensitive manipulation.	No force control mechanisms are referenced.	0
	Grasp Variety	The robot handles reagent dispensing and reaction setup using syringes and needles but does not manipulate diverse objects	The robot manipulates liquids and reagents, but there is no object handling beyond this.	0.2

		or materials with different shapes.		
**Perception**	Sensor Variety	The robotic system uses dispensing mechanisms and HPLC-UV for chemical analysis but lacks additional sensory modalities like tactile or force sensors for real-time control.	"Chemspeed SWING... equipped with a four-needle dispense head and four 1 ml syringe pumps to enable accurate dispenses at low volumes."	0
	Feedback Integration	Feedback is integrated from HPLC analysis, but this does not directly inform the robot's actions; instead, it informs the machine learning algorithm to adjust future experiments.	"HPLC results are used by ChemOS to propose new experimental parameters for the robot."	0.2
	Environmental Awareness	The robotic system operates in a controlled lab environment, handling materials and chemicals, with no dynamic environmental interaction or spatial awareness.	The system functions within a static laboratory setting without adapting to environmental changes .	0.2
**Task Complexity**	Number of Subtasks	The robot performs sequential tasks related to reagent dispensing and sample collection, but these are well-defined and not highly complex.	The Chemspeed SWING system automates liquid handling and reaction setup, but these are relatively simple, repetitive tasks.	0.4
	Environmental Uncertainty	The robot operates in a controlled environment with no need to handle dynamic or unpredictable conditions.	The system does not face changing environments, as it is designed for a controlled laboratory setup.	0
	Task Variability	The robot handles a specific task related to chemical synthesis and reaction optimization, without demonstrating versatility across other tasks.	The system focuses solely on chemical reaction optimization, and no task variation is presented.	0.2

Supplementary Table 8 ADePT evaluation for "A robotic platform for flow synthesis of organic compounds informed by AI planning" [45]

Reference	Use case	Robotic System
**Coley et al. 2019**	Automating the synthesis of organic compounds using a robotic platform integrated with AI-driven synthesis planning.	A six-axis robotic manipulator configures flow chemistry reactors and fluidic connections for the synthesis of small organic molecules in a reconfigurable system.

Dimension	Metric	Justification	Paper Evidence	Score
**Adaptability and Learning**	Adaptation Efficiency	The robot does not learn or adapt; all adaptations are	"The system executes CRFs based on the AI-	0.2

		handled by AI planning (ASKCOS) and expert input, rather than real-time adjustments by the robot itself.	planned synthesis, with expert adjustments for process variables."	
	Task Generalization	The robot is used for configuring synthesis setups for different molecules, but its functionality is limited to this domain and does not generalize to broader tasks.	The robot's role is limited to chemical synthesis setups, with no task diversity outside this scope .	0.2
	Real-Time Adaptability	The robot does not exhibit real-time adaptability; it configures systems according to predefined CRFs and does not adjust its actions dynamically during operation.	The platform operates based on pre-programmed instructions (CRFs) without real-time adjustments.	0
	Learning Method	No learning is applied to the robot's actions; all learning pertains to the AI-driven synthesis planning (ASKCOS) for reaction optimization, not robot behavior.	"The robotic platform executes synthesis according to CRFs generated by AI planning (ASKCOS)."	0
**Dexterity**	Degrees of Freedom (DOF)	The six-axis robotic manipulator provides good flexibility for configuring reactors and fluidic lines, but less than what would be possible with a seven-axis system.	"A six-axis robotic manipulator selects process modules from storage locations and arranges them in the sequence required for a particular synthesis."	0.6
	Force Control	No force control is mentioned, indicating that the robot does not adapt its force when handling components.	The paper does not reference force-sensitive handling or any adjustment based on the type of material being manipulated.	0
	Grasp Variety	The robot handles fluid lines and reactors but does not engage in varied object manipulation; its grasping ability is limited to these specific components.	The robot's tasks are limited to manipulating process modules and fluidic connections.	0.4
**Perception**	Sensor Variety	The robotic system utilizes basic positioning sensors to assemble flow paths and fluid connections, but it does not use advanced sensors like force or vision.	"The robotic manipulator configures the synthesis apparatus by assembling the required unit operations and reagent lines on demand."	0

	Feedback Integration	Feedback is integrated into the chemical process via fluid flow monitoring and reaction conditions but does not inform the robot's physical actions in real time.	"The system follows the CRF to prime, set flow rates, set pressure and temperature, and wash and disassemble the process."	0.2
	Environmental Awareness	The robotic system operates in a controlled environment and assembles components based on predefined parameters, with no external awareness or adaptation.	The robot configures modules according to pre-defined recipes, without reacting to environmental changes.	0.2
**Task Complexity**	Number of Subtasks	The robot configures multiple reactors, separators, and fluid lines for different chemical processes, but these tasks are well-defined and not deeply complex.	The system handles the assembly of multiple unit operations for different synthesis tasks, but these are linear setups.	0.4
	Environmental Uncertainty	The robot operates in a highly controlled environment without any dynamic or unpredictable conditions.	The system works in a stable laboratory environment with no environmental uncertainties.	0
	Task Variability	The robot configures a variety of chemical synthesis pathways, but its role is confined to this domain without handling other types of tasks.	The robot is specialized for assembling flow chemistry setups, with no task variation outside of this role.	0.4

Supplementary Table 9 ADePT evaluation for "Establishing and testing a robot-based platform to enable the automated production of nanoparticles in a flexible and modular way" [46]

Reference	Use case	Robotic System
**Dembski et al. 2023**	Automated production of silica nanoparticles using a dual-arm robotic platform designed for flexible and reproducible synthesis.	A dual-arm robot performs material handling tasks such as dosing, mixing, and centrifugation in the automated synthesis of nanoparticles, with programmable logic control (PLC) for process coordination.

Dimension	Metric	Justification	Paper Evidence	Score
**Adaptability and Learning**	Adaptation Efficiency	The robot follows predefined processes for material handling and synthesis, without any learning or real-time adaptation.	"The process was broken down into specific tasks that the robot performs sequentially, with predefined job programming."	0

	Task Generalization	The robot is designed for a specific task—nanoparticle production—but the modularity of the system offers potential for adaptation to other similar tasks.	"The system is designed in a modular concept, enabling simple adaptation to different NP production processes."	0.4
	Real-Time Adaptability	The robot does not exhibit real-time adaptability; it operates according to preset instructions and job sequences, without dynamic adjustment.	"Positions of the robot and movements were programmed in jobs that can be called by a PLC."	0
	Learning Method	The robot itself does not learn from its tasks; all learning and adaptation occur in the design and setup phase, not during task execution.	"No learning method is mentioned in relation to the robot's actions."	0
**Dexterity**	Degrees of Freedom (DOF)	The dual-arm robot provides greater flexibility and reach compared to a single-arm system, allowing for complex handling tasks within the synthesis process.	"The dual-arm robot can handle all tools and materials during the synthesis and serves as a connecting link between the units inside the robot cell."	0.8
	Force Control	The robot features force control for its grippers, enabling precise manipulation of synthesis components, but no force control beyond the grippers is mentioned.	"The grippers feature force control, which allows the robot to ensure secure gripping and handle materials carefully."	0.6
	Grasp Variety	The robot uses linear electric grippers and handles a specific set of tools and containers (e.g., pipettes, centrifuge tubes), but its grasp variety is limited to these tasks.	"The robot handles tools such as pipettes, centrifuge tubes, and vessels using its two grippers."	0.4
**Perception**	Sensor Variety	The robotic system is equipped with force sensors in the grippers for secure handling but lacks more advanced sensory inputs like vision or tactile sensors.	"The robot is equipped with two linear electric grippers. The grippers feature force control, which allows to control the contact pressure and ensure secure gripping."	0.2
	Feedback Integration	Force feedback is integrated into the robot's grippers, but there is no evidence of real-time feedback from other	"The grippers feature force control, allowing to control the contact pressure and ensure secure gripping."	0.4

		parts of the system (e.g., environmental or object recognition).		
	Environmental Awareness	The robot operates in a controlled, modular cell with no broader environmental awareness or mapping capabilities.	"The dual-arm robot is installed in a modular robot cell... the robot interacts with various synthesis tools within this controlled environment."	0.2
**Task Complexity**	Number of Subtasks	The robot handles multiple complex subtasks like liquid dosing, centrifugation, and pipetting, but these are predefined and performed sequentially.	"The robot cell performs tasks like dosing, mixing, and centrifugation, broken down into smaller functional steps."	0.6
	Environmental Uncertainty	The robot operates in a stable environment with no unexpected or unpredictable changes, as it works within a highly controlled lab setting.	"The robot system operates in a controlled lab environment with predefined setups."	0
	Task Variability	The system is highly specialized for nanoparticle production but could potentially adapt to other wet-chemical synthesis processes in the future.	"The system is designed in a modular concept and can be adapted for different nanoparticle production processes."	0.4

Supplementary Table 10 ADePT evaluation for ExpFlow: a graphical user interface for automated reproducible electrochemistry [47]

Reference	Use case	Robotic System
**Duke et al., 2024**	Automating cyclic voltammetry (CV) experiments using a robotic platform controlled through the ExpFlow software for electrochemical data collection and reproducibility.	A Kinova Gen 3 robotic arm with six degrees of freedom (DOF) is used to automate the manipulation of solution vials in CV experiments.

Dimension	Metric	Justification	Paper Evidence	Score
**Adaptability and Learning**	Adaptation Efficiency	The robot follows predefined workflows, with no real-time adaptation to process changes or environmental factors.	"The robotic experiments were run from predefined workflows based on ExpFlow templates."	0
	Task Generalization	The robotic system is designed specifically for CV experiments, with no evidence of generalization beyond this particular task domain.	The system is used solely for cyclic voltammetry tasks.	0.2

	Real-Time Adaptability	The robot does not demonstrate real-time adaptability, as it follows predefined workflows without dynamic changes during execution.	The workflow is predefined and not subject to real-time modifications.	0
	Learning Method	There is no evidence of learning or improvement in the robot's actions; the adaptation happens through human input in the template setup phase, not through autonomous robot learning.	No learning is applied to the robot itself; only the workflow process can be adjusted by users.	0
**Dexterity**	Degrees of Freedom (DOF)	The six-DOF robotic arm allows for sufficient flexibility to handle vials and conduct the transfer required for CV experiments, though less than a 7-DOF system.	"A Kinova Gen 3 robotic arm with six degrees of freedom was used."	0.6
	Force Control	There is no indication of force control in the robotic gripper, which may limit the handling of delicate materials.	No force-sensitive control is mentioned in the description of the robotic platform.	0
	Grasp Variety	The robot handles standardized vials and equipment, but its ability to grasp and manipulate different object types appears limited.	The robotic arm is used primarily for transferring solution vials, without evidence of handling more varied shapes or sizes of objects.	0.2
**Perception**	Sensor Variety	The system relies on positioning sensors for vial placement but lacks advanced sensors like force or vision to aid in manipulation.	"A six-axis Kinova Gen 3 robotic arm was used to transfer solution vials to and from the potentiostat system."	0
	Feedback Integration	There is no real-time sensor feedback that directly informs the robot's physical actions during manipulation.	"Once a researcher creates an ExpFlow template and converts it into a robotic workflow... the robotics API translates the loaded workflow into robotic actions."	0.2
	Environmental Awareness	The robot operates in a predefined and controlled environment without dynamic interaction or external environmental awareness.	The robot is programmed to interact with specific vials and equipment, with no adaptation to external changes.	0.2

**Task Complexity**	Number of Subtasks	The robot handles multiple subtasks such as vial transfer and electrode placement, but the complexity remains low as these tasks are standardized.	"The workflow includes tasks such as transferring vials to the potentiostat and initiating CV measurements."	0.4
	Environmental Uncertainty	The robot operates in a controlled lab setting with no environmental variability or need for adaptation.	The environment is static and controlled with no mention of unexpected variables.	0
	Task Variability	The robotic system is limited to electrochemical experiments, with no demonstrated ability to handle other tasks or variable operations beyond this scope.	The system is specifically designed for electrochemistry experiments and does not show variability beyond this application.	0.2

Supplementary Table 11 ADePT evaluation for "Dual-arm Robotic Compound-oriented Measurement System: Integration of a Positive Pressure Solid Phase Extraction Unit" [48]

**Reference**	**Use case**	**Robotic System**
**Fleischer et al., 2021**	Automating the sample preparation process in a measurement system using dual-arm robotics integrated with a solid phase extraction (SPE) unit.	A dual-arm robotic platform performs tasks such as handling, transferring, and preparing chemical compounds in an integrated positive pressure solid-phase extraction (SPE) system.

**Dimension**	**Metric**	**Justification**	**Paper Evidence**	**Score**
**Adaptability and Learning**	Adaptation Efficiency	The robot follows predefined instructions with no evidence of learning or real-time adaptation to task changes or external inputs.	"All tasks are pre-programmed with no dynamic adaptation during the process."	0
	Task Generalization	The robot is highly specialized for the solid phase extraction task, and there is no evidence of generalizing to other domains or tasks.	"The system is built around the SPE unit, without generalization to other processes."	0.2
	Real-Time Adaptability	The system lacks real-time adaptability, operating according to pre-programmed paths without adjusting to real-time conditions or changes in the task environment.	"Robotic tasks are performed according to predefined paths synchronized with the SPE unit's timing."	0
	Learning Method	No learning methods are applied to the robotic system, as all tasks are defined and executed	"No learning capabilities are integrated into the robot's operation."	0

		without autonomous refinement or improvement.		
**Dexterity**	Degrees of Freedom (DOF)	The dual-arm system provides increased flexibility for manipulating multiple components within the system, handling samples with higher dexterity than single-arm setups.	"The dual-arm setup allows handling of sample containers and precise alignment with the SPE unit simultaneously."	0.8
	Force Control	No force control is mentioned, meaning the robot handles objects without adjusting its grip dynamically based on object properties or fragility.	"The system operates with predefined movements, lacking adaptive force feedback."	0
	Grasp Variety	The robot can handle specific types of objects (e.g., sample containers), but there is no evidence of diverse object manipulation beyond this.	"Robotic arms are used to manipulate vials and containers for SPE processing."	0.4
**Perception**	Sensor Variety	The robotic system employs basic positioning sensors for arm coordination but lacks advanced sensory modalities like vision or force feedback.	"The robot arms are synchronized through pre-programmed pathways for accurate placement of samples in the SPE unit."	0
	Feedback Integration	There is no real-time feedback informing the robot's physical manipulation of objects, and actions are pre-programmed without adaptive feedback loops.	"All robot movements are pre-programmed to ensure synchronization with the SPE unit's operation."	0
	Environmental Awareness	The robot operates within a controlled, fixed environment and does not exhibit awareness of or adaptation to changes in its surroundings.	The system's environment is entirely predefined, with no environmental interaction beyond its programmed workspace.	0.2
**Task Complexity**	Number of Subtasks	The system performs a series of predefined tasks, including transferring, handling, and positioning, but these are linear and relatively simple.	"The dual-arm robot system is responsible for handling multiple samples and placing them in the SPE unit for processing."	0.4
	Environmental Uncertainty	The system operates in a controlled environment with no environmental variability or uncertainty, handling fixed workflows.	"The system operates in a static laboratory setup."	0

	Task Variability	The robot's task variability is limited to the SPE process, and there is no evidence of its ability to handle different tasks or workflows beyond this scope.	"The dual-arm robot is focused on handling tasks related to solid-phase extraction."	0.2

Supplementary Table 12 ADePT evaluation for " A Bayesian experimental autonomous researcher for mechanical design" [49]

Reference	Use case	Robotic System
**Gongora et al., 2020**	A robotic system used in conjunction with Bayesian optimization to autonomously conduct additive manufacturing (AM) experiments, testing and optimizing mechanical properties like toughness.	The system integrates a UR5e six-axis robotic arm to automate the handling of 3D-printed parts between a set of five printers and testing equipment, performing tasks such as retrieval, weighing, and mechanical testing.

Dimension	Metric	Justification	Paper Evidence	Score
**Adaptability and Learning**	Adaptation Efficiency	The robot performs predefined workflows; adaptation and learning are applied to the experimental process (Bayesian optimization), not the robot's actions.	"Learning is focused on experiment selection and optimization, rather than robotic task adaptation."	0
	Task Generalization	The robot is highly specialized for AM and mechanical testing, with no generalization to tasks outside this domain.	The robot is dedicated to retrieving printed parts and performing specific tests; it is not generalized to other types of tasks.	0.2
	Real-Time Adaptability	There is no real-time adaptability in the robot's physical actions, as it follows a preprogrammed sequence of steps during the experiments.	"Robotic movements are predefined and not subject to dynamic adjustments based on real-time conditions."	0
	Learning Method	The robot itself does not learn; the Bayesian optimization process informs which experiments to perform, but this does not alter how the robot executes its tasks.	"The Bayesian framework selects experiments, but the robot follows fixed tasks and does not learn from its actions."	0
**Dexterity**	Degrees of Freedom (DOF)	The six-axis robotic arm provides sufficient flexibility for object handling tasks within its operating range, but lacks the dexterity of seven-axis systems.	"A six-axis UR5e robotic arm retrieves, weighs, and tests parts in an automated system."	0.6

	Force Control	There is no mention of force-sensitive handling, meaning the robot likely follows fixed movement patterns without adjusting based on the fragility or weight of objects.	No force control is described in the handling of printed parts. The robot's tasks are preprogrammed.	0
	Grasp Variety	The robot primarily handles printed parts and standardized tools such as vials and containers; its grasp variety is therefore limited to specific object types.	"The robotic arm handles parts between printers and testing stations but lacks the versatility to handle diverse object types."	0.2
**Perception**	Sensor Variety	The robotic system utilizes basic positioning sensors but lacks additional sensory inputs such as tactile feedback or vision systems for object recognition.	"The system uses a UR5e robotic arm for automated handling between 3D printers and testing equipment."	0
	Feedback Integration	There is no mention of real-time feedback from sensors directly influencing the robot's actions; the workflow is predefined, focusing on mechanical testing and handling.	"Robotic actions are coordinated with a testing machine and scale for mechanical testing but do not use real-time feedback during manipulation."	0.2
	Environmental Awareness	The robot operates in a controlled, fixed environment with no broader environmental awareness or adaptability.	"The robotic arm is used within a predefined setup involving 3D printers and testing machines."	0.2
**Task Complexity**	Number of Subtasks	The robot handles multiple predefined tasks such as transferring, weighing, and testing parts, but these are linear and not particularly complex.	"The system integrates 3D printing, weighing, and mechanical testing, with robotic handling in between."	0.4
	Environmental Uncertainty	The system operates in a highly controlled laboratory setting with no environmental uncertainty or adaptation required.	"The robot functions in a predefined lab environment, without environmental changes."	0
	Task Variability	The system is specialized for a narrow range of tasks—handling 3D-printed parts and conducting mechanical tests—without variability in other types of operations.	"The robot performs only handling tasks related to 3D printing and mechanical testing."	0.2

Supplementary Table 13 ADePT evaluation for "Core Processes in Intelligent Robotic Lab Assistants: Flexible Liquid Handling" [50]

Reference	Use case	Robotic System
**Knobbe et al., 2022**	Automating liquid handling tasks in laboratories using an intelligent robotic system capable of pipetting with high precision, following ISO 8655 standards.	The system features a Franka Emika 7-DOF robotic arm integrated into a gantry system for flexible, force-sensitive liquid handling operations, such as pipetting and handling non-standard labware.

Dimension	Metric	Justification	Paper Evidence	Score
**Adaptability and Learning**	Adaptation Efficiency	The robot follows pre-programmed tasks and uses predefined skills for liquid handling, with no evidence of real-time learning or adaptation.	"The robot operates based on a predefined skill taxonomy, following programmed protocols."	0
	Task Generalization	The system is designed to handle a variety of pipetting tasks, but its scope is confined to lab-based operations without generalization beyond liquid handling.	"The system is specialized for flexible pipetting and liquid handling, but does not generalize to tasks outside this domain."	0.4
	Real-Time Adaptability	The system does not exhibit real-time adaptability to task changes; it follows predefined workflows without adjusting dynamically during operation.	"All processes are predefined and executed according to protocols without dynamic real-time changes."	0
	Learning Method	There is no learning method applied to the robot itself; the system operates based on predefined tasks and does not improve or adapt through learning.	"No learning or adaptive processes are integrated into the robot's workflow."	0
**Dexterity**	Degrees of Freedom (DOF)	The 7-DOF robotic arm offers high flexibility, allowing precise manipulation of labware in confined spaces such as biosafety cabinets.	"The robotic system has 7-DOF with torque sensing for flexible and precise manipulation."	0.8
	Force Control	The system has advanced force control, particularly for tasks like gripping pipettes and pressing buttons, enabling precise pipetting and handling.	"Force-sensitive manipulation ensures tight pipette tip fitting and handling of non-standard containers."	0.8
	Grasp Variety	The robot handles pipettes and labware with interchangeable grippers, offering flexibility for standard lab tasks but limited to lab equipment.	"The system uses a Finger Quick-Exchange System, allowing for flexible handling of various lab tools."	0.6

**Perception**	Sensor Variety	The robotic system uses a range of sensors, including force sensors in the robotic arm joints, for precise manipulation, but lacks vision systems for object recognition.	"Link-side torque sensing in each joint provides torque control and fast collision detection for safe operation."	0.2
	Feedback Integration	The robot uses force feedback for pipetting tasks, allowing precise control over liquid handling, but lacks broader environmental feedback systems.	"The system includes force-sensitive manipulation, using feedback for tasks like pipetting."	0.6
	Environmental Awareness	The robot operates within a controlled workspace and does not exhibit adaptive environmental awareness beyond the pipetting task.	"The system relies on predefined locations for labware, using markers for object identification and pose detection."	0.2
**Task Complexity**	Number of Subtasks	The robot handles multiple subtasks related to liquid handling, including pipette tip pickup, aspiration, and dispensing, but these tasks are well-defined and linear.	"The system handles pipetting tasks, including tip pickup, liquid transfer, and pipette cleaning."	0.6
	Environmental Uncertainty	The robot operates in a controlled laboratory environment with no adaptation to environmental uncertainty or unpredictable conditions.	"The system works in a stable, controlled environment without environmental variability."	0
	Task Variability	The robotic system focuses on a narrow range of tasks (pipetting and liquid handling) but is designed to handle different labware and flexible volumes.	"The system can flexibly handle pipetting in various containers and different liquid volumes."	0.4

Supplementary Table 14 ADePT evaluation for "Development of a Robotic System for Automatic Organic Chemistry Synthesis" [51]

**Reference**	**Use case**	**Robotic System**
**Lim et al., 2021**	Automating the synthesis of organic compounds using a robotic arm for tasks such as liquid handling, mixing, and filtration.	A Denso VS-060 six-axis robotic arm is used in combination with a custom Robotiq Hand-E gripper and a wireless syringe pump to perform complex chemical reactions without modifying existing lab equipment.

**Dimension**	**Metric**	**Justification**	**Paper Evidence**	**Score**
**Adaptability and Learning**	Adaptation Efficiency	The robot follows predefined workflows and does not learn or adapt its behaviour in real	"No real-time adaptation or learning is implemented in the robotic system."	0

		time based on environmental conditions or task variations.		
	Task Generalization	The system is focused on organic chemistry synthesis and is not generalized to handle other lab tasks or applications beyond this domain.	"The robot is specialized for liquid handling and synthesis tasks specific to organic chemistry."	0.2
	Real-Time Adaptability	The robotic arm performs tasks in a preprogrammed sequence without adjusting dynamically to changes during task execution.	"The system operates based on a set sequence without real-time adaptability."	0
	Learning Method	There is no learning applied to the robotic system itself; all processes are predefined and do not evolve based on task performance.	"No learning or optimization of robotic actions is integrated into the system."	0
**Dexterity**	Degrees of Freedom (DOF)	The six-axis robotic arm provides sufficient flexibility for complex liquid handling tasks, though it is less versatile than a seven-axis system.	"The Denso VS-060 six-axis arm provides flexibility for liquid handling and reaction setup."	0.6
	Force Control	There is no mention of force-sensitive manipulation, and handling is likely based on predefined movements without dynamic force adjustments.	"The robot lacks advanced force control, limiting its ability to handle delicate objects dynamically."	0
	Grasp Variety	The system can handle specific labware and vials using the Robotiq Hand-E gripper, but its grasping capabilities are limited to these objects.	"The gripper handles syringes and vials but does not demonstrate versatility for a wider range of objects."	0.4
**Perception**	Sensor Variety	The robotic system relies on positioning sensors to control the arm's movement, but no additional sensors like force or vision systems are integrated.	"The system includes a Denso VS-060 robotic arm with basic positioning sensors, but lacks additional sensory inputs."	0
	Feedback Integration	There is no real-time feedback for dynamic adjustments in the robot's actions, though task completion (e.g., liquid transfer) is monitored indirectly.	"The syringe pump and robot arm are synchronized but there is no adaptive feedback from sensors during operation."	0.2
	Environmental Awareness	The robot operates in a structured, controlled lab environment and lacks	"The system performs tasks in a predefined lab environment with no	0.2

		adaptability to environmental changes or awareness beyond predefined setups.	adaptability to dynamic changes."	
**Task Complexity**	Number of Subtasks	The robot handles multiple steps in the reaction process, such as liquid transfer, mixing, and filtration, but the tasks follow a simple, linear structure.	"The robot performs tasks like liquid handling, stirring, and filtration sequentially as part of the synthesis."	0.6
	Environmental Uncertainty	The system operates in a stable lab environment and does not handle any unpredictable or dynamic environmental conditions.	"The robot works in a controlled, predictable environment."	0
	Task Variability	The system is specifically designed for chemical synthesis and does not handle tasks beyond organic chemistry workflows.	"The robot is specialized for organic chemistry reactions and does not demonstrate versatility beyond these tasks."	0.2

Supplementary Table 15 ADePT evaluation for "Self-driving laboratory for accelerated discovery of thin-film materials"[52]

**Reference**	**Use case**	**Robotic System**
**MacLeod et al., 2020**	Automating the discovery and optimization of thin-film materials for energy applications using a self-driving laboratory equipped with robotic systems and Bayesian optimization algorithms.	The platform, called "Ada," uses a modular polar robotic arm with a pneumatic gripper to perform tasks like aspirating, dispensing, spin-coating, and characterizing thin films.

**Dimension**	Metric	Justification	Paper Evidence	Score
**Adaptability and Learning**	Adaptation Efficiency	Adaptation occurs at the experimental level (Bayesian optimization), not in the robot's handling or manipulation of materials. The robot follows predefined workflows.	"Experimental design is adapted iteratively by a Bayesian optimization algorithm, but the robotic actions are fixed."	0
	Task Generalization	The robotic system is specialized for thin-film synthesis and characterization, with no evidence of generalization to tasks outside of this domain.	"The platform is specifically designed for optimizing thin films for solar cells and other energy applications."	0.2
	Real-Time Adaptability	The robot performs predefined sequences without real-time adaptation or learning from its environment.	"The experiments are pre-programmed, and no real-time adaptation occurs in the robotic processes."	0
	Learning Method	Learning occurs in the experiment selection via the	"Ada uses a Bayesian optimization algorithm to	0

		Phoenics Bayesian optimization algorithm, not in the robotic control or manipulation itself.	iteratively design experiments based on previous data."	
**Dexterity**	Degrees of Freedom (DOF)	The 4-DOF robotic arm is enhanced with a rotating joint at the end effector, allowing for greater dexterity in capping/uncapping and liquid dispensing tasks.	"The system uses a polar robot with 4-DOF and a rotating joint for vial handling and dispensing."	0.7
	Force Control	There is no evidence of force control, limiting the robot's handling of delicate materials or tasks requiring nuanced grip adjustments.	No force-sensitive manipulation is mentioned for the gripper or other robotic components.	0
	Grasp Variety	The robot handles standardized tools like pipettes and vials but is not designed for a wide range of objects beyond laboratory-specific materials.	"The robot uses a pneumatic gripper to handle vials and other standardized lab equipment."	0.4
**Perception**	Sensor Variety	The robotic system includes basic sensors for position control but does not use advanced sensors like vision or force feedback for real-time adjustments.	"The robot is equipped with a rotatable, pneumatic gripper and a pipette mount for liquid handling tasks."	0
	Feedback Integration	There is no feedback used for dynamic adjustment of the robot's actions, although task completion is monitored through external devices (e.g., spectroscopy).	"Feedback systems are limited to optical and conductivity measurements, not to robotic manipulation itself."	0.2
	Environmental Awareness	The robot operates in a controlled, structured environment without broader environmental awareness or adaptability.	"The robot operates in a predefined setup interacting with experimental modules like spin coaters and spectrometers."	0.2
**Task Complexity**	Number of Subtasks	The robot handles multiple subtasks, including aspirating, dispensing, spin-coating, annealing, and characterizing thin films, though these tasks are linear.	"The platform autonomously handles thin-film synthesis, spin coating, annealing, and spectroscopic characterization."	0.6
	Environmental Uncertainty	The system operates in a highly controlled lab environment without dynamic or unpredictable changes that require adaptation.	"The robot works in a structured, controlled lab setup designed for thin-film optimization."	0

	Task Variability	The system is designed specifically for thin-film discovery and optimization, with little variability in the tasks it can perform beyond this scope.	"The system is modular and flexible for different thin-film workflows but does not handle tasks outside material synthesis and characterization."	0.2

Supplementary Table 16 ADePT evaluation for "A self-driving laboratory advances the Pareto front for material properties"[53]

Reference	Use case	Robotic System
**MacLeod et al., 2022**	Automating the discovery and optimization of material properties (e.g., conductivity vs. processing temperature) using a self-driving lab equipped with robotic systems and Bayesian optimization algorithms.	The self-driving laboratory (Ada) employs two robots: a 4-axis N9 robot for liquid handling and thin-film deposition, and a 6-axis UR5e robotic arm for sample transfer and interfacing with analytical equipment (XRF microscope). The robots automate the creation and characterization of palladium thin films through combustion synthesis.

Dimension	Metric	Justification	Paper Evidence	Score
**Adaptability and Learning**	Adaptation Efficiency	Adaptation occurs at the experimental level (Bayesian optimization), not in the robot's physical manipulation of materials.	"The qEHVI algorithm adapts the experimental plan to optimize material properties, but the robot follows fixed instructions."	0
	Task Generalization	The system is specialized for thin-film synthesis and characterization, with no evidence of generalization to broader laboratory tasks.	"The self-driving lab is designed for thin-film synthesis through combustion methods."	0.2
	Real-Time Adaptability	The robots follow predefined tasks without real-time adaptability or changes in response to dynamic conditions.	"The robots execute fixed workflows for sample preparation and analysis."	0
	Learning Method	Learning is applied to experiment selection via the Bayesian optimization algorithm (qEHVI), but not to the robot's handling or manipulation capabilities.	"The self-driving lab uses the qEHVI algorithm to optimize experiments, but the robots themselves do not learn from their actions."	0
**Dexterity**	Degrees of Freedom (DOF)	The combination of a 4-axis N9 robot and a 6-axis UR5e robotic arm provides a high degree of flexibility for handling, deposition, and characterization tasks.	"The N9 is a 4-axis robot, while the UR5e is a 6-axis collaborative robot, enabling complex handling tasks for sample transfer and analysis."	0.7
	Force Control	There is no mention of force-sensitive manipulation in the system, which may limit its	No force control is described in the manipulation of samples	0

		ability to handle delicate objects with precision.	during thin-film deposition or analysis.	
	Grasp Variety	The robotic system handles standardized labware such as vials, glass slides, and thin films, but does not demonstrate versatility beyond these objects.	"The robots handle glass slides and other consumables for thin-film preparation and analysis."	0.4
**Perception**	Sensor Variety	The system includes basic sensors for positioning and pipetting, but there is no evidence of advanced sensors such as vision or force feedback for object recognition or manipulation.	"The N9 robot was equipped to mix, drop cast, and anneal thin films, while the UR5e arm transported samples to the XRF microscope for analysis."	0
	Feedback Integration	Feedback from gravimetric monitoring helps track task outcomes, but there is no real-time feedback for dynamically influencing the robot's manipulation.	"Pipetting errors were minimized through gravimetric feedback, but no dynamic feedback during robotic actions is mentioned."	0.2
	Environmental Awareness	The robots operate in a structured, fixed environment and have no capacity for real-time environmental awareness or adaptation.	"The robots function within a highly controlled environment designed for thin-film synthesis and characterization."	0.2
**Task Complexity**	Number of Subtasks	The robot performs multiple subtasks, such as pipetting, drop casting, annealing, and XRF analysis, but these tasks follow a simple, linear sequence.	"The experimental workflow includes multiple steps, from precursor mixing to thin-film characterization, executed sequentially by the robots."	0.6
	Environmental Uncertainty	The system operates in a stable, controlled lab environment without any environmental variability.	"The lab setup is entirely predefined, with no unexpected changes in the environment."	0
	Task Variability	The system focuses on a narrow range of tasks related to thin-film synthesis, with no ability to handle broader workflows or other types of experiments.	"The robot system is specifically designed for palladium thin-film synthesis and analysis."	0.2

Supplementary Table 17 ADePT evaluation for "Bayesian Optimization of Computer-Proposed Multistep Synthetic Routes on an Automated Robotic Flow Platform" [54]

Reference	Use case	Robotic System
**Nambiar et al., 2022**	Automating the optimization of multistep synthetic routes for organic chemistry on a	The platform uses a 4-axis gantry robot with a rotary joint for module placement and reconfiguration in the continuous flow synthesis

	continuous flow platform using Bayesian optimization.	system. It manages liquid handling and interfaces with process analytical tools.

Dimension	Metric	Justification	Paper Evidence	Score
**Adaptability and Learning**	Adaptation Efficiency	The robot follows pre-programmed tasks, while the learning (Bayesian optimization) is applied to experimental conditions and chemical processes, not the robot's handling.	"Bayesian optimization helps optimize reaction conditions, but the robot executes fixed reconfiguration tasks."	0
	Task Generalization	The robotic system is focused on handling flow synthesis processes and does not generalize to broader laboratory tasks.	"The platform is specialized for flow synthesis and reaction optimization using the gantry robot."	0.2
	Real-Time Adaptability	The robot does not exhibit real-time adaptability in its movements, as it executes predefined actions in response to optimized conditions for the chemical process.	"The robot reconfigures modules based on predefined instructions and does not adapt in real-time to external conditions."	0
	Learning Method	Learning is applied to optimize chemical reaction conditions, not the robot’s operation or actions. The Bayesian optimization algorithm focuses on process conditions.	"The Bayesian algorithm optimizes reaction variables like temperature and residence time, but the robot follows fixed task sequences."	0
**Dexterity**	Degrees of Freedom (DOF)	The 4-axis gantry robot with a rotary joint offers moderate dexterity, allowing the robot to place modules in different process bays and handle modular reconfigurations.	"The gantry robot has 3 linear XYZ Cartesian axes and 1 rotary axis (θ) for flexible process reconfiguration."	0.65
	Force Control	No force-sensitive manipulation is mentioned, indicating limited ability to handle fragile materials or perform fine adjustments based on force.	"The robot executes pick-and-place operations but does not have force-sensitive capabilities."	0
	Grasp Variety	The system is designed to manipulate standard labware and process modules, but its gripper is not specialized for diverse object handling.	"The gantry robot manages process modules like reactors, separators, and analytical equipment but is not designed for handling a wide range of objects."	0.4
**Perception**	Sensor Variety	The system uses basic sensors to control the gantry arm’s positioning and fluid	"The platform consists of a 4-axis gantry robot... Process modules include	0

		flow but lacks vision or tactile sensors for adaptive handling.	inline FT-IR and LC-MS for real-time monitoring of reactions."	
	Feedback Integration	The robotic system relies on feedback from process analytical technology (PAT) like FT-IR and LC-MS for real-time monitoring, but this feedback does not directly control the robot's movements.	"Inline FT-IR and LC-MS provide real-time feedback on reaction progress."	0.4
	Environmental Awareness	The robot operates in a controlled laboratory environment and lacks environmental sensors for adapting to dynamic conditions.	"The robot reconfigures process modules within a predefined environment for continuous flow synthesis."	0.2
**Task Complexity**	Number of Subtasks	The robot handles multiple subtasks in multistep flow synthesis, such as placing modules, managing fluid flow, and sampling reactions for analysis.	"The system executes multistep synthetic routes, including reaction, separation, and analysis, by coordinating modular components."	0.6
	Environmental Uncertainty	The system operates in a stable, controlled lab setup with no variability in environmental conditions.	"The robotic platform functions within a controlled, predictable environment for flow synthesis."	0
	Task Variability	The system is focused on modular, flow-based synthetic routes, and its tasks do not vary significantly outside of this domain.	"The robotic platform reconfigures flow synthesis components but does not extend to other laboratory processes."	0.2

Supplementary Table 18 ADePT evaluation for "Robotic pendant drop: containerless liquid for μs-resolved, AI-executable XPCS"[55]

Reference	Use case	Robotic System
**Ozgulbas et al., 2023**	Automating liquid sample preparation and data acquisition for X-ray Photon Correlation Spectroscopy (XPCS) using a robotic system integrated with a pendant drop method.	The system uses a UR3e robotic arm with an electronic pipette to generate and manipulate pendant drops for automated liquid handling, combining this with XPCS measurements.

Dimension	Metric	Justification	Paper Evidence	Score
**Adaptability and Learning**	Adaptation Efficiency	The robot follows preprogrammed tasks with no evidence of adaptive behavior or real-time learning based on	"The robotic system operates according to fixed workflows and does not adapt based on real-time feedback."	0

		environmental or task variations.		
	Task Generalization	The robotic system is specialized for handling liquid samples and performing XPCS measurements, with no evidence of generalization to broader laboratory tasks.	"The robot is focused on automating XPCS measurements with liquid handling, specifically for pendant drops."	0.2
	Real-Time Adaptability	The robot does not exhibit real-time adaptability; it executes a sequence of preplanned actions without adjustment during execution.	"All robot actions are predefined, with no adaptability during the experiment."	0
	Learning Method	Learning is applied to experimental design through AI-executable protocols but not to the robot's own operation or movement.	"The robot executes predefined steps without learning or adapting its handling behaviour."	0
**Dexterity**	Degrees of Freedom (DOF)	The UR3e robotic arm with 6 DOF offers flexibility for manipulating liquid samples in tight spaces like beamlines, although it is limited by predefined tasks.	"The UR3e arm is used for handling the pipette and generating the pendant drop in a confined beamline environment."	0.6
	Force Control	There is no mention of force control in the robotic arm, and precise force adjustments for delicate manipulation are not needed in the experimental context.	No force-sensitive manipulation is described in handling liquid drops.	0
	Grasp Variety	The robot can manipulate pipette tips and various liquid containers, but its grasping capabilities are limited to liquid handling equipment.	"The system uses tool changers to handle pipette tips, disposable pipettes, and other liquid handling tools."	0.4
**Perception**	Sensor Variety	The system relies on positioning sensors and a 45-degree reflective mirror to ensure the proper alignment of the pendant drop with the X-ray beam.	"A 45° reflective mirror and an optical microscope monitor the pendant drop during alignment and XPCS measurements."	0.1
	Feedback Integration	The robot follows predefined workflows, and feedback is used to monitor alignment and drop stability, but not for real-time control of robotic movements.	"The drop's optical appearance is monitored during alignment, but the robot executes fixed motions without real-time	0.2

			feedback influencing its actions."	
	Environmental Awareness	The robot operates in a controlled environment but is enclosed by a polycarbonate sleeve to suppress airflow and evaporation, limiting its awareness to predefined factors.	"The pendant drop is shielded by a polycarbonate sleeve to reduce airflow and evaporation."	0.2
**Task Complexity**	Number of Subtasks	The robot handles multiple subtasks, including liquid preparation, drop generation, and alignment for XPCS measurements, but the tasks follow a simple workflow.	"The robot performs sample preparation, drop dispensing, and alignment for XPCS in a stepwise manner."	0.6
	Environmental Uncertainty	The robot operates in a stable, controlled environment without variability or adaptation to changing conditions.	"The setup is designed to limit external interference, like evaporation and airflow, using a polycarbonate enclosure."	0
	Task Variability	The system is specialized for XPCS experiments and does not handle other tasks beyond liquid handling for pendant drop generation and measurement.	"The robot is designed specifically for liquid handling and XPCS, with no evidence of versatility beyond this domain."	0.2

Supplementary Table 19 ADePT evaluation for "Automated solubility screening platform using computer vision"[56]

Reference	Use case	Robotic System
**Shiri et al., 2021**	Automating solubility screening for organic compounds using a modular platform with integrated solid and liquid dosing, computer vision, and real-time feedback to determine solubility.	The platform uses an N9 SCARA robot (Selective Compliance Articulated Robot Arm) equipped with a rotatable gripper and a probe for liquid and solid handling tasks in a solubility screening workflow.

Dimension	Metric	Justification	Paper Evidence	Score
**Adaptability and Learning**	Adaptation Efficiency	The robot follows a pre-programmed sequence with no evidence of real-time adaptation during the task execution.	"The workflow follows a fixed sequence controlled by a Python master script."	0
	Task Generalization	The system is specialized for solubility screening tasks and does not generalize to other laboratory tasks.	"The system automates solubility screening, and though modular, it is focused on this workflow."	0.2
	Real-Time Adaptability	The system lacks real-time adaptability; the robotic arm	"All robot actions follow predefined instructions	0

		does not adjust its actions dynamically during the experiments.	without real-time adaptability."	
	Learning Method	Learning is applied only to the experiment’s process (through feedback on turbidity), not to the robot's physical operations.	"The system automates the solubility process, but the robot follows fixed steps without learning."	0
**Dexterity**	Degrees of Freedom (DOF)	The N9 SCARA robot has 4 degrees of freedom (DOF) with a rotatable gripper, providing enhanced flexibility for its specific liquid handling and vial manipulation tasks.	"The N9 arm can move in three dimensions and uses a gripper and probe to handle vials and needles."	0.7
	Force Control	There is no evidence of force control, limiting the robot's ability to handle delicate objects or perform sensitive tasks.	"The robot executes fixed manipulation tasks like uncapping vials without mention of force sensitivity."	0
	Grasp Variety	The system can handle vials and liquid handling tools, but the gripper is specialized for these specific objects, limiting its versatility.	"The gripper handles HPLC vials and needles required for the solubility workflow."	0.4
**Perception**	Sensor Variety	The system relies on computer vision and image processing to monitor turbidity, with basic sensors for robot positioning. There is no advanced sensor variety.	"The HeinSight turbidity code reads images captured by a webcam to measure the turbidity of the solution and determine solubility."	0.1
	Feedback Integration	Computer vision provides feedback on turbidity, but this feedback informs the experiment's progress rather than real-time control of the robot’s actions.	"The system uses a webcam to monitor turbidity... and add solvent until the solution is fully dissolved."	0.2
	Environmental Awareness	The robot operates in a controlled laboratory setting with no broader environmental awareness or dynamic adaptation.	"The system runs solubility experiments in a controlled environment, with no indication of real-time environmental awareness."	0.2
**Task Complexity**	Number of Subtasks	The robot handles multiple subtasks, including solid and liquid dosing, vial capping/uncapping, and turbidity monitoring, but the tasks are linear and predefined.	"The workflow includes vial handling, dosing, and monitoring steps."	0.6
	Environmental Uncertainty	The system operates in a controlled environment with no	"The platform operates under predefined	0

		dynamic changes, so it does not address environmental uncertainty.	conditions without any environmental variability."	
	Task Variability	The system focuses on solubility screening, limiting task variability beyond this domain.	"The system is modular, but its focus is on automating solubility testing workflows."	0.2

Supplementary Table 20 ADePT evaluation for "Automated self-optimization, intensification, and scale-up of photocatalysis in flow" [57]

Reference	Use case	Robotic System
**Slattery et al., 2024**	Automation of photocatalysis optimization, intensification, and scale-up using a continuous-flow robotic platform with Bayesian optimization.	The RoboChem platform integrates a liquid handler, syringe pumps, a continuous-flow photoreactor, and in-line NMR. It does not include any articulated robotic arms (e.g., 6-axis arms).

Dimension	Metric	Justification	Paper Evidence	Score
**Adaptability and Learning**	Adaptation Efficiency	The system applies Bayesian optimization to adapt and optimize the reaction conditions, but not the robot's handling tasks.	"The optimization algorithm refines reaction conditions based on yield and throughput, but the robot itself does not adapt during physical tasks."	0
	Task Generalization	The platform is specifically designed for photocatalytic reaction optimization and does not generalize to other laboratory workflows.	"The RoboChem platform is specialized for optimizing photocatalytic reactions."	0.2
	Real-Time Adaptability	The system follows predefined workflows and does not dynamically adapt its actions in real time.	"The robot executes tasks sequentially without real-time adaptation."	0
	Learning Method	Learning is applied to reaction optimization (via Bayesian optimization), but not to the robot's physical operation.	"Bayesian optimization improves reaction outcomes, but there is no learning in robotic handling."	0
**Dexterity**	Degrees of Freedom (DOF)	The system relies on a liquid handler and syringe pumps for sample transfer, with no articulated robotic arms. This limits dexterity to the liquid handling domain.	"The RoboChem platform uses a liquid handler and syringe pumps to manage reactions."	0.2
	Force Control	The platform does not involve tasks requiring force-sensitive manipulation or delicate object handling.	No mention of force control for the liquid handler and syringe pumps.	0
	Grasp Variety	The liquid handler is designed specifically for reaction setups involving	"The system's liquid handler and pumps are	0.3

		liquids, with no versatility beyond these objects.	specialized for handling reaction mixtures."	
**Perception**	Sensor Variety	The system incorporates basic sensors like phase sensors to monitor reaction slugs and in-line NMR for yield analysis, but no advanced sensors like vision or tactile sensors are present.	"RoboChem uses phase sensors and in-line benchtop NMR for reaction tracking and yield analysis."	0
	Feedback Integration	Feedback is obtained from NMR analysis, which informs the optimization algorithm (Bayesian optimization), but no direct feedback adjusts the robotic system's physical actions in real-time.	"The results are fed back to the machine learning model, which recommends the next experiment, but the robot executes predefined tasks."	0.2
	Environmental Awareness	The system operates in a controlled environment, focused on flow photocatalysis, with no real-time adaptability to changes in the external environment.	"The RoboChem platform operates in a controlled benchtop environment designed for photocatalysis."	0.2
**Task Complexity**	Number of Subtasks	The robot handles multiple subtasks, such as liquid handling, reaction monitoring, and NMR analysis, but they follow a linear, predefined sequence.	"The system prepares, monitors, and analyses reactions sequentially."	0.6
	Environmental Uncertainty	The system operates in a controlled lab setup, with no environmental variability or uncertainty.	"RoboChem operates under stable laboratory conditions without external environmental uncertainty."	0
	Task Variability	The system focuses on optimizing photocatalytic reactions, and its tasks do not extend beyond this domain.	"The platform is designed to handle photocatalysis workflows, with no evidence of broader task variability."	0.2

Supplementary Table 21 ADePT evaluation for "An autonomous laboratory for the accelerated synthesis of novel materials"[13]

Reference	Use case	Robotic System
**Szymanski et al., 2023**	Automating the discovery and synthesis of novel inorganic materials using a robotic platform integrated with machine learning (ML) for decision-making and optimization.	The A-Lab uses three robotic arms: a Mitsubishi RV7-FL for powder handling and two UR5e robots for sample transfers between stations (preparation, heating, and characterization). The robots handle powders, vials, and crucibles autonomously in a solid-state synthesis workflow.

Dimension	Metric	Justification	Paper Evidence	Score

**Adaptability and Learning**	Adaptation Efficiency	Learning is applied to optimize synthesis conditions (via active learning), but robotic operations like sample transfers follow fixed pre-programmed routines.	"Active learning is used for synthesis optimization, not for robotic movement adaptation."	0
	Task Generalization	The robotic system is specialized for solid-state synthesis workflows and does not generalize to other laboratory tasks beyond this domain.	"The A-Lab is specialized for the synthesis of inorganic materials in powder form."	0.2
	Real-Time Adaptability	The robot executes predefined routines without adjusting its actions dynamically during operation.	"Robotic arms follow pre-programmed routines for material handling without real-time adaptability."	0
	Learning Method	Learning is applied to experiment optimization via active learning, but not to the robot's physical operations or sample handling.	"Learning is used for optimizing synthesis procedures, but not for robotic movements."	0
**Dexterity**	Degrees of Freedom (DOF)	The 6-axis UR5e robots provide high dexterity, enabling them to perform tasks like vial transfer, powder handling, and placement in furnaces and XRD stations.	"The A-Lab contains three robotic arms: a Mitsubishi RV7-FL and two 6-axis UR5e robots for sample transfers and handling."	0.7
	Force Control	The robotic system is equipped with force sensors, allowing it to handle delicate items like crucibles with precise force adjustments.	"The UR5e robots use force sensors to handle fragile alumina crucibles."	0.6
	Grasp Variety	The system's grippers are designed to handle vials, crucibles, and powder-dosing tools, but are specialized for these objects, limiting versatility in grasping other types.	"The UR5e robots use custom grippers to handle vials, alumina crucibles, and sample trays."	0.4
**Perception**	Sensor Variety	The system uses force sensors for handling delicate materials and standard sensors for positional feedback but lacks vision or advanced perception capabilities.	"The robotic arms (UR5e) are equipped with force sensors for safe handling of samples."	0.2

	Feedback Integration	Feedback is used for material handling (via force sensors) and material transfer but not for dynamically adjusting actions in real time based on environmental inputs.	"The robots use force feedback to handle crucibles and delicate samples, but no other real-time feedback adjusts the robot's actions."	0.4
	Environmental Awareness	The system operates in a controlled environment with no real-time adaptation to external factors like temperature or humidity changes.	"The laboratory operates in a controlled environment, and there is no indication of real-time environmental awareness."	0.2
**Task Complexity**	Number of Subtasks	The robot handles multiple subtasks, including powder handling, sample transfers, and XRD analysis, but these tasks follow a simple, linear sequence.	"The A-Lab's robotic arms perform sample preparation, heating, and characterization in sequence."	0.6
	Environmental Uncertainty	The system operates in a stable, controlled environment without encountering environmental uncertainties.	"The A-Lab functions in a controlled lab environment with no environmental variability."	0
	Task Variability	The system is designed for tasks related to solid-state synthesis, with no variability beyond this domain.	"The A-Lab handles tasks specifically related to powder synthesis and characterization."	0.2

Supplementary Table 22 ADePT evaluation for "The evolution of Materials Acceleration Platforms: toward the laboratory of the future with AMANDA"[58]

Reference	Use case	Robotic System
**Wagner et al., 2021**	Automating the research process for developing new materials, focusing on solution-processed organic solar cells (OSCs) using the AMANDA platform, which integrates automation, data collection, and AI-based optimization.	The AMANDA platform includes pipetting robots for precise liquid handling, alongside a SCARA transport robot for substrate handling, spin coaters, and optical characterization tools. The pipetting robots prepare and dispense solutions for OSC fabrication.

Dimension	Metric	Justification	Paper Evidence	Score
**Adaptability and Learning**	Adaptation Efficiency	Learning is applied to optimizing process conditions through AI-based optimization, but the robots follow pre-programmed sequences with no task adaptation.	"AMANDA enables AI-based optimization of process parameters, but robotic movements are pre-defined."	0

	Task Generalization	The robotic system is specialized for organic solar cell fabrication and does not generalize to other tasks or workflows.	"The AMANDA system is designed specifically for thin-film devices like organic solar cells."	0.2
	Real-Time Adaptability	The robotic system follows fixed workflows and does not adjust its actions based on real-time feedback or environmental changes.	"Tasks are performed in predefined sequences, from spin coating to measurements."	0
	Learning Method	Learning is applied to experimental conditions via machine learning, but the robot's physical operations are not subject to learning or improvement.	"AI helps optimize spin coating and solution concentrations, but robot handling is fixed."	0
**Dexterity**	Degrees of Freedom (DOF)	The SCARA transport robot has 4 degrees of freedom (DOF), while pipetting robots provide precise liquid handling capabilities, adding flexibility in handling solutions.	"The system includes a SCARA transport robot for substrates and pipetting robots for precise liquid handling."	0.7
	Force Control	No force-sensitive control is mentioned, indicating that the SCARA and pipetting robots handle rigid objects (e.g., substrates) and liquid without requiring precise force adjustments.	"No force-sensitive handling is mentioned in the substrate transfer or liquid handling processes."	0
	Grasp Variety	The SCARA robot is designed for substrates, and pipetting robots are specialized for handling liquids, limiting their versatility beyond these specific tasks.	"The SCARA robot handles substrates for spin coating, while pipetting robots manage solution preparation and dispensing."	0.4
**Perception**	Sensor Variety	The system uses standard sensors for optical characterization (e.g., cameras, UV-Vis spectrometers) and positional feedback for the robots but lacks advanced sensory inputs such as vision or tactile sensors.	"Optical characterization is performed using a camera setup and UV-Vis spectrometer."	0
	Feedback Integration	Feedback is limited to inline process measurements (e.g., optical density), but no dynamic feedback influences the robot's real-time actions	"In-process measurements provide insights into the sample fabrication process, but feedback is used for	0.2

		during substrate transfer or liquid handling.	process evaluation, not robot control."	
	Environmental Awareness	The robots operate in a controlled environment without real-time adaptation to external environmental conditions.	"The platform operates under controlled conditions, including glove boxes for inert processing."	0.2
**Task Complexity**	Number of Subtasks	The robots handle multiple steps in OSC fabrication, including solution preparation, substrate transfer, spin coating, and characterization, but these are linear and predefined tasks.	"AMANDA automates 50 tasks across substrate handling, solution preparation, spin coating, and characterization."	0.6
	Environmental Uncertainty	The system operates in a controlled environment (e.g., glove boxes) with no exposure to environmental uncertainty.	"Experiments are conducted in controlled glove boxes to ensure inert processing."	0
	Task Variability	The SCARA and pipetting robots are specialized for thin-film solar cell fabrication, with little variability in tasks beyond this domain.	"The system is designed to automate thin-film device fabrication, with no broader task variability."	0.2

Supplementary Table 23 ADePT evaluation for "A robotic platform for the synthesis of colloidal nanocrystals"[59]

Reference	Use case	Robotic System
**Zhao et al., 2023**	Automating high-throughput synthesis and morphological control of colloidal nanocrystals (NCs) using a robotic platform integrated with AI-driven inverse design.	The robotic platform includes a SCARA robot mounted on a linear rail (mobile robot) for microplate transport, a 6-axis AUBO robotic arm for equipment service, and automated pipettes for liquid handling.

Dimension	Metric	Justification	Paper Evidence	Score
**Adaptability and Learning**	Adaptation Efficiency	The system applies machine learning for optimizing synthesis conditions but follows fixed robotic protocols, with no real-time adaptation during handling tasks.	"Machine learning optimizes reaction conditions based on feedback, but robotic movements remain pre-programmed."	0
	Task Generalization	The platform is specialized for colloidal nanocrystal synthesis and does not demonstrate generalization to other types of laboratory tasks.	"The robotic platform is designed specifically for the synthesis and characterization of nanocrystals."	0.2
	Real-Time Adaptability	The robots follow fixed workflows and do not	"The platform executes tasks according to	0

		dynamically adjust their actions based on feedback from the characterization modules.	predefined sequences without dynamic adaptation."	
	Learning Method	Machine learning is utilized for optimizing synthesis conditions (inverse design) but does not extend to adapting robotic manipulations.	"ML models are trained to optimize synthesis conditions based on in situ data but do not adapt robotic movements."	0
**Dexterity**	Degrees of Freedom (DOF)	The platform includes a 6-axis AUBO robotic arm and a SCARA robot, providing high dexterity and flexibility for handling microplates, vials, and liquid transfers.	"For the two collaborative robots, the mobile robot for microplate transport is a SCARA robot, and the robotic arm for equipment service is a 6-DoF AUBO arm."	0.7
	Force Control	The robotic arms do not use force-sensitive control, limiting their ability to handle fragile objects or tasks requiring precise grip adjustments.	"No mention of force-sensitive handling capabilities in the synthesis process."	0
	Grasp Variety	The SCARA robot and AUBO arm handle specialized items like microplates, vials, and reagents, but their grasping capabilities are limited to these objects.	"The platform uses a SCARA robot for microplate transport and a 6-axis AUBO arm for equipment service, alongside automated pipettes for precise liquid handling."	0.4
**Perception**	Sensor Variety	The system integrates optical sensors (e.g., spectrometers) and a mobile camera for in situ analysis but lacks advanced tactile or force sensors.	"The platform is equipped with optical characterization modules such as a spectrometer and a colour-ultrasensitive mobile camera."	0
	Feedback Integration	Feedback from optical sensors informs reaction monitoring and synthesis conditions, but it does not directly control the robots' movements dynamically.	"In situ characterization informs the synthesis process, but the robot follows predefined actions based on data without dynamic adjustments."	0.2
	Environmental Awareness	The robotic arms operate in a controlled environment with no sensors monitoring external conditions like humidity or temperature.	"The system operates in a controlled laboratory environment, without real-time environmental awareness."	0.2
**Task Complexity**	Number of Subtasks	The platform handles multiple subtasks, including solution preparation, synthesis, and	"The robotic arms perform tasks like substrate handling, pipetting, and	0.6

		optical characterization, but they follow a linear, predefined sequence.	sample analysis in sequence."	
	Environmental Uncertainty	The platform operates in a stable environment and does not encounter environmental variability or uncertainty.	"Experiments are conducted in a controlled lab environment."	0
	Task Variability	The system is designed specifically for nanocrystal synthesis, limiting its task variability beyond this scope.	"The platform is specialized for colloidal nanocrystal synthesis workflows."	0.2

Supplementary Table 24 ADePT evaluation for "Autonomous, multiproperty-driven molecular discovery: From predictions to measurements and back" [60]

Reference	Use case	Robotic System
**Koscher et al. (2023)**	Automated molecular discovery involving predictive modelling, synthesis, and characterization in a controlled lab environment.	A PreciseFlex SCARA robot (4 DOF) mounted on a linear rail and a Tecan Freedom EVO liquid handler for performing liquid handling and transfer tasks.

Dimensions	Metric	Justification	Evidence	Score
**Adaptability and Learning**	Adaptation Efficiency	The system adapts experimental protocols based on modeling outcomes but does not adapt its robotic movements; these remain fixed, showing limited adaptability in physical actions.	"Adjustments are made in experimental conditions, not in robotic motion paths."	0.2
	Task Generalisation	The platform demonstrates task generalization within chemical synthesis and characterization, using the same robotic setup for different reaction pathways and liquid handling tasks.	"System executes multiple reaction types using the same robotic components."	0.6
	Real-Time Adaptability	The robot lacks real-time adaptability for motion adjustments; tasks follow predefined protocols without adaptation based on sensor data.	"No dynamic adjustments are made; tasks are executed as pre-programmed."	0
	Learning Method	Machine learning is used for experimental predictions and optimizations, but it does not influence robotic motion or adapt robot behaviour in real time.	"Models are employed for optimizing reaction conditions, not robotic behavior."	0
**Dexterity**	Degrees of Freedom	The SCARA robot provides 4 DOF plus linear rail movement, while the liquid handler adds flexibility in liquid dispensing and transfer, suitable for laboratory tasks but limited compared to systems with more DOF.	"SCARA robot handles positioning while the Tecan liquid handler manages multi-channel dispensing."	0.6

	Force Control	No force control or tactile sensing capabilities are included, which limits precise interaction during tasks; all actions are pre-programmed.	N/A	0
	Grasp Variety	The system uses grippers designed for lab-specific items (e.g., vials, plates) and the liquid handler's specialized tools, but it cannot adapt to varied object shapes beyond this.	"Grippers and handlers are configured for specific lab equipment..."	0.4
**Perception**	Sensor Variety	The system relies on proprioceptive sensors (e.g., joint encoders) for the SCARA robot and integrated sensing in the liquid handler but lacks external sensory modalities like vision or force feedback.	"The robot utilizes joint position sensing for movement accuracy..."	0
	Feedback Integration	The system does not utilize feedback mechanisms (e.g., force or visual feedback) to adjust the robotic motion in real time; tasks are executed based on pre-programmed sequences.	"SCARA robot operates along fixed paths... liquid handler operates with pre-configured protocols."	0
	Environmental Awareness	The robot and liquid handler operate within a structured environment with fixed stations and do not dynamically map or adapt to the environment beyond predefined setups.	"Fixed paths and predefined positions are used for all robot operations."	0.2
**Task Complexity**	Number of Subtasks	The robotic system handles complex, multi-step synthesis and measurement workflows, utilizing the SCARA robot and liquid handler for various coordinated tasks, reflecting high complexity.	"System manages multi-step reactions... coordinating robotic arms and liquid handlers."	0.8
	Environmental Uncertainty	The robotic system operates under controlled conditions, and there is no evidence of adaptation to environmental changes or unexpected situations within the setup.	"Fixed laboratory setup with controlled conditions..."	0.2
	Task Variability	The platform autonomously executes various chemical synthesis and measurement tasks, but this variability remains confined to the predefined experimental domain.	"Multiple classes of chemical reactions are automated within the system's domain."	0.6

SM3.2. ADePT Evaluation for Key features of Robot Proficiency Use Cases

Supplementary Table 25 ADePT evaluation for "High-Accuracy Injection Using a Mobile Manipulation Robot for Chemistry Lab Automation"[61]

Reference	Use case	Robotic System
**Angelopoulos et al., 2023**	Automating precise injection tasks in a chemistry laboratory setting using a mobile manipulation robot equipped with a syringe for applications such as gas chromatography (GC).	The platform includes a Fetch Mobile Manipulator equipped with a 7-DoF arm and a differential drive base for mobility. It uses a deep learning-based vision system for high-precision alignment of the syringe needle through visual servoing.

Dimension	Metric	Justification	Paper Evidence	Score
**Adaptability and Learning**	Adaptation Efficiency	The platform uses deep learning for visual recognition and alignment of the syringe needle, effectively adapting the robot's actions in real time based on visual feedback.	"Deep learning is used to localize the syringe needle for visual servoing, and the robot adjusts its actions based on this feedback."	0.4
	Task Generalization	The system is specialized for injection tasks and does not generalize to other types of laboratory activities or robotic manipulation beyond this domain.	"The robot is specifically designed for injection tasks using visual guidance, with no evidence of generalization beyond this task."	0.2
	Real-Time Adaptability	The robot dynamically adjusts its syringe alignment and positioning based on real-time visual feedback, demonstrating effective real-time adaptability for this specific task.	"The method relies on camera feedback to guide alignment and adjust the syringe in real time for precision."	0.6
	Learning Method	The learning is focused on visual recognition and alignment of the syringe needle, not on robotic movement or handling adaptability.	"The deep learning model is trained to detect the syringe's key points, but it does not adaptively change the robot's handling methods."	0
**Dexterity**	Degrees of Freedom (DOF)	The robot uses a 7-DoF arm, which provides high dexterity necessary for precise syringe manipulation and injections within confined spaces.	"The Fetch Mobile Manipulator has a 7-DoF arm that allows for precise positioning of the syringe for injection tasks."	0.7
	Force Control	No force-sensitive control is mentioned, limiting its capability to handle delicate tasks that require precise force application.	"The method relies on visual feedback rather than force feedback to perform the injection task."	0
	Grasp Variety	The robotic arm can grasp and manipulate syringes	"The robot grasps the syringe and performs	0.4

		and vials; however, its gripper is specialized and does not show versatility beyond these items.	injections, but no other types of grasping tasks are described."	
**Perception**	Sensor Variety	The system uses multiple cameras (RGBD and machine vision) for visual servoing and a deep learning model to detect and align the syringe needle but lacks tactile or force feedback.	"The robot uses two Toshiba TeliCams mounted on the chemistry equipment to visually track and align the syringe needle."	0.5
	Feedback Integration	Visual feedback actively guides the robot in positioning the syringe for injections, demonstrating effective integration of visual feedback for real-time adjustments.	"Our approach uses visual servoing based on the needle's localization through the cameras to achieve accurate alignment and injection."	0.6
	Environmental Awareness	The robot operates in a controlled lab environment and lacks sensors for real-time environmental monitoring, such as temperature or air quality.	"The system operates in a controlled environment, focusing solely on visual feedback for task execution."	0.2
**Task Complexity**	Number of Subtasks	The robot handles multiple subtasks like navigating to the target, aligning the syringe, and injecting samples, but these tasks follow a straightforward sequence.	"The robot navigates, aligns the needle, and performs injections using pre-defined steps."	0.6
	Environmental Uncertainty	The platform operates in a stable, controlled environment and does not deal with environmental uncertainties such as fluctuating temperature or airflow.	"Experiments are performed in a controlled lab setup."	0
	Task Variability	The robotic system focuses on syringe-based injection tasks for chemical analysis and does not handle other types of lab activities or tasks beyond this.	"The mobile manipulator is specifically used for injections in chemistry instruments such as gas chromatographs."	0.2

Supplementary Table 26 ADePT evaluation for "A mobile robotic chemist"[12]

Reference	Use case	Robotic System
**Burger et al., 2020.**	Automating the search for improved photocatalysts for	The system features a KUKA mobile manipulator consisting of a 7-axis robotic arm mounted on a

	hydrogen production from water using a mobile robotic platform integrated with Bayesian optimization.	mobile base. It uses laser scanning and touch feedback for precise positioning, and it integrates with a variety of laboratory equipment for sample handling, including liquid and solid dispensing stations.

Dimension	Metric	Justification	Paper Evidence	Score
**Adaptability and Learning**	Adaptation Efficiency	The system employs Bayesian optimization for experiment conditions but does not adapt robotic manipulation dynamically; it follows fixed routines with initial feedback only.	"Bayesian optimization helps optimize experiment parameters, but robot actions remain fixed post-calibration."	0.3
	Task Generalization	The robotic system is focused on photocatalytic experiments but shows potential for generalization with additional hardware integration.	"This modular approach could extend beyond photocatalysis to other research areas with compatible modules."	0.4
	Real-Time Adaptability	The robot uses visual and touch feedback for positioning but does not dynamically adapt during task execution based on other sensory inputs.	"Real-time positioning adjustments are made with touch feedback, but task execution follows a pre-programmed sequence."	0.2
	Learning Method	The platform's learning focuses on optimizing experimental conditions via Bayesian methods rather than robotic manipulation techniques.	"Bayesian optimization is used to refine experiment parameters, not for adapting robotic control."	0
**Dexterity**	Degrees of Freedom (DOF)	The 7-DoF LBR iiwa arm provides high dexterity, enabling the robot to perform complex tasks such as vial handling and cartridge placement with precision.	"The robotic arm has 7 degrees of freedom, allowing it to conduct various manipulations required for laboratory experiments."	0.7
	Force Control	The robot uses touch feedback but lacks force-sensitive control for manipulating objects that require delicate handling.	"The robot enhances its positioning accuracy with touch but does not use force control for handling objects."	0
	Grasp Variety	The gripper is designed for vials, racks, and cartridges, showing versatility within the experimental setup but not beyond these types of objects.	"The multipurpose gripper was developed to handle vials, solid-dispensing cartridges, and racks."	0.5
**Perception**	Sensor Variety	The platform uses laser scanning for navigation and touch sensors for precise	"The KMP uses a map for navigation, and the robot enhances precision using	0.5

		positioning but lacks tactile sensors for handling delicate objects.	a touch routine for fine positioning."	
	Feedback Integration	Feedback from laser scanning and touch sensors improves positioning, but it does not dynamically adjust robotic manipulation during object handling.	"The robot uses touch feedback to precisely locate itself at stations but follows pre-programmed actions afterward."	0.4
	Environmental Awareness	The robot navigates within a controlled laboratory using laser scanners for localization, but it does not monitor environmental conditions such as temperature or humidity.	"The mobile platform is equipped with laser scanners for precise navigation in the lab environment."	0.2
**Task Complexity**	Number of Subtasks	The system performs multiple tasks such as solid dispensing, vial loading, and liquid handling in sequence, but these tasks are highly structured and linear.	"The robot conducts tasks like loading samples and transferring vials sequentially between stations."	0.6
	Environmental Uncertainty	The platform operates in a stable environment with no exposure to dynamic changes or environmental uncertainties.	"Experiments occur in a controlled laboratory environment."	0
	Task Variability	The robot's tasks focus on the specific setup for photocatalytic experiments, limiting its variability beyond this domain.	"The robot's workflow is designed specifically for the search for photocatalysts in the lab environment."	0.3

Supplementary Table 27 ADePT evaluation for "Leveraging Multi-modal Sensing for Robotic Insertion Tasks in R&D Laboratories"[62]

Reference	Use case	Robotic System
**Butterworth et al., 2023**	Automating vial insertion tasks in chemistry labs using a multi-modal robotic system that combines visual, force, and tactile feedback for precise and reliable handling.	The system employs a UR5 robotic arm with a Robotiq 2F-85 gripper, equipped with an Intel D415 camera for visual guidance and DIGIT tactile sensors for enhanced manipulation. The robot uses multi-modal feedback (visual, force, tactile) for vial insertion into racks.

Dimension	Metric	Justification	Paper Evidence	Score
**Adaptability and Learning**	Adaptation Efficiency	The system adapts vial insertion based on feedback from multiple sensors, demonstrating efficiency in refining positioning and adjusting force during insertion tasks.	"The tactile and force feedback methods refine the positioning for more accurate vial placement."	0.6

	Task Generalization	The robotic system is specialized for vial insertion tasks in lab environments and does not generalize to other laboratory procedures beyond vial handling.	"The focus is on vial insertion, with no indication of adaptation for other lab tasks."	0.3
	Real-Time Adaptability	The robot adjusts its movements dynamically using visual and force feedback during vial insertion, demonstrating effective real-time adaptability.	"Multi-modal feedback allows the robot to adjust its actions in real time, improving success rates."	0.7
	Learning Method	The platform does not employ learning techniques like reinforcement learning; instead, it uses a predefined multi-modal feedback system to improve task performance.	"The approach relies on multi-modal sensing rather than machine learning for improving the success rate."	0
**Dexterity**	Degrees of Freedom (DOF)	The 6-DoF UR5 robotic arm provides sufficient dexterity for handling and inserting vials in various positions, offering a high level of manoeuvrability.	"The UR5 robotic arm with 6 degrees of freedom is used for the vial insertion task."	0.6
	Force Control	The system employs force sensors to monitor the insertion process, allowing the robot to detect when the vial is misaligned or obstructed, enhancing precision.	"The robot uses intrinsic force sensors to detect misalignment during insertion and adjust accordingly."	0.6
	Grasp Variety	The robotic arm uses a 2-finger gripper equipped with tactile sensors, suitable for handling vials, but shows limited adaptability beyond these objects.	"The robot uses a two-finger gripper with tactile feedback to grasp and position vials."	0.4
**Perception**	Sensor Variety	The system integrates multiple sensors: an RGBD camera for visual input, intrinsic force sensors for feedback, and tactile sensors on the gripper for precise handling.	"A camera is mounted at the wrist, and two camera-based tactile sensors are attached to the gripper."	0.8
	Feedback Integration	The robot uses multi-modal feedback (visual, force, tactile) to adjust its actions dynamically, improving insertion accuracy and reliability in real time.	"Our multi-modal approach boosts the success rate of the vial insertion to 89.55% by using force and visual feedback."	0.7
	Environmental Awareness	The robot operates in a controlled environment using visual and force feedback but lacks environmental sensors	"The system operates in a simulated lab environment, relying on visual and force	0.6

		for monitoring conditions like temperature or humidity.	sensors for object interaction."	
**Task Complexity**	Number of Subtasks	The system performs tasks like vial pickup, rack detection, and insertion sequentially, with adjustments based on feedback, but the overall sequence remains structured.	"The robot performs sequential tasks from detecting the rack to inserting the vial, guided by feedback sensors."	0.6
	Environmental Uncertainty	The use of visual, tactile, and force feedback mitigates positional uncertainties of vials and racks, ensuring accurate alignment and placement despite minor variations.	"The multi-modal feedback system compensates for positional deviations of objects, improving insertion accuracy."	0.6
	Task Variability	The robot's task scope is limited to vial handling and insertion, lacking flexibility for broader applications in the lab beyond these functions.	"The study focuses exclusively on vial insertion tasks."	0.2

Supplementary Table 28 ADePT evaluation for " ORGANA: A Robotic Assistant for Automated Chemistry Experimentation and Characterization "[63]

Reference	Use case	Robotic System
**Darvish et al., 2024**	Automation of diverse chemistry experiments in laboratory settings with natural language interaction.	The system uses a 7-DoF Franka Emika Panda robotic arm for manipulation tasks, advanced 3D visual perception for transparent object detection, and integrates feedback from lab instruments (e.g., analytical balances, temperature sensors). The TAMP system is integrated to solve task scheduling and motion simultaneously, allowing parallel task execution, which significantly increases lab automation efficiency.

Dimension	Metric	Justification	Paper Evidence	Score
**Adaptability and Learning**	Adaptation Efficiency	LLM interaction allows efficient adaptation based on user instructions and real-time feedback from sensors, enhancing task flexibility.	"ORGANA uses LLMs for symbol grounding and task adaptation based on sensor feedback, providing flexible lab automation".	0.7
	Task Generalization	The TAMP system optimizes task execution across various chemistry experiments, allowing generalization within the chemistry domain. LLM-driven adaptation further enhances flexibility.	"ORGANA supports various chemistry experiments through adaptable LLM-driven instructions and optimized task execution via TAMP".	0.8
	Real-Time Adaptability	The robot dynamically adjusts actions using TAMP, multi-modal feedback (e.g., visual perception,	"The TAMP capability of ORGANA allows real-time task adjustments and	0.8

		temperature), and optimized scheduling, enabling precise real-time corrections and parallel task execution.	parallel task execution, improving efficiency".	
	Learning Method	While the platform uses LLMs for symbolic reasoning and user interaction, it does not employ advanced learning methods like reinforcement learning for task execution.	"The platform relies on LLM-based reasoning without advanced machine learning for manipulation".	0.3
**Dexterity**	Degrees of Freedom (DOF)	The 7-DoF Franka Emika Panda offers high dexterity, supporting precise manipulation and diverse positioning tasks essential for chemistry workflows.	"The robotic arm provides seven degrees of freedom, allowing complex manipulation in the chemistry experiments".	0.7
	Force Control	The robot does not incorporate force sensors but indirectly manages force through analytical balance feedback, limiting precise control in tasks that require direct force sensing.	"No direct force sensors are mentioned; control relies on external equipment feedback such as the balance".	0
	Grasp Variety	The robotic arm's gripper handles standard labware, such as vials and beakers, showing versatility within lab tasks but limited adaptability outside this scope.	"The robot uses a gripper suitable for vials and beakers common in chemistry setups".	0.4
**Perception**	Sensor Variety	The platform integrates visual sensors for object detection and pose estimation, an analytical balance, and temperature sensors, providing a diverse sensory suite.	"ORGANA integrates visual algorithms for transparent object detection and uses balance and temperature sensors for experiment control".	0.6
	Feedback Integration	Visual feedback from object detection, combined with data from lab instruments, enables dynamic adjustments, improving precision during chemistry tasks.	"Feedback from visual perception and lab instruments (temperature and balance) is used to adjust actions dynamically".	0.7
	Environmental Awareness	The robot uses feedback from various lab instruments (e.g., balances, temperature) and advanced visual algorithms for precise object localization, but lacks comprehensive	"The perception system detects and estimates the poses of transparent objects without markers and interacts with the environment based on LLM-driven instructions".	0.7

		environmental monitoring like humidity.		
**Task Complexity**	Number of Subtasks	The TAMP approach allows the robot to handle multiple complex tasks simultaneously, such as pouring, stirring, and monitoring, optimizing the sequence and execution for efficiency.	"ORGANA improves efficiency by solving task and motion planning and scheduling problems simultaneously, enabling parallel execution of tasks".	0.8
	Environmental Uncertainty	Advanced visual perception and pose estimation capabilities help mitigate uncertainties in labware positioning, ensuring precision despite positional variations.	"The perception system accurately detects transparent objects, improving handling precision in uncertain environments".	0.7
	Task Variability	The system shows high variability within chemistry experiments, handling diverse chemistry tasks like solubility testing, recrystallization, and pH monitoring, but remains domain specific.	"ORGANA is utilized to identify the electrochemical characteristics of quinone, a promising molecule for redox flow batteries. The system executes various chemistry tasks".	0.8

Supplementary Table 29 ADePT evaluation for "Autonomous biomimetic solid dispensing using a dual-arm robotic manipulator" [64]

Reference	Use case	Robotic System
**Jiang et al., 2023**	Automating precise solid material dispensing in laboratories through biomimetic robotic manipulation.	Robotic System: Dual-arm ABB YuMi robot employing fuzzy logic control and custom-designed labware (spatulas and vial holders) for solid dispensing tasks.

Dimension	Metric	Justification	Paper Evidence	Score
**Adaptability and Learning**	Adaptation Efficiency	The robot adapts using a fuzzy logic controller based on sensor feedback, but it requires reinitialization for significant deviations, indicating moderate efficiency.	"The fuzzy logic controller adjusts the dispensing motion based on feedback, restarting when necessary to maintain accuracy".	0.6
	Task Generalization	The system can generalize its methods across various solid types but remains specialized to dispensing tasks in laboratory contexts, showing limited generalization.	"The platform supports dispensing of different solid materials by adjusting spatula sizes and dispensing motions".	0.6

	Real-Time Adaptability	The robot adjusts its actions based on real-time feedback from the balance, achieving moderate real-time adaptability for precise task execution.	"Fuzzy logic controls the real-time adjustment of dispensing motions based on balance readings".	0.6
	Learning Method	The system uses a rule-based fuzzy logic approach for real-time adjustments, lacking advanced learning methods like reinforcement learning or neural networks.	"Fuzzy logic control is used for adjusting dispensing motions based on the weight difference and particle size".	0.2
**Dexterity**	Degrees of Freedom (DOF)	The dual-arm robot has 7 degrees of freedom per arm, enabling flexible and precise manipulation suitable for handling various lab tools.	"The dual-arm robot, ABB YuMi, has seven degrees of freedom, enabling precise manipulation of lab tools like spatulas and vials".	0.7
	Force Control	The robot does not have integrated force sensors and relies only on balance feedback, limiting its ability to control force directly during tasks that require precision.	"The balance provides weight feedback; no tactile sensors are integrated for direct force control".	0
	Grasp Variety	The robot's grasp variety is constrained by the use of custom-designed labware (e.g., spatulas and vial holders), limiting its versatility to these specific objects.	"The robot uses custom-designed spatulas and vial holders to manage various dispensing tasks"; Fig. 2 shows the spatulas designed specifically for the robot.	0.4
**Perception**	Sensor Variety	The system uses an analytical balance and visual perception, but it lacks other sensory modalities (e.g., tactile or thermal), limiting its sensing capabilities.	"The analytical balance measures the weight, and visual perception assists with object positioning".	0.2
	Feedback Integration	The robot integrates feedback from the analytical balance and visual perception to dynamically adjust its actions, demonstrating moderate feedback integration.	"The fuzzy logic control algorithm integrates feedback from the balance to adjust dispensing parameters".	0.6
	Environmental Awareness	The robot relies on visual and balance feedback within a controlled environment; it does not perceive or	"Visual algorithms detect and position sample containers, and the balance provides weight feedback".	0.2

		respond to broader environmental factors.		
**Task Complexity**	Number of Subtasks	The robot executes complex sequences involving multiple subtasks such as moving containers, manipulating spatulas, and monitoring weights, demonstrating high task complexity.	"The robot's actions include opening balance doors, placing vials, and manipulating solid hoppers for accurate dispensing".	0.8
	Environmental Uncertainty	The system operates in controlled environments and has limited ability to handle environmental uncertainties, relying primarily on weight and visual feedback.	"The robot executes tasks within a controlled workspace; adjustments are based primarily on weight feedback rather than external environmental factors".	0.4
	Task Variability	The robot can adjust its method to handle various solid types, showing some variability within the domain of solid handling, but remains confined to laboratory tasks.	"Our method adapts to dispense a range of solids by modifying the spatula size and movement patterns according to particle size and type".	0.6

Supplementary Table 30 ADePT evaluation for " Learning Robotic Powder Weighing from Simulation for Laboratory Automation"[65]

Reference	Use case	Robotic System
**Kadokawa et al., 2023**	Automating precise powder weighing tasks in laboratory environments using a reinforcement learning-based approach with domain randomization.	A collaborative robot (UR3) using a dispensing spoon, learning policies trained in simulation with soft actor-critic (SAC) and long short-term memory (LSTM) for real-world powder weighing.

Dimension	Metric	Justification	Paper Evidence	Score
**Adaptability and Learning**	Adaptation Efficiency	The system uses reinforcement learning (SAC with LSTM) for efficient adaptation during tasks, indicating a high level of adaptability.	"The powder weighing manipulation is formulated as a reinforcement learning problem... with an LSTM structure to balance aggressive and conservative actions".	0.8
	Task Generalization	The robot generalizes its weighing policy for multiple powders through domain randomization, but it remains limited to powder-related tasks.	"The policy is trained using domain randomization to adapt to different powder materials and target masses".	0.7
	Real-Time Adaptability	The use of balance feedback and the LSTM-	"The LSTM structure allows the robot to adjust actions	0.7

		based policy supports real-time task adjustments, demonstrating moderate real-time adaptability.	dynamically based on real-time feedback from the balance".	
	Learning Method	The robot employs reinforcement learning (SAC) with domain randomization, representing an advanced learning method for task adaptation.	"We formulate this powder weighing manipulation as a reinforcement learning problem using the soft actor-critic (SAC) algorithm".	0.8
**Dexterity**	Degrees of Freedom (DOF)	The robot (UR3) has 6 degrees of freedom, which is sufficient for the powder weighing task but may not support more complex manipulations outside this scope.	"The experiments used a collaborative robot (Universal Robots, UR3) to control the powder using the dispensing spoon".	0.6
	Force Control	The robot does not use direct force sensors; it depends on balance feedback, limiting its capacity for precise force-based control during tasks.	"The balance provides weight feedback; no tactile or force sensors are integrated".	0
	Grasp Variety	The robot's end effector handles a specialized spoon, and its adaptability to other objects is not demonstrated, indicating limited grasp variety.	"The robot is equipped with a dispensing spoon to scoop and dump powder from a bottle".	0.3
**Perception**	Sensor Variety	The system uses an electric balance and relies on visual feedback; other sensory modalities like tactile sensors are not incorporated.	"The powder weight was measured with an electric balance (ITX220, AS ONE Corp.). The robot controlled the finger-tip pose in hand-chip coordinates at approximately 100 Hz".	0.2
	Feedback Integration	Balance data and visual perception guide dynamic adjustments, showing moderate feedback integration.	"Feedback from the balance and visual perception is integrated to adjust the robot's spoon movement dynamically".	0.6
	Environmental Awareness	The robot uses balance and visual inputs but does not respond to environmental factors beyond these controlled variables, indicating limited awareness.	"The robot relies on visual and balance feedback within a controlled environment; it does not demonstrate broader environmental awareness".	0.2
**Task Complexity**	Number of Subtasks	The robot manages several complex subtasks like	"The robot scoops the powder from the bottle and	0.8

		scooping, shaking, and adjusting powder amounts, showing high task complexity.	performs dumping manipulation using parameterized motions".	
	Environmental Uncertainty	The robot shows limited adaptation to environmental uncertainty, as it operates in a controlled environment, relying mostly on visual and balance feedback.	"Tasks are conducted in a controlled setting; adjustments rely on visual and balance data, without external environmental sensing".	0.4
	Task Variability	The system demonstrates variability by handling different powders and target masses, though it remains specialized within the laboratory domain.	"The robot successfully completes weighing tasks with various powders and target masses (5–15 mg)".	0.7

Supplementary Table 31 ADePT evaluation for " AIR-Chem: Authentic Intelligent Robotics for Chemistry "[66]

Reference	Use case	Robotic System
**Li et al., 2018**	Use Case: Automating laboratory tasks involving precise material handling and environmental modulation using AI-integrated robotics.	Robotic System: Portable multi-robot system consisting of a robotic arm and AGV system equipped with computer vision (CV) and optimization capabilities for laboratory automation.

Dimension	Metric	Justification	Paper Evidence	Score
**Adaptability and Learning**	Adaptation Efficiency	The robot uses visual input and pre-programmed algorithms for efficient adaptation within its controlled environment, indicating moderate adaptation efficiency.	"The system uses CV technology to adjust to object locations dynamically and optimize movement within the workspace".	0.6
	Task Generalization	The robot generalizes tasks related to object handling and positioning within laboratory settings, but it is constrained by the specific equipment and tasks.	"The robotic arm can adapt to different container sizes and types within the workspace, guided by visual feedback".	0.6
	Real-Time Adaptability	The system integrates visual feedback to make adjustments in real-time, but its adaptability is limited to the predefined parameters set by the visual system.	"The CV system allows for real-time adjustments in object handling based on visual data".	0.6
	Learning Method	The system uses rule-based algorithms for object localization and movement but does not	"The platform integrates a parameter optimization module, but it relies on fixed	0.3

		employ advanced learning methods like reinforcement learning.	rules rather than advanced adaptive learning".	
**Dexterity**	Degrees of Freedom (DOF)	When combining the 4 DOF of the robotic arm with the mobile base, the system achieves a higher effective DOF, allowing for flexible movement and manipulation across the workspace.	"The arm features rotation on multiple axes, and the AGV base provides additional mobility, extending the reach and positioning flexibility of the entire system".	0.7
	Force Control	The system does not include force sensors and relies solely on visual feedback for manipulation, limiting precise control in tasks needing direct force feedback.	"There is no mention of integrated force sensors in the robotic setup; control relies on visual feedback".	0
	Grasp Variety	The robotic arm is equipped to handle specific laboratory equipment like beakers, but its grasp variety is limited to these pre-designed objects.	"The robot uses a customized gripper for transferring and holding reaction vessels".	0.3
**Perception**	Sensor Variety	The system uses visual sensors (industrial cameras) and QR code detection, but lacks other sensory modalities like tactile or force sensors.	"The reaction chamber contains an ultrahigh pixel resolution industrial camera for optical characterization... using CV for locating objects".	0.4
	Feedback Integration	Visual feedback is integrated to adjust robotic movements and position objects, providing moderate feedback integration but limited to visual data only.	"The CV system assists in locating objects and adjusting the robotic arm’s actions".	0.6
	Environmental Awareness	The robot adapts to changes based on visual inputs and QR code localization, showing limited awareness beyond controlled visual perception.	"Object recognition and localization are based on QR codes and visual detection via the robot’s camera".	0.4
**Task Complexity**	Number of Subtasks	The robot system performs multiple subtasks such as object recognition, positioning, and manipulation, showing its	"The robotic system includes modules for object detection, transfer, and setup of laboratory environments".	0.6

		capability to manage complex sequences.		
	Environmental Uncertainty	The system operates in a structured environment with controlled conditions and minimal adaptation to unexpected changes, indicating limited handling of uncertainty.	"The robot system is designed for a controlled laboratory setting and does not adapt to unexpected variations outside the structured environment".	0.4
	Task Variability	The robotic platform supports a range of laboratory tasks related to object handling, but it remains specialized to a chemistry-focused environment.	"AIR-Chem adapts to different object handling tasks but is optimized for specific lab setups and processes".	0.3

Supplementary Table 32 ADePT evaluation for " Modular, multi-robot integration of laboratories: an autonomous workflow for solid-state chemistry " [67]

Reference	Use case		Robotic System	
**Lunt et al., 2024**	Use Case: Automating solid-state chemistry processes (e.g., PXRD) with integrated multi-robot systems in a structured laboratory environment.		Robotic System: Integration of the KUKA KMR iiwa mobile manipulator, ABB YuMi dual-arm robot, and Chemspeed platform, orchestrated through the ARChemist software framework.	
**Dimension**	**Metric**	**Justification**	**Paper Evidence**	**Score**
**Adaptability and Learning**	Adaptation Efficiency	The ARChemist system coordinates robots efficiently within defined tasks, but it primarily uses pre-programmed sequences and paths, indicating moderate adaptability within set parameters.	"The system employs predefined sequences and coordination algorithms to manage task transitions among robots, showing moderate flexibility".	0.6
	Task Generalization	The modular setup allows handling various stages of solid-state chemistry tasks but remains specialized within laboratory workflows such as PXRD and sample preparation.	"The system generalizes well within the scope of solid-state chemistry tasks, particularly those related to PXRD workflows".	0.6
	Real-Time Adaptability	The system adjusts its actions based on encoder and laser feedback for navigation but lacks broader real-time adaptability due to the absence of other sensory inputs like vision or force.	"Real-time navigation is supported by encoder feedback and laser scanning, but further adaptability is limited to these modalities".	0.5
	Learning Method	The robotic setup is managed using pre-programmed rules	"The ARChemist platform uses fixed algorithms for	0.2

		and sequences without the application of advanced learning methods such as reinforcement learning or adaptive AI techniques.	managing robotic tasks without employing adaptive or learning-based techniques".	
**Dexterity**	Degrees of Freedom (DOF)	The ABB YuMi robot has 7 DOF per arm, providing high flexibility for manipulating objects, while the KUKA robot adds further mobility with its 7-DOF arm and mobile base.	"The KUKA KMR iiwa has a 7-DOF robotic arm mounted on a mobile platform... The YuMi robot also has two independent 7-DOF arms".	0.8
	Force Control	The system lacks integrated force sensors, relying solely on predefined paths and visual checks; this restricts its ability to adjust based on tactile feedback during manipulation.	"The KUKA and YuMi systems do not include force sensors; adjustments are made based on predefined positional data and encoded paths".	0
	Grasp Variety	The ABB YuMi uses custom grippers suited for handling standard labware, such as vials and PXRD sample holders, limiting its grasp variety to these specific objects.	"The robot is equipped with SmartGrippers and neoprene tape to handle various laboratory containers, but these are tailored for specific labware".	0.5
**Perception**	Sensor Variety	The system uses laser scanners on the mobile KUKA base for navigation and positional tracking, with no mention of visual cameras or other sensors such as force or tactile sensors.	"The KUKA base uses 2D laser scanners to map the environment and navigate through the laboratory".	0.5
	Feedback Integration	The robots integrate feedback primarily through positional encoders and laser scanners for navigation, providing moderate feedback integration limited to positional data.	"Position monitoring is performed using encoders, and laser scanners are utilized for navigating the laboratory space".	0.5
	Environmental Awareness	The system relies on laser scanning for collision avoidance and positional feedback but lacks other forms of environmental sensing, such as vision or temperature monitoring.	"Laser scanners control the proximity to users and obstacles, enabling the robot to stop or adjust paths when necessary".	0.4
**Task Complexity**	Number of Subtasks	The system successfully coordinates multiple robots through a 12-step autonomous PXRD workflow, demonstrating the ability to	"The complete workflow includes 12 coordinated robotic steps involving sample transfer,	0.8

		handle complex, multi-step tasks efficiently.	preparation, and PXRD analysis".	
	Environmental Uncertainty	The robots operate effectively in a structured laboratory environment with known conditions but have limited adaptation to unexpected changes beyond the defined environment.	"Operations are confined to structured settings, and there is no evidence of adaptation capabilities beyond these constraints".	0.3
	Task Variability	The robotic system supports various chemistry tasks related to PXRD and sample preparation, but its applicability is limited to specific procedures and lab equipment.	"The modular configuration is adaptable to several solid-state chemistry procedures but remains focused on predefined laboratory processes".	0.6

Supplementary Table 33 ADePT evaluation for " Robotic Powder Grinding with Audio-Visual Feedback for Laboratory Automation in Materials Science "[68]

**Reference**	**Use case**		**Robotic System**	
**Nakajima et al., 2023**	Use Case: Automating the powder grinding process in laboratory settings for efficient material preparation.		Robotic System: UR5e robotic arm using a multi-modal feedback approach combining audio (ultrasonic microphone) and visual (RGB camera) sensors to control grinding and powder collection.	
**Dimension**	**Metric**	**Justification**	**Paper Evidence**	**Score**
**Perception**	Sensor Variety	The system integrates both visual (RGB camera) and auditory (ultrasonic microphone) sensors, providing diverse sensory input for monitoring powder states.	"The robotic system consists of a UR5e robotic arm, a camera (Intel Realsense D435i), and an ultrasonic sensor positioned under the mortar".	0.4
	Feedback Integration	The integration of audio and visual feedback allows for dynamic adjustments during the grinding process, showing high feedback integration capabilities.	"We propose a novel audio sensing technique to estimate the particle size... combined with visual feedback for efficient grinding".	0.8
	Environmental Awareness	The robot uses multi-modal feedback (audio and visual) to perceive powder distribution and grinding progress, showing reasonable awareness of its immediate environment.	"The grinding progress can be monitored using visual and audio feedback, which provide different qualitative information about the process".	0.4
**Dexterity**	Degrees of Freedom (DOF)	The UR5e arm has 6 DOF, enabling it to perform a variety of precise movements necessary for	"The UR5e robotic arm... performs the grinding and gathering actions with 6 degrees of freedom,	0.6

		grinding and gathering powder effectively.	allowing precise manipulation".	
	Force Control	The system does not include force sensors; it relies on audio and visual feedback for indirect force estimation, limiting precise tactile feedback capabilities.	"No direct force sensors are mentioned; control relies on auditory and visual cues to estimate grinding progress".	0.2
	Grasp Variety	The robotic arm uses specialized tools such as a pestle and a silicone spatula, limiting its adaptability to handle other types of objects beyond these specific tools.	"The system uses a pestle and spatula attached via a soft jig, designed specifically for powder grinding and gathering".	0.4
**Adaptability and Learning**	Adaptation Efficiency	The audio-visual feedback system allows efficient adaptation during tasks, with the robot adjusting its grinding actions based on real-time sensory data.	"The grinding radius and decision-making are based on real-time feedback from the audio and visual systems".	0.7
	Task Generalization	The robot can adapt its grinding technique for different powders and mortar sizes using the feedback system, showing reasonable generalization capabilities within the domain.	"The system is capable of grinding various powder types by adapting its motion parameters based on sensory feedback".	0.6
	Real-Time Adaptability	The robot demonstrates strong real-time adaptability by using audio and visual feedback to dynamically adjust its grinding motion and gathering actions.	"The system monitors grinding progress and decides to continue or gather powder based on the real-time audio and visual signals".	0.8
	Learning Method	The system operates based on rule-based algorithms combining multi-modal feedback rather than employing advanced machine learning methods like reinforcement learning.	"The strategy integrates audio-visual features for efficient manipulation but does not involve machine learning algorithms".	0.3
**Task Complexity**	Number of Subtasks	The system manages multiple complex steps, including grinding, adjusting motion paths, and gathering powder, showing high task complexity management.	"The robot executes a sequence of grinding and gathering steps autonomously, adjusting its actions based on the state of the powder".	0.8
	Environmental Uncertainty	The system effectively adapts to variations in powder distribution within	"The system adjusts grinding parameters based on visual and audio	0.4

		the controlled environment, but it is limited to structured setups and lacks broader uncertainty handling.	feedback but operates within a controlled setting with predefined parameters".	
	Task Variability	The system can adjust to different powder types and conditions, but its application remains confined to the specific task of powder grinding in laboratory settings.	"The robot adapts its grinding approach based on the particle size and distribution, although it is limited to this specific domain".	0.6

Supplementary Table 34 ADePT evaluation for " Precise Well-plate Placing Utilizing Contact During Sliding with Tactile-based Pose Estimation for Laboratory Automation"[69]

Reference	Use case	Robotic System
**Pai et al., 2023**	Use Case: Automating precision insertion of micro well-plates into holders for laboratory experiments with sub-millimeter accuracy using adaptive tactile sensing.	Robotic System: Franka Emika Panda arm (7-DOF) equipped with an adaptive fingers gripper and tactile sensors (GelSight Mini), utilizing visual and tactile feedback for precision insertion tasks.

Dimension	Metric	Justification	Paper Evidence	Score
**Adaptability and Learning**	Adaptation Efficiency	The system's use of tactile feedback for pose estimation and force adjustment allows efficient adaptation during insertion, indicating a high level of adaptability.	"Tactile sensors enable real-time adjustment based on object pose, improving the success rate of the insertion task" .	0.8
	Task Generalization	The robot generalizes well within the specific domain of well-plate handling, but its specialization limits its applicability to other tasks outside this domain.	"The method is optimized for precise placement of well-plates and similar laboratory apparatus but remains focused on this application".	0.6
	Real-Time Adaptability	The robot demonstrates high real-time adaptability by using tactile and force feedback to adjust its actions dynamically during the insertion process.	"The system adjusts based on real-time tactile and force feedback, allowing it to maintain precision even under variable conditions".	0.8
	Learning Method	The system uses rule-based algorithms for control and feedback integration but does not implement advanced learning methods like reinforcement learning.	"The control strategy combines tactile and visual feedback without employing advanced machine learning techniques".	0.3
**Dexterity**	Degrees of Freedom (DOF)	The robotic arm offers 7 DOF, providing high flexibility for complex manipulations	"The Franka Emika Panda arm is equipped with 7 DOF, allowing flexible	0.8

		required during precision insertion.	and precise manipulation of the well-plate during insertion".	
	Force Control	The system incorporates a force/torque sensor for precise control during contact and insertion, indicating strong force control capabilities.	"A Leptrino force/torque sensor is mounted between the robot arm and gripper, enabling precise control based on force feedback".	0.8
	Grasp Variety	The adaptive fingers gripper is specialized for handling well-plates, which limits its versatility to other objects beyond this specific task.	"The gripper design is tailored for micro well-plates, providing stability and precision but limiting adaptability to other object types".	0.4
**Perception**	Sensor Variety	The system integrates tactile (GelSight Mini) and visual sensors (Intel RealSense cameras), providing diverse sensory inputs for precise object manipulation.	"The gripper is equipped with GelSight Mini tactile sensors, and Intel RealSense Depth Cameras are used for object localization".	0.8
	Feedback Integration	The tactile and visual feedback is integrated to adjust the robot's actions dynamically during the insertion process, showing high feedback integration capability.	"The system combines visual data for object detection and tactile feedback for fine adjustment during insertion".	0.8
	Environmental Awareness	The robot uses tactile sensors to detect contact points and align objects during insertion, showing a reasonable level of environmental awareness specific to contact interactions.	"Pose estimation and contact detection are achieved using GelSight sensors, allowing precise control during the insertion task".	0.7
**Task Complexity**	Number of Subtasks	The robotic system manages a complex sequence of actions involving detection, alignment, and insertion, demonstrating its ability to handle multi-step tasks.	"The task involves grasping, pose estimation, alignment, and insertion steps, all coordinated using sensory feedback".	0.8
	Environmental Uncertainty	The system effectively handles uncertainties in object positioning using tactile feedback but is limited to structured environments like laboratory setups.	"Tactile-based pose estimation allows the robot to adjust for inaccuracies in object positioning but within a controlled environment".	0.5
	Task Variability	The system adapts its insertion technique to different well-plate conditions but remains	"The robot adjusts its pose estimation and insertion method for different well-plate	0.6

		confined to well-plate insertion tasks within the laboratory environment.	orientations but is limited to similar laboratory apparatus".	

Supplementary Table 35 ADePT evaluation for " Accelerating Laboratory Automation Through Robot Skill Learning For Sample Scraping "[70]

Reference	Use case	Robotic System
**Pizzuto et al., 2022a**	Use Case: Automating sample scraping tasks in chemistry labs using reinforcement learning (RL) and curriculum learning to improve precision and efficiency.	Robotic System: Franka Emika Panda robot (7-DOF) with Robotiq parallel gripper, integrated with force/torque sensors and RL models (TQC, SAC) for scraping tasks, including curriculum learning for skill acquisition.

Dimension	Metric	Justification	Paper Evidence	Score
**Adaptability and Learning**	Adaptation Efficiency	The RL model, enhanced with curriculum learning, allows efficient adaptation by progressively increasing task complexity, optimizing the scraping process dynamically.	"Curriculum learning is applied to gradually increase the complexity of scraping tasks, enabling the robot to improve its skills progressively".	0.8
	Task Generalization	The robot can adapt its scraping behavior for different vial sizes and conditions using the curriculum-trained policy, but it remains specific to scraping tasks.	"The robot successfully executes scraping across different vial sizes, but the curriculum-trained policy is specialized for this task".	0.6
	Real-Time Adaptability	The system shows strong real-time adaptability by using force/torque feedback to dynamically adjust the scraper's position and maintain optimal contact.	"Real-time adjustments are made based on the force/torque feedback, allowing the robot to maintain contact and adapt its movements dynamically".	0.8
	Learning Method	The system uses model-free reinforcement learning (TQC, SAC) combined with curriculum learning, enhancing skill acquisition and efficiency. Given its advanced methodology, a score between 0.85 and 0.9 is appropriate.	"The robot applies model-free reinforcement learning methods (TQC, SAC) along with curriculum learning for efficient policy development and task mastery".	0.85
**Dexterity**	Degrees of Freedom (DOF)	The Franka Emika Panda arm has 7 DOF, enabling precise and flexible movement for scraping tasks within the vial environment.	"The robotic arm is equipped with 7 degrees of freedom, allowing precise manipulation for scraping operations".	0.8
	Force Control	The system uses a force/torque sensor, enabling precise control of the scraping	"A force/torque sensor is mounted between the robot arm and the	0.8

		tool to maintain contact and prevent damage during the task.	gripper, enabling precise control based on force feedback".	
	Grasp Variety	The robot's gripper is designed to hold a scraper tool but shows limited versatility for adapting to other object types outside the specific scraping task.	"The Robotiq gripper is used with a laboratory scraper, designed for the specific scraping task within the laboratory setting".	0.4
**Perception**	Sensor Variety	The system utilizes force/torque sensors and proprioceptive feedback but lacks visual or tactile sensors, limiting its sensory diversity.	"Force/torque information at the end-effector is used, with no mention of additional visual or tactile sensors".	0.2
	Feedback Integration	The force/torque feedback is integrated for real-time adjustments during the scraping task, showing moderate feedback integration capability.	"The robot adjusts its end-effector position based on force/torque information to maintain contact with the vial wall during scraping".	0.6
	Environmental Awareness	The robot's awareness is limited to force feedback for contact detection; it does not use visual perception or other modalities for broader environmental awareness.	"The scraping process relies on force feedback to detect and maintain contact but lacks broader environmental perception methods".	0.2
**Task Complexity**	Number of Subtasks	The robot manages multiple steps such as insertion, contact maintenance, and powder scraping, showing high capability in handling a sequence of sub-tasks.	"The robotic scraping procedure involves complex steps, including inserting the scraper and maintaining contact with the vial while scraping".	0.8
	Environmental Uncertainty	The system adapts to variations in vial position and size using force feedback, but it is limited to controlled environments where force is the primary variable.	"The robot adjusts its actions based on variations in the force detected during scraping but operates within a structured laboratory environment".	0.5
	Task Variability	The system adapts to different vial conditions and scraper tools but is confined to tasks related to powder scraping within laboratory settings.	"The robot successfully performs scraping with different vial sizes and scraper tools but remains focused on this specific task".	0.6

Supplementary Table 36 ADePT evaluation for " Accelerating Laboratory Automation Through Robot Skill Learning For Sample Scraping "[71]

Reference	Use case	Robotic System
**Pizzuto et al., 2022b**	Use case: Autonomous solubility screening using deep learning for	Robotic system: A Franka Emika Panda robotic arm equipped with an Intel RealSense D435i

	image classification in a laboratory setting.	camera mounted on its end-effector for visual perception.

Dimension	Metric	Justification	Evidence	Score
**Adaptability and Learning**	Adaptation Efficiency	The robot follows pre-programmed movements, and while the visual system adapts for solubility classification, it does not adapt robotic actions based on feedback during operation.	“Robot follows fixed paths for image capture; sensor data do not modify these paths.”	0.2
	Task Generalisation	The system generalizes its solubility screening method across different solvents and solutes using deep learning but lacks generalization capabilities in motion beyond the predefined setup.	“Our architecture... the same setup for various solvents and molecules...”	0.4
	Real-Time Adaptability	The robot lacks real-time adaptability in its motion; the deep learning models process visual data independently of robotic movements, which remain pre-programmed.	“Predefined motion paths for the robotic arm; image processing does not influence these paths.”	0.2
	Learning Method	The deep learning (CNN) method is used only for solubility classification and does not influence robotic behavior, indicating no learning applied to robot adaptation or motion.	“Our system relies on... CNN tailored for autonomous solubility screening”	0
**Dexterity**	Degrees of Freedom	The Franka Emika Panda robot has 7 DOF, which allows it to position the camera effectively for visual tasks but does not engage in complex manipulation tasks.	“The robot perceived its workspace from above the IKA Plate...”	0.7
	Force Control	The system does not include force feedback capabilities; it relies on visual input only, which limits its precision in manipulation tasks like pouring or physical interaction.	N/A	0
	Grasp Variety	The robot arm is designed for visual analysis and positioning rather than diverse manipulation tasks, resulting in limited grasp variety.	N/A	0
**Perception**	Sensor Variety	The system uses a depth camera (Intel RealSense D435i) mounted on the robotic arm for visual input but lacks other	“The robot is equipped with... an Intel RealSense D435i stereo camera	0.1

		sensory modalities (e.g., tactile or force sensing).	mounted on the gripper…”	
	Feedback Integration	Visual feedback from the camera is used to classify solubility, but this information does not adjust the robot's motion in real time; movements are pre-programmed.	“The robot perceived its workspace… using preprogrammed movements…”	0.2
	Environmental Awareness	The robot detects and locates vials using visual data and a mask R-CNN but operates within controlled lab conditions, lacking robust adaptation to dynamic environments.	“Our architecture… detect glass laboratory vials… without need of a human scientist to highlight…”	0.2
**Task Complexity**	Number of Subtasks	The robot is responsible for positioning the camera and capturing images, while the computer vision system handles segmentation and classification; the task complexity remains moderate as it involves these coordinated processes.	“Our architecture is mainly divided into three stages…”	0.4
	Environmental Uncertainty	The system operates under controlled lab conditions and lacks the ability to handle significant changes or variability in the environment.	“Setup was designed… in an unmodified laboratory.”	0.2
	Task Variability	The system performs solubility screening across different solvent and solute combinations but remains constrained to this specific domain without broader task variability.	“We collected data… across six different setups…”	0.4

Supplementary Table 37 ADePT evaluation for " An all-round AI-Chemist with a scientific mind" [72]

Reference	Use case	Robotic System
**Zhu et al. (2022)**	Autonomous chemical synthesis, characterization, and testing in a smart laboratory.	Ridgeback mobile platform with a UR5e robotic arm equipped with a gripper, depth cameras, and LIDAR for laboratory navigation.

Dimension	Metric	Justification	Evidence	Score
**Perception**	Sensor Variety	The system uses LIDAR, depth cameras (Intel RealSense D435i), and a force-feedback gripper, providing diverse sensory inputs for robotic navigation and manipulation.	“The mobile robot…equipped with dual-lidar-based integrated mapping… and depth cameras.”	0.6
	Feedback Integration	The robot integrates sensory data from LIDAR, cameras,	“…real-time interactions between the robot and	0.8

		and gripper force feedback to adjust its path and manipulation tasks in real time, showing a strong degree of integration.	the workstation… for navigation and manipulation…”	
	Environmental Awareness	The robot creates a 2D map using SLAM and utilizes LIDAR and depth cameras to detect and avoid obstacles, demonstrating good awareness of its controlled laboratory environment.	“SLAM algorithm… fuses lidar data to detect surrounding environment information in 360° directions.”	0.8
**Dexterity**	Degrees of Freedom	The mobile manipulator combines the mobility of the Ridgeback base with the 6 DOF of the UR5e arm, resulting in a total of 12 DOF. This allows the robot to reach and interact with various workstations with flexibility.	“The mobile robot…equipped with…six-degree-of-freedom robotic arm… and omni-directional platform.”	0.8
	Force Control	The system features a gripper with force feedback control, providing moderate precision for tasks such as vial grasping and positioning but not delicate manipulation tasks.	“Gripper with grasp force feedback control…ensures precision in experimental operations.”	0.6
	Grasp Variety	The gripper supports grasping various vial types and components necessary for experimental setups, facilitated by vision and force feedback, indicating good grasp variety.	“Gripper…adapted to handle sample vials and experimental equipment.”	0.6
**Adaptability and Learning**	Adaptation Efficiency	The robot autonomously adapts its path and manipulations using SLAM-based navigation and visual feedback, showing moderate efficiency in handling task variations.	“…adaptive Monte Carlo Localization (AMCL)…adjusts robot motion based on experimental tasks.”	0.6
	Task Generalisation	The robotic system generalizes its navigation and manipulation processes to different workstation setups, indicating an ability to perform similar tasks across varied conditions.	“Robot uses SLAM and vision systems for locating workstations and adapting movements.”	0.6
	Real-Time Adaptability	The robot integrates sensory data for real-time adjustments during navigation and manipulation, but its	“Robot…executes operations based on dynamic real-time data from sensors.”	0.6

		adaptability is constrained to pre-programmed sequences within the lab.		
	Learning Method	There is no indication of learning methods applied to robotic behavior. The system uses predefined paths and feedback for navigation and manipulation, relying on external programming.	N/A	0
**Task Complexity**	Number of Subtasks	The robot executes tasks involving multiple steps, such as moving between workstations, positioning vials, and manipulating components, reflecting moderate task complexity.	"The laboratory hosts…auto-synthesis, auto-characterization, and auto-performance-testing…"	0.6
	Environmental Uncertainty	The robot operates in a controlled environment with some ability to adapt to minor changes, such as detecting obstacles and adjusting paths accordingly.	"Laboratory map…using SLAM algorithm for obstacle detection and avoidance."	0.6
	Task Variability	The system performs navigation and manipulation tasks within a controlled lab setup, demonstrating variability within this limited context but not beyond the defined chemical domain.	"Robot…executes different setup tasks based on workstation needs."	0.6

Supplementary Table 38 ADePT evaluation for " A multi-agent-driven robotic AI chemist enabling autonomous chemical research on demand"[73]

Reference	Use case	Robotic System
**Song et al. (2024)**	Autonomous execution of complex, multi-step chemical experiments and optimization within an automated laboratory environment.	A mobile manipulator (Ridgeback platform with a UR5e robotic arm) combined with a linear rail-mounted robotic arm, both equipped with vision systems (LIDAR, depth cameras) and force feedback for navigation and manipulation tasks.

Dimension	Metric	Justification	Evidence	Score
**Adaptability and Learning**	Adaptation Efficiency	The robot system adapts its navigation and manipulation strategies using SLAM and feedback, showing moderate efficiency in adjusting to environmental changes.	"Adjusts paths based on updated SLAM maps and sensory feedback."	0.6
	Task Generalisation	The multi-agent system, leveraging LLMs, allows the robot to generalize across various tasks by dynamically	"Robot Operator agent translates procedures into robotic instructions…"	0.8

		generating and interpreting procedures based on experimental needs.		
	Real-Time Adaptability	The LLMs and multi-agent system provide flexibility, enabling the robot to adapt procedures dynamically based on task requirements, though physical adaptability remains limited by pre-programmed paths.	"Robot Operator agent…adjusts based on real-time feedback to refine paths."	0.6
	Learning Method	The LLM-based multi-agent system actively determines robot behavior by writing and refining code (Code Writer, Code Proofreader) and designing experiments (Protocol Writer, Protocol Critic), showing an advanced integration of learning for robot behavior.	"Code writer…writes Python code leveraging LLM capabilities…Protocol writer generates procedures."	0.6
**Dexterity**	Degrees of Freedom	The mobile manipulator (Ridgeback + UR5e) combined with the linear rail-mounted arm provides 12 DOF for flexible manipulation across a large workspace.	"Two robots: mobile robot with 6 DOF arm and linear rail-mounted arm."	0.8
	Force Control	The grippers on both arms are equipped with force feedback, enabling precise control for grasping and manipulating various chemical containers and lab apparatus.	"Gripper with force feedback control…precision in experimental manipulations."	0.6
	Grasp Variety	The robotic grippers can handle different types of vials and components needed for chemical setups, facilitated by visual and force feedback integration, showing good versatility.	"Adapted to handle vials and experimental equipment."	0.6
**Perception**	Sensor Variety	The system uses LIDAR, depth cameras, and force sensors in both robotic arms, providing varied sensory modalities for navigation, positioning, and manipulation.	"The mobile robot…equipped with dual-lidar… and depth cameras… and gripper force feedback."	0.6
	Feedback Integration	Sensory data from cameras, LIDAR, and force sensors are integrated in real-time to adjust the robot's path and	"Real-time interactions…adjust path and grip based on sensory inputs."	0.8

		manipulation strategies, demonstrating robust integration.		
	Environmental Awareness	The robot uses SLAM to create a map of the laboratory and adapt to obstacles using sensory inputs, showing effective awareness within its environment.	"SLAM algorithm fuses LIDAR data…detects surrounding environment in 360° directions."	0.8
**Task Complexity**	Number of Subtasks	The robotic system handles complex multi-step workflows, with the multi-agent system using LLMs to coordinate robotic operations across workstations, showing high complexity.	"Operates across 20 automated stations…for multi-step workflows."	0.8
	Environmental Uncertainty	The system effectively operates within a controlled lab environment, adapting to obstacles and lab setups with sensory feedback, but struggles with unstructured settings.	"SLAM and obstacle detection within structured lab space."	0.6
	Task Variability	The multi-agent system, using LLMs, enables the robotic system to switch between various tasks (e.g., synthesis, characterization) within the controlled environment, showing high variability.	"Tasks include synthesis, screening, and characterization…"	0.8

Supplementary Table 39 ADePT evaluation for " Go with the flow: deep learning methods for autonomous viscosity estimations"[74]

Reference	Use case	Robotic System
**Walker et al. (2023)**	Autonomous viscosity estimation using dual-arm robotic manipulation and deep learning for fluid motion analysis.	ABB YuMi dual-arm collaborative robot used for automated fluid handling and video acquisition.

Dimension	Metric	Justification	Evidence	Score
**Adaptability and Learning**	Adaptation Efficiency	The robot's adaptability is constrained as it follows pre-programmed sequences, using the captured visual data only for subsequent viscosity estimation but not to adapt the robotic motion.	"The robot follows a sequence… collecting data autonomously."	0.2
	Task Generalisation	The robotic platform generalizes its manipulation skills within the set task of handling different vial types for	"The robot… collects video data of fluid motion…	0.4

		viscosity measurements but is not designed to generalize beyond this scope.	autonomously processes the data.”	
	Real-Time Adaptability	The robot lacks real-time adaptability in its operation, as the motion and data collection are predefined and do not adjust based on real-time sensory inputs.	“The robot rotates the sample... at a pre-defined velocity.”	0.2
	Learning Method	The deep learning model (3D-CNN) is applied for perception (viscosity estimation), not for learning or adapting the robot's physical behavior during tasks.	“3D-CNNs were used to estimate viscosities... from video data.”	0.4
**Dexterity**	Degrees of Freedom	The YuMi robot offers 7 DOF per arm, providing sufficient dexterity for fluid handling tasks such as vial manipulation and rotation for video capture.	“YuMi collaborative robot... a dual-arm, seven-degree-of-freedom platform.”	0.7
	Force Control	The system does not mention any force feedback capabilities or force control mechanisms in the robotic arms, limiting precision in manipulation tasks.	N/A	0
	Grasp Variety	The robot’s grippers handle vials effectively for the viscosity measurements, but there is no mention of handling different object types or shapes beyond the predefined vial setup.	“The robot... picks up the sample from a rack... rotates the sample for video capture.”	0.4
**Perception**	Sensor Variety	The system uses cameras to capture video data of fluid motion but lacks other sensory modalities (e.g., LIDAR, force sensors), indicating a limited range of sensory inputs.	“The robotic setup comprised... a Logitech webcam... placed within the workspace of the robot.”	0.1
	Feedback Integration	Visual data captured by cameras is used to inform the model for viscosity classification, but it does not directly influence real-time robotic motion or adjustments.	“The robot rotates the sample through 90°... capturing video data for analysis.”	0.2
	Environmental Awareness	The robot's environment awareness is limited to the workspace setup and the location of vials, as it follows pre-defined paths without adjusting to dynamic environmental changes.	“The robot picks a sample... moves it to a camera station... following pre-defined sequences.”	0.2
**Task Complexity**	Number of Subtasks	The robot completes a sequence of subtasks,	“An overview of the sequence... moves the	0.6

		including vial picking, rotation for video capture, and storage, reflecting moderate complexity but within a structured setup.	vial from rack to camera and back.”	
	Environmental Uncertainty	The system operates within a controlled lab environment with no adaptability to significant changes or unstructured conditions.	“The setup… involves fixed racks and camera stations.”	0.2
	Task Variability	The robot performs repeated tasks with different samples but remains limited to the task of fluid motion analysis for viscosity estimation, showing low variability.	“The robot tests samples… rotating them at pre-defined velocities.”	0.4

Supplementary Table 40 ADePT evaluation for " Go with the flow: deep learning methods for autonomous viscosity estimations"[74]

Reference	Use case	Robotic System
**Yoshikawa et al. (2022)**	Automation of chemistry experiments using task and motion planning with collision and spillage constraints.	An 8-DOF Franka Emika Panda robotic arm equipped with a Robotiq gripper, RealSense camera for perception, and a scale for feedback integration.

Dimension	Metric	Justification	Evidence	Score
**Adaptability and Learning**	Adaptation Efficiency	The robot refines its motion plans based on visual and weight feedback, particularly in pouring tasks, but the system heavily relies on predefined task sequences rather than adaptive learning.	“Task execution is refined using feedback from perception modules… and object position updates.”	0.6
	Task Generalisation	The task planning system, using PDDLStream, allows the robot to generalize across similar chemistry tasks (e.g., pouring and transferring), demonstrating flexibility based on task constraints.	“PDDLStream generates action sequences… covering picking, moving, and pouring.”	0.8
	Real-Time Adaptability	The system demonstrates real-time adaptability by using the scale's feedback to adjust pouring actions dynamically, ensuring accuracy, although it is restricted to predefined tasks.	“The feedback from the scale is used to adjust pouring actions… in real time.”	0.8
	Learning Method	The system uses PDDLStream for task and motion planning, but there is no evidence of machine learning being applied for robotic behavior adaptation,	“PDDLStream… generates a sequence of actions… for robot execution.”	0

		indicating reliance on pre-programmed routines.		
**Dexterity**	Degrees of Freedom	The robotic system uses an 8-DOF arm, providing increased dexterity for complex maneuvers like pouring and transferring materials while ensuring precision to prevent spillage.	“We introduced an additional (8th) degree of freedom… to increase the success rate of motion planning.”	0.8
	Force Control	There is no evidence of force control mechanisms, such as tactile or force sensors, to assist with precise manipulation tasks, which limits adaptability during interactions.	N/A	0
	Grasp Variety	The Robotiq gripper is utilized for basic grasping tasks (e.g., holding beakers and vessels), but it is not designed to handle a wide variety of object shapes or types beyond these.	“The robot… picks up and manipulates beakers… using the Robotiq 2F-85 gripper.”	0.4
**Perception**	Sensor Variety	The system uses a depth camera (Intel RealSense D435i) and a scale for weight feedback, providing both visual and weight-based sensory inputs, but lacks other modalities like force or tactile sensing.	“The robot is equipped with… an Intel RealSense D435i stereo camera mounted on the gripper…”	0.6
	Feedback Integration	Visual feedback from the camera and weight feedback from the scale are integrated in real time to adjust the robot’s actions, especially during pouring tasks, demonstrating effective feedback integration.	“The feedback from the scale is used to adjust pouring actions… ensuring precise liquid transfer.”	0.8
	Environmental Awareness	The robot’s environmental awareness is limited to object detection using fiducial markers and depth camera data, without dynamic mapping or adaptation beyond predefined settings.	“Perception updates the scene description by detecting objects and estimating positions using markers.”	0.4
**Task Complexity**	Number of Subtasks	The system completes complex workflows involving multiple steps, such as picking, pouring, and transferring, while adhering to constraints like avoiding spillage, indicating high task complexity.	“Our planning framework can conduct… fundamental chemical experiments involving multi-step tasks.”	0.8
	Environmental Uncertainty	The system handles environmental uncertainty	“Constrained motion planning… prevents	0.4

		within a controlled lab environment, using predefined constraints and visual feedback to prevent spillage and ensure safety.	spillage when transporting liquids and powders."	
	Task Variability	The robot autonomously performs different chemistry tasks (e.g., pouring liquids, recrystallization) within the structured lab environment, demonstrating variability within this limited domain.	"The robot performs various pouring skills... solubility and recrystallization experiments."	0.6

SM4. Summary Average Scorings

Supplementary Table 41 ADePT average scorings

Reference	Adaptability & Learning	Dexterity	Perception	Task Complexity
Asano et al., 2023	0.05	0.367	0.233	0.2
Chen et al., 2024	0.1	0.517	0.267	0.2
Christensen et al., 2021	0.05	0.133	0.133	0.2
Coley et al., 2019	0.1	0.333	0.133	0.267
Dembski et al., 2023	0.1	0.6	0.267	0.333
Duke et al., 2024	0.05	0.267	0.133	0.2
Fleischer et al., 2021	0.05	0.4	0.067	0.2
Gongora et al., 2020	0.05	0.267	0.133	0.2
Knobbe et al., 2022	0.1	0.733	0.333	0.333
Lim et al., 2021	0.05	0.333	0.133	0.267
MacLeod et al., 2020	0.05	0.367	0.133	0.267
MacLeod et al., 2022	0.05	0.367	0.133	0.267
Nambiar et al., 2022	0.05	0.35	0.2	0.267
Ozgulbas et al., 2023	0.05	0.333	0.167	0.267
Shiri et al., 2021	0.05	0.367	0.167	0.267
Slattery et al., 2024	0.05	0.167	0.133	0.267
Szymanski et al., 2023	0.05	0.567	0.267	0.267
Wagner et al., 2021	0.05	0.367	0.133	0.267
Zhao et al., 2023	0.05	0.367	0.133	0.267
Koscher et al., 2023	0.2	0.333	0.067	0.533
Angelopoulos et al., 2023	0.3	0.367	0.433	0.267
Burger et al., 2020	0.225	0.4	0.367	0.3
Butterworth et al., 2023	0.4	0.533	0.7	0.467
Darvish et al., 2024	0.65	0.367	0.667	0.767
Jiang et al., 2023	0.5	0.367	0.333	0.6
Kadokawa et al., 2023	0.75	0.3	0.333	0.633
Li et al., 2018	0.525	0.333	0.467	0.433
Lunt et al., 2024	0.475	0.433	0.467	0.567
Nakajima et al., 2023	0.6	0.4	0.533	0.6

Pai et al., 2023	0.625	0.667	0.767	0.633
Pizzuto et al., 2022a	0.763	0.667	0.333	0.633
Pizzuto et al., 2022b	0.2	0.233	0.167	0.333
Zhu et al., 2022	0.45	0.667	0.733	0.6
Song et al., 2024	0.65	0.667	0.733	0.733
Walker et al., 2023	0.3	0.367	0.167	0.4
Yoshikawa et al., 2022	0.55	0.4	0.6	0.6
**Mean**	0.259	0.408	0.310	0.386
**Median**	0.1	0.367	0.25	0.2835

SM5. Supplementary References

1. Wu, J., Jin, Z., Liu, A., Yu, L. & Yang, F. A survey Of learning-Based control of robotic visual servoing systems. *J Franklin Inst* **359**, 556–577 (2022).

2. Suomalainen, M., Karayiannidis, Y. & Kyrki, V. A survey of robot manipulation in contact. *Rob Auton Syst* **156**, 104224 (2022).

3. AboZaid, Y. A., Aboelrayat, M. T., Fahim, I. S. & Radwan, A. G. Soft robotic grippers: A review on technologies, materials, and applications. *Sens Actuators A Phys* **372**, 115380 (2024).

4. Xie, Z., Liang, X. & Roberto, C. Learning-based robotic grasping: A review. *Front Robot AI* **10**, 1038658 (2023).

5. Kumar, V. & Michael, N. Opportunities and challenges with autonomous micro aerial vehicles. *Int J Rob Res* **31**, 1279–1291 (2012).

6. Lee, T., Kwon, J., Wensing, P. M. & Park, F. C. Robot Model Identification and Learning: A Modern Perspective. *Annu Rev Control Robot Auton Syst* **7**, 311–334 (2024).

7. Garrett, C. R. *et al.* Integrated Task and Motion Planning. *Annu Rev Control Robot Auton Syst* **4**, 265–293 (2021).

8. O'Neill, A. *et al.* Open X-Embodiment: Robotic Learning Datasets and RT-X Models : Open X-Embodiment Collaboration 0. in *2024 IEEE International Conference on Robotics and Automation (ICRA)* 6892–6903 (IEEE, 2024). doi:10.1109/ICRA57147.2024.10611477.

9. Brohan, A. *et al.* RT-1: Robotics Transformer for Real-World Control at Scale. (2022) doi:10.15607/rss.2023.xix.025.

10. Kroemer, O., Niekum, S. & Konidaris, G. A Review of Robot Learning for Manipulation: Challenges, Representations, and Algorithms. *Journal of Machine Learning Research* **22**, 1–82 (2020).

11. Tom, G. *et al.* Self-Driving Laboratories for Chemistry and Materials Science. *Chem Rev* **124**, 9633–9732 (2024).

12. Burger, B. *et al.* A mobile robotic chemist. *Nature* **583**, 237–241 (2020).

13. Szymanski, N. J. *et al.* An autonomous laboratory for the accelerated synthesis of novel materials. *Nature* **624**, 1–6 (2023).

14. Dai, T. *et al.* Autonomous mobile robots for exploratory synthetic chemistry. *Nature* **635**, 890–897 (2024).

15. Le, H., Saeedvand, S. & Hsu, C. C. A Comprehensive Review of Mobile Robot Navigation Using Deep Reinforcement Learning Algorithms in Crowded Environments. *Journal of Intelligent and Robotic Systems: Theory and Applications* **110**, 1–22 (2024).

16. Brunke, L. *et al.* Safe Learning in Robotics: From Learning-Based Control to Safe Reinforcement Learning. *Annu Rev Control Robot Auton Syst* **5**, 411–444 (2022).

17. Tang, C. *et al.* Deep Reinforcement Learning for Robotics: A Survey of Real-World Successes. *Annu Rev Control Robot Auton Syst* **8**, 153–188 (2025).

18. Chi, C. *et al.* Diffusion policy: Visuomotor policy learning via action diffusion. *Int J Rob Res* (2025) doi:10.1177/02783649241273668.

19. Chen, L. *et al.* Decision Transformer: Reinforcement Learning via Sequence Modeling. in *Advances in Neural Information Processing Systems* vol. 18 15084–15097 (2021).

20. Yu, C. & Wang, P. Dexterous Manipulation for Multi-Fingered Robotic Hands With Reinforcement Learning: A Review. *Front Neurorobot* **16**, 861825 (2022).

21. Liu, Y., Hou, J., Li, C. & Wang, X. Intelligent Soft Robotic Grippers for Agricultural and Food Product Handling: A Brief Review with a Focus on Design and Control. *Advanced Intelligent Systems* **5**, 2300233 (2023).

22. Weinberg, A. I., Shirizly, A., Azulay, O. & Sintov, A. Survey of learning-based approaches for robotic in-hand manipulation. *Front Robot AI* **11**, 1455431 (2024).

23. Stradovnik, S. & Hace, A. Task-Oriented Evaluation of the Feasible Kinematic Directional Capabilities for Robot Machining. *Sensors* **22**, 4267 (2022).

24. Sereinig, M., Manzl, P. & Gerstmayr, J. Task-Dependent Comfort Zone, a Base Placement Strategy for Mobile Manipulators Based on Manipulability Measures. *Robotics* **13**, 122 (2024).

25. Wong, C.-C., Tsai, C.-Y., Lai, Y.-C. & Wong, S.-W. Manipulability-Aware Task-Oriented Grasp Planning and Motion Control with Application in a Seven-DoF Redundant Dual-Arm Robot. *Electronics (Basel)* **13**, 5025 (2024).

26. Ortenzi, V., Stolkin, R., Kuo, J. & Mistry, M. Hybrid motion/force control: a review. *Advanced Robotics* **31**, 1102–1113 (2017).

27. Abu-Dakka, F. J. & Saveriano, M. Variable Impedance Control and Learning—A Review. *Front Robot AI* **7**, 590681 (2020).

28. Haddadin, S. & Shahriari, E. Unified force-impedance control. *Int J Rob Res* **43**, 2112–2141 (2024).

29. Caramelli, D. *et al.* Discovering New Chemistry with an Autonomous Robotic Platform Driven by a Reactivity-Seeking Neural Network. *ACS Cent Sci* **7**, 1821–1830 (2021).

30. Bayley, O., Savino, E., Slattery, A. & Noël, T. Autonomous chemistry: Navigating self-driving labs in chemical and material sciences. *Matter* **7**, 2382–2398 (2024).

31. Li, H. *et al.* See, Hear, and Feel: Smart Sensory Fusion for Robotic Manipulation. *Proc Mach Learn Res* **205**, 1368–1378 (2022).

32. Mao, Q., Liao, Z., Yuan, J. & Zhu, R. Multimodal tactile sensing fused with vision for dexterous robotic housekeeping. *Nat Commun* **15**, 6871 (2024).

33. Al-Tawil, B., Hempel, T., Abdelrahman, A. & Al-Hamadi, A. A review of visual SLAM for robotics: evolution, properties, and future applications. *Front Robot AI* **11**, 1347985 (2024).

34. Guan, J., Hao, Y., Wu, Q., Li, S. & Fang, Y. A Survey of 6DoF Object Pose Estimation Methods for Different Application Scenarios. *Sensors* **24**, 1076 (2024).

35. Luo, S., Bimbo, J., Dahiya, R. & Liu, H. Robotic tactile perception of object properties: A review. *Mechatronics* **48**, 54–67 (2017).

36. Tang, Q., Liang, J. & Zhu, F. A comparative review on multi-modal sensors fusion based on deep learning. *Signal Processing* **213**, 109165 (2023).

37. Angelopoulos, A., Cahoon, J. F. & Alterovitz, R. Transforming science labs into automated factories of discovery. *Sci Robot* **9**, 6991 (2024).

38. Kurniawati, H. Partially Observable Markov Decision Processes and Robotics. *Annu Rev Control Robot Auton Syst* **5**, 253–277 (2022).

39. Heo, M., Lee, Y., Lee, D. & Lim, J. J. FurnitureBench: Reproducible real-world benchmark for long-horizon complex manipulation. *Int J Rob Res* **44**, 1863–1891 (2025).

40. Zhang, X. *et al.* Material intelligence by the convergence of artificial intelligence and robotic platforms. *Nexus* **2**, 100083 (2025).

41. Tobias, A. V. & Wahab, A. Autonomous 'self-driving' laboratories: a review of technology and policy implications. *R Soc Open Sci* **12**, (2025).

42. Asano, Y., Yoneda, S., Kitai, K., Okada, K. & Shiomi, J. Flexible Laboratory Automation System Based on Distributed Framework: Implementation for Press Process in Polymer Materials Development. in *2023 IEEE 19th International Conference on Automation Science and Engineering (CASE)* vols 2023-Augus 1–6 (IEEE, 2023).

43. Chen, J. *et al.* Navigating phase diagram complexity to guide robotic inorganic materials synthesis. *Nature Synthesis* **3**, 606–614 (2024).

44. Christensen, M. *et al.* Data-science driven autonomous process optimization. *Commun Chem* **4**, 112 (2021).

45. Coley, C. W. *et al.* A robotic platform for flow synthesis of organic compounds informed by AI planning. *Science (1979)* **365**, (2019).

46. Dembski, S. *et al.* Establishing and testing a robot-based platform to enable the automated production of nanoparticles in a flexible and modular way. *Scientific Reports 2023 13:1* **13**, 1–10 (2023).

47. Duke, R. *et al.* ExpFlow: a graphical user interface for automated reproducible electrochemistry. *Digital Discovery* **3**, 163–172 (2024).

48. Fleischer, H. *et al.* Dual-arm Robotic Compound-oriented Measurement System: Integration of a Positive Pressure Solid Phase Extraction Unit. in *2021 IEEE International Instrumentation and Measurement Technology Conference (I2MTC)* vols 2021-May 1–6 (IEEE, 2021).

49. Gongora, A. E. *et al.* A Bayesian experimental autonomous researcher for mechanical design. *Sci Adv* **6**, (2020).

50. Knobbe, D., Zwirnmann, H., Eckhoff, M. & Haddadin, S. Core Processes in Intelligent Robotic Lab Assistants: Flexible Liquid Handling. in *2022 IEEE/RSJ International Conference on Intelligent Robots and Systems (IROS)* vols 2022-Octob 2335–2342 (IEEE, 2022).

51. Lim, J. X. Y., Leow, D., Pham, Q. C. & Tan, C. H. Development of a Robotic System for Automatic Organic Chemistry Synthesis. *IEEE Transactions on Automation Science and Engineering* **18**, 2185–2190 (2021).

52. MacLeod, B. P. *et al.* Self-driving laboratory for accelerated discovery of thin-film materials. *Sci Adv* **6**, (2020).

53. MacLeod, B. P. *et al.* A self-driving laboratory advances the Pareto front for material properties. *Nat Commun* **13**, 995 (2022).

54. Nambiar, A. M. K. *et al.* Bayesian Optimization of Computer-Proposed Multistep Synthetic Routes on an Automated Robotic Flow Platform. *ACS Cent Sci* **8**, 825–836 (2022).

55. Ozgulbas, D. Y. *et al.* Robotic pendant drop: containerless liquid for μs-resolved, AI-executable XPCS. *Light Sci Appl* **12**, 1–10 (2023).

56. Shiri, P. *et al.* Automated solubility screening platform using computer vision. *iScience* **24**, 102176 (2021).

57. Slattery, A. *et al.* Automated self-optimization, intensification, and scale-up of photocatalysis in flow. *Science (1979)* **383**, (2024).

58. Wagner, J. *et al.* The evolution of Materials Acceleration Platforms: toward the laboratory of the future with AMANDA. *J Mater Sci* **56**, 16422–16446 (2021).

59. Zhao, H. *et al.* A robotic platform for the synthesis of colloidal nanocrystals. *Nature Synthesis* **2**, 505–514 (2023).

60. Koscher, B. A. *et al.* Autonomous, multiproperty-driven molecular discovery: From predictions to measurements and back. *Science (1979)* **382**, (2023).

61. Angelopoulos, A., Verber, M., McKinney, C., Cahoon, J. & Alterovitz, R. High-Accuracy Injection Using a Mobile Manipulation Robot for Chemistry Lab Automation. in *2023 IEEE/RSJ International Conference on Intelligent Robots and Systems (IROS)* 10102–10109 (IEEE, 2023). doi:10.1109/iros55552.2023.10341743.

62. Butterworth, A., Pizzuto, G., Pecyna, L., Cooper, A. I. & Luo, S. Leveraging Multi-modal Sensing for Robotic Insertion Tasks in R&D Laboratories. in *IEEE International Conference on Automation Science and Engineering* vols 2023-Augus 1–8 (IEEE, 2023).

63. Darvish, K. *et al.* ORGANA: A robotic assistant for automated chemistry experimentation and characterization. *Matter* **8**, 101897 (2025).

64. Jiang, Y. *et al.* Autonomous biomimetic solid dispensing using a dual-arm robotic manipulator. *Digital Discovery* (2023) doi:10.1039/D3DD00075C.

65. Kadokawa, Y., Hamaya, M. & Tanaka, K. Learning Robotic Powder Weighing from Simulation for Laboratory Automation. in *2023 IEEE/RSJ International Conference on Intelligent Robots and Systems (IROS)* 2932–2939 (IEEE, 2023). doi:10.1109/IROS55552.2023.10342463.

66. Li, J. *et al.* AIR-Chem: Authentic Intelligent Robotics for Chemistry. *J Phys Chem A* **122**, 9142–9148 (2018).

67. Lunt, Amy. M. *et al.* Modular, multi-robot integration of laboratories: an autonomous workflow for solid-state chemistry. *Chem Sci* **15**, 2456–2463 (2024).

68. Nakajima, Y. *et al.* Robotic Powder Grinding with Audio-Visual Feedback for Laboratory Automation in Materials Science. in *2023 IEEE/RSJ International Conference on Intelligent Robots and Systems (IROS)* 8283–8290 (IEEE, 2023). doi:10.1109/IROS55552.2023.10341526.

69. Pai, S. *et al.* Laboratory Automation: Precision Insertion with Adaptive Fingers utilizing Contact through Sliding with Tactile-based Pose Estimation. *ArXiv* (2023).

70. Pizzuto, G. *et al.* Accelerating Laboratory Automation Through Robot Skill Learning For Sample Scraping. (2022).

71. Pizzuto, G., De Berardinis, J., Longley, L., Fakhruldeen, H. & Cooper, A. I. SOLIS: Autonomous Solubility Screening using Deep Neural Networks. in *2022 International Joint Conference on Neural Networks (IJCNN)* vols 2022-July 1–7 (IEEE, 2022).

72. Zhu, Q. *et al.* An all-round AI-Chemist with a scientific mind. *Natl Sci Rev* **9**, (2022).

73. Song, T. *et al.* A multi-agent-driven robotic AI chemist enabling autonomous chemical research on demand. (2024) doi:10.26434/CHEMRXIV-2024-W953H-V2.

74. Walker, M., Pizzuto, G., Fakhruldeen, H. & Cooper, A. I. Go with the flow: deep learning methods for autonomous viscosity estimations. *Digital Discovery* **2**, 1540–1547 (2023).
